# Mechanochemical Sequential Deoxygenative Cross-Coupling Reactions of Phenols Under Ruthenium-Nickel Catalysis

**DOI:** 10.3390/molecules30081835

**Published:** 2025-04-19

**Authors:** Satenik Mkrtchyan, Vishal B. Purohit, Michał Jakubczyk, Vaibhav D. Prajapati, Ronak V. Prajapati, Michael G. Garcia, Eugene Karpun, Vitaliy Yepishev, Manoj K. Saini, Sehrish Sarfaraz, Khurshid Ayub, Gabriela Addová, Juraj Filo, Viktor O. Iaroshenko

**Affiliations:** 1 Department of Chemistry, Faculty of Natural Sciences, Matej Bel University, Tajovskeho 40, 974 01 Banska Bystrica, Slovakia; 2 Department of Chemical Sciences, P. D. Patel Institute of Applied Sciences, Charotar University of Science and Technology (CHARUSAT), Changa 388421, Gujarat, India; vishalpurohit113@gmail.com; 3 Institute of Inorganic Chemistry, Czech Academy of Sciences, Husinec-Rez c.p. 1001, 250 68 Husinec-Rez, Czech Republic; jakubczyk@iic.cas.cz; 4 Shri Alpesh N. Patel Post Graduate Institute of Science & Research, Anand 388001, Gujarat, India; vaibhavdprajapati@gmail.com; 5 Department of Chemistry, Sardar Patel University, Vallabh Vidyanagar 388120, Gujarat, India; ronak71296@gmail.com; 6 Department of Biology/Chemistry, Center for Cellular Nanoanalytics (CellNanOs), University Osnabruck, Barbarastr. 7, D-49076 Osnabruck, Germany; michaelonline89@outlook.com; 7 Life Chemicals Ukraine, Winston Churchill St. 5, 02000 Kyiv, Ukraine; ekarpun@yahoo.com (E.K.); yepishev@gmail.com (V.Y.); 8 Professional Medical and Pharmaceutical College (IAPM), Frometivska, 2, 03039 Kyiv, Ukraine; 9 Department of Chemistry, Institute of Science, Banaras Hindu University, Varanasi 221005, Uttar Pradesh, India; mksaini@bhu.ac.in; 10 Department of Chemistry, COMSATS University, Abbottabad Campus, Abbottabad 22060, KPK, Pakistan; sehrishsarfaraz555@gmail.com (S.S.), khurshid@cuiatd.edu.pk (K.A.); 11 Department of Organic Chemistry, Faculty of Natural Sciences, Comenius University in Bratislava, Ilkovicova 6, 842 15 Bratislava, Slovakia; gabriela.addova@uniba.sk (G.A.); juraj.filo@uniba.sk (J.F.); 12 Functional Materials Group, Gulf University for Science and Technology, Mubarak Al-Abdullah, Kuwait 32093, Kuwait; 13 Centre of Research Impact and Outcome, Chitkara University Institute of Engineering and Technology, Chitkara University, Rajpura 140401, Punjab, India; 14 Georgian American University, School of Medicine, 10 Merab Aleksidze Str., Tbilisi 0160, Georgia

**Keywords:** mechanochemistry, phenols, ruthenium, borylation, Suzuki–Miyaura-type cross-coupling

## Abstract

Herein, we report the first mechanochemical strategy for the Ru-catalyzed deoxygenative borylation of free phenols via C–O bond cleavage. This Ru-catalyzed phenolic borylation approach has been successfully extended to the Suzuki–Miyaura-type cross-coupling of phenols with aryl bromides. The protocol accepts a wide scope of phenolic substrates, allowing the synthesis of aryl pinacolboranes and biphenyl structures in excellent yields and serving as a better alternative to classical cross-coupling reactions in the context of pot, atom, and step economy synthesis.

## 1. Introduction

Phenols are a unique class of basic aromatics present in many natural products and widely used as the starting materials for the synthesis of many pharmaceuticals, agrochemicals, and other value-added chemicals [1,2,3]. Compared with aryl halides, which are common aromatic feedstocks at present, phenols are considered comparatively sustainable and renewable aromatic feedstocks because of their wide availability from lignin biomasses and low cost of production. Using phenols, direct cross-coupling reactions involving phenolic –OH groups would be environmentally benign, with water as the sole byproduct [3,4,5].

**Figure 1:**
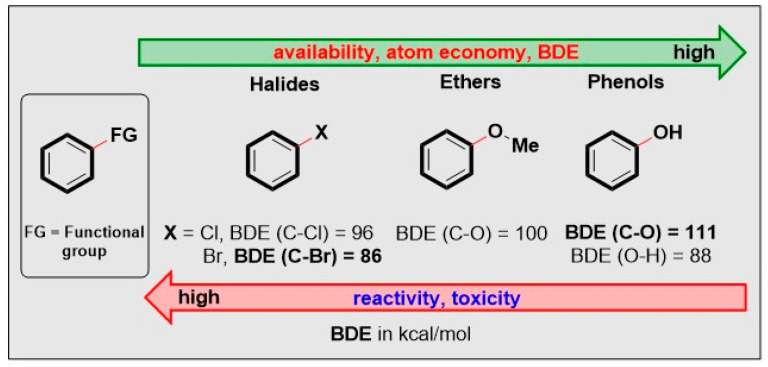
Comparison of BDE of C_Ar_–FG bond in haloarenes, ethers, and phenols.

Given the importance of phenolic feedstocks, chemists have made significant efforts to achieve the deoxygenative functionalization of phenols via cleavage of phenolic C(*sp*^2^)–O bonds [2,3,6,7]. However, the direct activation/cleavage of C(*sp*^2^)–OH bonds, though apparently straightforward, remains greatly challenging, because the phenolic–OH group is highly reactive and has a strong C(*sp*^2^)–O bond with high bond dissociation energy (BDE = 111 kcal/mol) due to *p–π* conjugation compared to that of the O–H bond (BDE = 88 kcal/mol) (Figure 1) [3,8]. Thus, the oxidative addition to transition metals by the strong C(*sp*^2^)–O bond of phenols is quite difficult in the presence of a weak O–H bond. Consequently, transforming phenol into its derivatives, such as ethers, esters, sulfonates, carbamates, and metal salts, is necessary to remove the acidic proton; additionally, it is necessary to lower the BDE of C–O bonds to enable a smooth oxidative addition of a transition metal into it [9,10]. However, this process involves an additional derivatization step with an increase in the toxicity of the substrate, which is not aligned with the principles of green chemistry. Thus, there is an urgent need to develop a convenient method for the deoxygenative functionalization of unprotected phenols.

During the past two decades, organoboron compounds have gained considerable attention as one of the key substrates in Suzuki-Miyaura coupling [11,12,13] and other related coupling reactions [11]. Furthermore, arylboronic acids have been found to be suitable in mechanochemical Suzuki-Miyaura cross-coupling reactions [14,15,16,17,18]. Due to their remarkable stability, non-toxicity, high functional group tolerance, and commercial availability, organoboron compounds have become the most versatile substrates in synthetic chemistry to construct C–C, C–O, and C–O bonds in a single step [11,12,13,14,15,16,17,18,19]. Thus, significant efforts have been directed toward the synthesis of organoboron derivatives [20,21,22,23,24,25]. Among them, the Miyaura borylation of aryl halides or pseudohalides using transition metals represents an important tool for the construction of C–B bonds in a single step [21,22,23,26]. Miyaura borylation has also been performed under solvent-free mechanochemical conditions using Pd-catalyzed cross-coupling between aryl halides and bis(pinacolato)diboron [25]. Nevertheless, the generation of halogen-containing waste may limit the synthetic utility of this method in pharmaceutical industries. In this context, the utilization of unprotected (free) phenols in cross-coupling reactions has gained much attention, since phenol derivatives are cheap, readily available, non-toxic, and avoid the generation of halogen-containing waste.

**Scheme 1:**
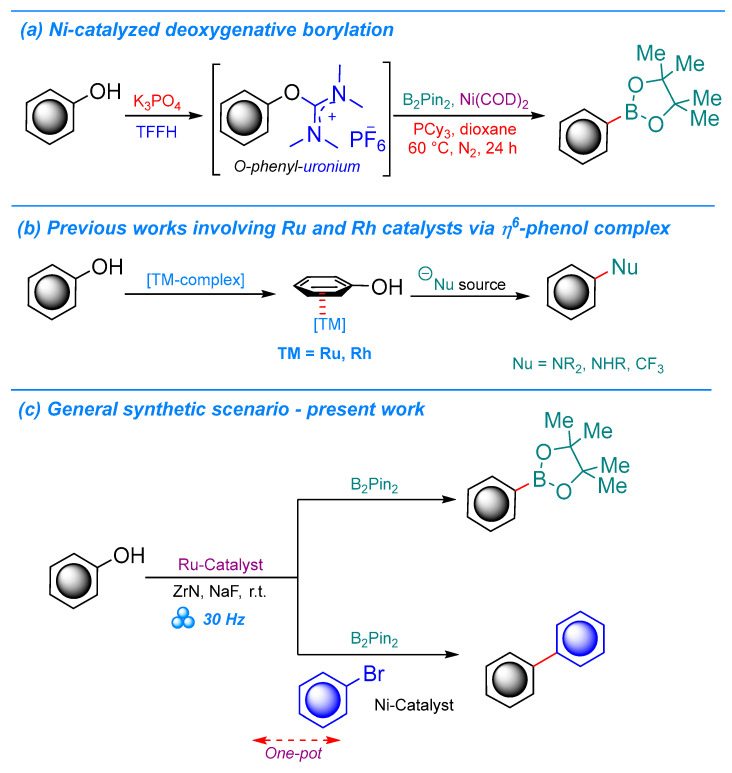
Reported strategies for the transition metal-catalyzed deoxygenative functionalization of phenols (**a**,**b**) and our approach (**c**).

In 2011, Yu and Shi described a mutual activation between naphtholates and aryl boronic acids via the formation of borates, which facilitated the Suzuki-Miyaura coupling under Ni-catalysis [27]. Later, in a seminal work, the same group showed an analogous approach for the direct borylation of β-naphthol [28]. In 2022, Liu et al. disclosed another strategy on deoxygenative borylation utilizing unprotected phenols through a combination of *tetramethylfluoroformamidinium hexafluorophosphate* (TFFH) and a Ni catalyst (Scheme 1a) [7]. Although this methodology is useful with a wide range of phenolic substrates, the requirement of a TFFH activator, *tricyclohexylphosphine* ligand, high temperature, inert atmosphere, and prolonged reaction times has significantly limited its practical utility. Until now, no methods were known to substitute the inert phenolic –OH groups with boronic esters without derivatization; however, attempts have been made to manipulate the aromatic –OH group with transition metal catalysis. For example, Shi and coworkers recently developed two methods for the catalytic amination of phenols with amines using Rh and Ru complexes (Scheme 1b) [29,30]. The mechanistic manipulation of these methodologies revealed the formation of metal-arene *π*-coordination complexes, i.e., *η*^6^-phenol complexes which facilitated the formation of *η*^5^-phenoxo complexes via inherently difficult keto–enol tautomerization, due to the electron-withdrawing effect of metals, and allowed the subsequent nucleophilic attack of amines to occur.

Based on our previous attempts [31,32,33,34,35,36,37,38,39,40,41,42,43,44,45] and Katayev’s work [46] on mechanochemical functionalizations, we envisioned that C–O bond activation could occur without the prior derivatization of phenol in the solid phase via *π*-coordination activation [47,48,49,50,51,52,53]. With this understanding and inspired by our recently developed deoxygenative trifluoromethylation approach [54], we set out to investigate the catalytic applicability of the [Cp*Ru(Napht)]BF_4_ complex for other cross-coupling reactions. The naphthalene ligand in the Ru-complex easily undergoes arene exchange by the substituted benzenes, including phenols [55]. We were curious to determine if mechanochemical friction would induce reactivity as expected, according to the scenario shown in Scheme 1c, for the proposed deoxygenative cross-coupling approach.

## 2. Results and Discussion

With this idea, a model reaction of *4-methylphenol* **1a** with 1.1 equiv. *bis(pinacolato)diboron* (B_2_Pin_2_) was performed in the presence of an appropriate Ru-catalyst (0.1 equiv.), KF (1.5 equiv.) as an activator (for B_2_Pin_2_), *1,4-diazabicyclo[2.2.2]octane* (DABCO) (1.2 equiv.) as a base, ZrN (4 equiv.) as an additive, and *1,4-dioxane* (0.2 mL) as a lubricant in a molecular mill with working frequency of 30 Hz for 60 min at room temperature (Table S1). Unfortunately, the reactions failed in the presence of the dimeric ruthenium complexes [Cp*RuCl_2_]_2_ and [(*p*-cymene)RuCl_2_]_2_ (Table S1, entry 1 and 2). Similar results were observed using a bis(*η*^6^-)arene Ru-complex, [(C_6_H_6_)_2_Ru](BF_4_)_2_ (Table S1, entry 3). To our delight, the formation of the expected aryl pinacolborane **2a** was observed with 42% yield in the presence of a labile *η*^5^-Ru-complex [Cp*Ru(MeCN)_3_]BF_4_ (Table S1, entry 4). This positive result prompted us further to screen other substitutionally labile sandwich-type Ru complexes, such as [Cp*Ru(PhCl)]PF_6_ and [Cp*Ru(Napht)]BF_4_ (Table S1, entry 5 and 6). The model reaction proceeded with almost quantitative yield (96%) of the respective aryl pinacolborane **2a** in the presence of [Cp*Ru(Napht)]BF_4_ (Table S1, entry 6). The importance of naphthalene ligand was confirmed, as [Cp*Ru(PhCl)]PF_6_ enabled the formation of product **2a** with 77% yield (Table S1, entry 5). Thus, [Cp*Ru(Napht)]BF_4_ was selected as the optimum choice as the catalyst in model reaction. The replacement of activator KF with NaF under the same conditions did not affect the yield of **2a** (Table S1, entry 7). Similar results were obtained upon decrement of the equivalents of NaF (from 1.5 to 1.2) (Table S1, entry 8). Furthermore, an experiment with a decrement (up to the half) in the equivalents of [Cp*Ru(Napht)]BF_4_ (0.05 equiv.) also led the formation of **2a** with 96% yield (Table S1, entry 9) which is the best outcome in the present optimization studies. A notable decrease in the yield of **2a** was observed with a further decrease in the equivalents of NaF (from 1.2 to 1.0) (Table S1, entry 10), while only a 40% yield of the same was observed in absence of any added activator (Table S1, entry 11). The presence of 1.2 equiv. of activator NaF and 0.05 equiv. of [Cp*Ru(Napht)]BF_4_ catalyst was sufficient to obtain Product **2a** in 96% yield, and therefore, entry 9 in Table S1 represents the optimal reaction conditions. In a separate experiment, the reaction with the equal ratio (1:1) of phenol **1a** and [Cp*Ru(Napht)]BF_4_ under optimized conditions yielded product **2a,** but with slightly diminished yield (73%) (Table S1, entry 12). The presence of other components, i.e., DABCO, ZrN and lubricant (*1,4-dioxane*), also played a significant role in the described transformation. To verify their specific role, three test reactions were performed in absence of each component (Table S1, entries 13–15). The reactions failed in the absence of DABCO and/or ZrN (Table S1, entry 13 and 15), while only 57% yield of product **2a** was detected in the absence of *1,4-dioxane* (Table S1, entry 14). From our previous studies on the deoxygenative trifluoromethylation of phenols [54], we observed that Lewis acid ZrN has potential to serve as an oxophilic additive to stabilize carbonyl functionalities, as well as having cationic vacancies (oxygen substitutions) which may induce ligand-to-metal charge transfer (LMCT). Thus, we directly opted for ZrN as an additive in the described transformation.

To validate the demand for mechanochemical conditions, the model reactions were also performed under conventional heating and stirring in a variety of organic media or under neat conditions (Table S1, entries 16–20). Nevertheless, all these solution phase reactions failed at higher as well as at lower temperatures, highlighting the importance of mechanochemistry over conventional methods. During mechanochemical ball milling, the mechanochemical forces (such as shear and compression) increased the surface area and reactivity, leading to more efficient transformations than under solution conditions.

**Scheme 2:**
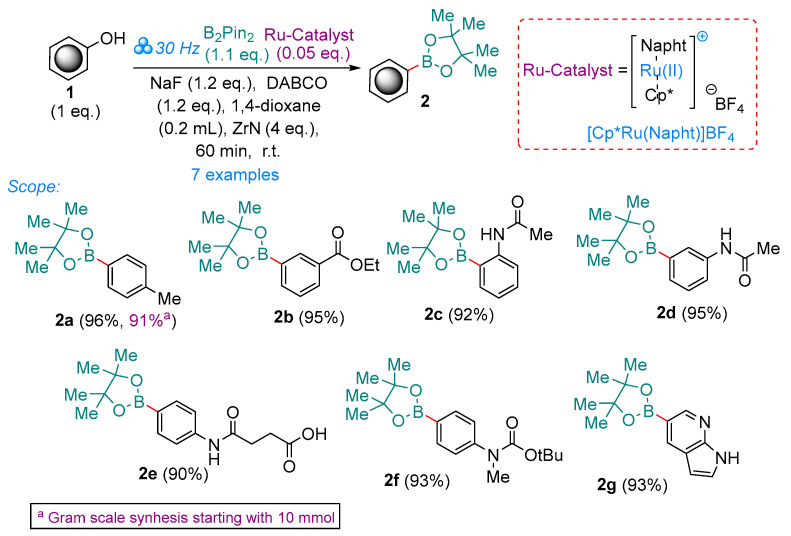
Substrate scope for the deoxygenative borylation of phenol **1**.

With the established optimized conditions, we explored the scope and generality of proposed deoxygenative borylation protocol, employing diversely substituted phenols (Scheme 2). Examining the scope, it was revealed that seven such phenolic substrates, including a fused heterocycle, were successfully transformed to the respective pinacolboranes using the above optimized conditions. Phenols with a wide range of electron donating or withdrawing groups, including nucleophile sensitive substituents, showed a considerable tolerance to produce the respective aryl pinacolboranes in excellent yields (90–96%). It is worth noting that with *4-aminophenol* and *p*-*tert*-*butylphenol*, the reactions failed. In the case of *4-aminophenol*, polycondensation might have taken place, while with *p*-*tert*-*butylphenol*, the bulky *tert*-butyl functionality might have destabilized the corresponding intermediates. Moreover, to validate the industrial utility of the proposed borylation approach, a synthesis at a 10 mmol scale was performed which led to the formation of compound **2a** with acceptable yields.

Inspired by the above success, we set out to extend the synthetic utility of the above established protocol for other synthetic scenarios, particularly for the deoxygenative cross-coupling of phenols with other coupling partners. On top of that, we decided to screen the same conditions with the additional presence of *bromobenzene* **3a** (1.2 equiv.) to establish the phenolic version of the well-known Suzuki-Miyaura cross-coupling reaction (between *phenol* and *bromobenzene*) via the in situ formation of aryl pinacolboranes (Table S2). Unfortunately, the test reaction failed to deliver the expected biphenyl product **4b** during a milling time of 90 min. (Table S2, entry 1). This failure might be attributed to the absence of palladium, which is mandatory in Suzuki-Miyaura cross-coupling. Thus, the same reaction was repeated in the presence of Pd-sources (0.1 equiv.) such as PdCl_2_(PPh_3_)_2_ and Pd(PPh_3_)_4_. To our delight, both reactions proceeded smoothly and delivered **4b** with 79% and 87% yields, respectively (Table S2, entry 2 and 3). The positive outcome of these experiments might have been due to the reaction of in situ generated aryl pinacolborane **2a** with *bromobenzene* **3a** in the presence of Pd-catalyst. Some recent studies have shown that nickel can also promote Suzuki-Miyaura cross-coupling reactions [56,57,58]. Thus, we decided to screen the model reaction using 0.1 equiv. of various Ni-complexes, as well as a combination of both a nickel salt and ligand (Table S2, entries 4–11). Moderate yields of product **4b** were detected in the presence of NiCl_2_(PPh_3_)_2_, NiBr_2_(PPh_3_)_2_, NiCl_2_(dppe), NiBr_2_(dppe), NiBr_2_/Xphos, and NiBr_2_/Xantphos (Table S2, entries 4–9). The best results were obtained with NiCl_2_(PCy_3_)_2_, leading to the formation of the expected product **4b** with 85% yield (Table S2, entry 11). In a separate experiment, we also screened a Ni-complex, (PPh_3_)_2_Ni(o-Tol)(Cl), which was previously reported to catalyze the cross-coupling reactions of aryl boronic acids and haloarenes [56]. The utilization of 0.05 equiv. (PPh_3_)_2_Ni(o-Tol)(Cl) in the model reaction delivered the desired product **4b** with 74% yield (Table S2, entry 10). This result prompted us to further decrease the equivalents of NiCl_2_(PCy_3_)_2_ in the model reactions.

Reducing the equivalents of NiCl_2_(PCy_3_)_2_ from 0.1 to 0.02 in the model reaction did not affect the yield of compound **4b** (Table S2, entries 11–15), while a further decrease of the same from 0.02 to 0.01 equiv. resulted a drastic decrease in the yield of **4b** (Table S2, entry 16). Thus, 0.02 equiv. of NiCl_2_(PCy_3_)_2_ was the optimum choice as a catalyst for the present cross-coupling reaction (Table S2, entry 15). Furthermore, the replacement of NiCl_2_(PCy_3_)_2_ with its bromo- analogue also gave a similar result (Table S2, entry 15); however, its cost effectiveness and availability made NiCl_2_(PCy_3_)_2_ the optimum choice as a catalyst.

**Scheme 3:**
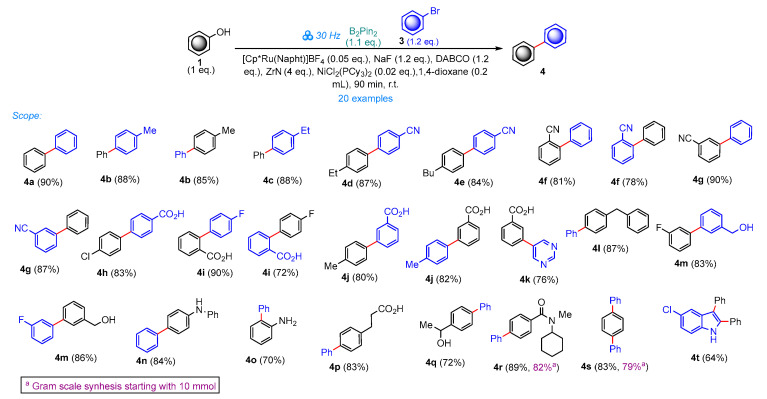
Substrate scope for the Suzuki-Miyaura type deoxygenative cross-coupling of phenol **1**.

With these optimized conditions, screening the scope and generality of the present Suzuki-Miyaura type deoxygenative cross-coupling protocol led to the synthesis of twenty biaryl analogues, **4a**–**t** (Scheme 3). The reactions of both phenol **1** and bromoarene **3** bearing electron-donating and/or withdrawing substituents proceeded smoothly to deliver the corresponding biaryl compounds **4** with excellent yields. Among them, six such biaryl analogues (**4b**, **4f**, **4g**, **4i**, **4j** and **4m**) were synthesized by switching the respective phenols and bromoarenes or vice versa. Furthermore, the present cross coupling approach was successfully extended for the synthesis of two double substitution products (**4s** and **4t**) and an arylpyrimidine derivative (**4k**) with acceptable yields. Furthermore, the proposed mechanochemical cross-coupling protocol not only showed a considerable tolerance toward a variety of phenols and bromoarenes substrates, but was also found to be feasible for the gram scale synthesis of two representatives, i.e., **4r** (82%), and **4s** (79%), with acceptable yields using the respective starting materials at a 10 mmol scale (Scheme 3).

**Scheme 4:**
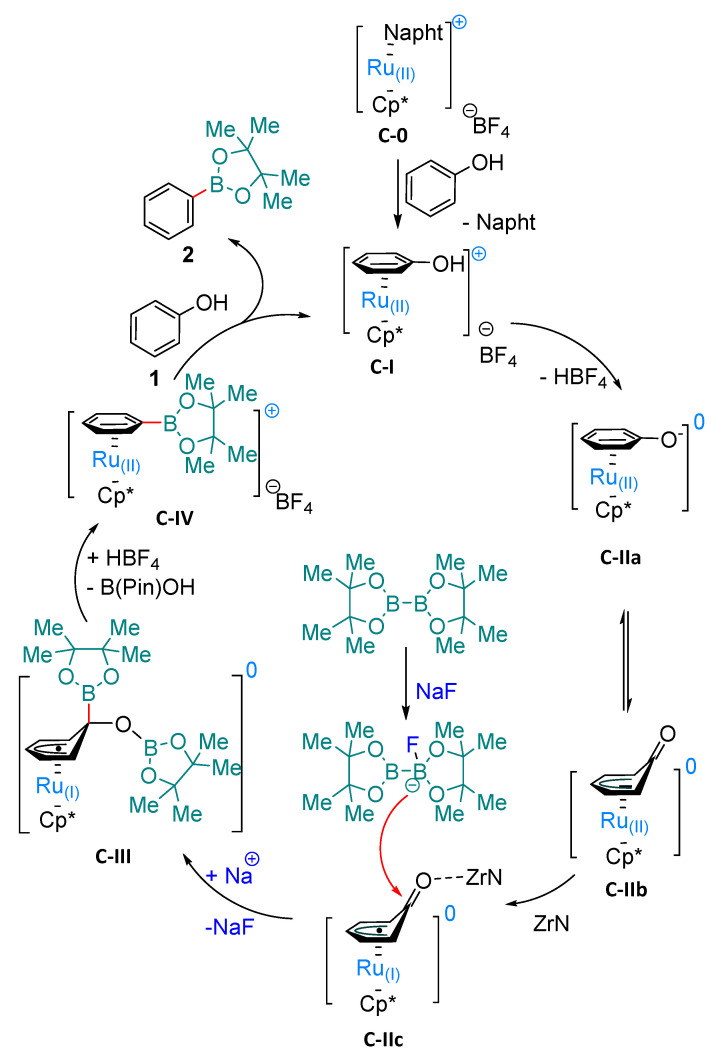
Plausible mechanism for the deoxygenative borylation of phenol **1**.

Following the available literature, the most plausible mechanism for the deoxygenative borylation of phenol is presented in Scheme 4. The initial arene exchange of the naphthalene by phenol **1** in the **C**-**0** complex to yield **C**-**I** is well established in the literature [54]. The deprotonation of the Ru-coordinated phenol in **C**-**I** (in presence of BF_4_^−^) leads to the formation of a neutral complex **C**-**IIa**, which exists in resonance with the *η*^5^-phenoxo complex **C**-**IIb**. Further, **C**-**IIb** is stabilized by the interaction with the solid adsorbent [59], in our case, the highly oxophilic ZrN [60] which, under mechanical conditions, induces LMCT to Ru(II), formally reducing it to Ru(I) to form intermediate **C**-**IIc***,* having a phenoxyl radical ligand. The radical character of species **C**-**IIc** provided a lower activation energy barrier (considering **C**-**IIc** against **C**-**IIb**) for the next step, i.e., for the nucleophilic attack of the anionic form of pinacolborane onto the carbonyl group, leading to the formation of **C**-**III**, which, in turn, liberated pinacolborane ester BPinOH upon interaction with HBF_4_. This protonation step induced the electron density to drop and return Ru(I) to the Ru(II) state. The resulting cationic *η*^6^-(trifluoromethyl)arene complex **C**-**IV** entered an aryl exchange step with phenolic starting material **1**, liberating product **2** and regenerating the active complex **C**-**I** for another turnover. To confirm the involvement of the radical species in the proposed mechanism, a radical trapping experiment was conducted by performing the model reaction with the additional presence (3 equiv.) of *2,2,6,6-tetramethyl-1-piperidinyloxy* (TEMPO). Expectedly, the reaction failed, confirming the proposed radical nature of the reaction.

**Figure 2:**
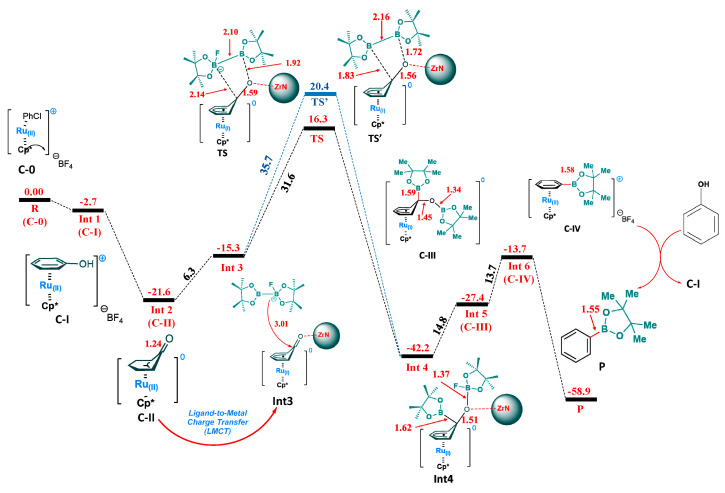
Free energy profile for the Ru-catalyzed deoxygenative borylation of unprotected phenols via C–O bond cleavage under mechanochemical conditions. Energy values are given in kcal/mol with respect to **C-0** complex at 0.00 kcal/mol. Bond distances are calculated in Angstroms (Å).

Density functional theory (DFT) simulations were performed to support the proposed mechanism of the Ru-catalyzed deoxygenative borylation of unprotected phenols via C–O bond cleavage under mechanochemical conditions (Figure 2). DFT study helped us to understand the kinetic barriers and thermodynamic stabilities of the various reaction steps involved in the mechanism. The mechanism of Ru-catalyzed activation started with the initial arene exchange of the naphthalene by phenol **1** in the **C**-**0** complex, which is well reported in the literature [54,55]. The ligand exchange resulted in the formation of a phenol-Ru-cyclopentadienyl sandwich complex designated as **C**-**I** (**Int1**). The **C**-**I** complex (**Int1**), with a free energy of −2.7 kcal/mol, revealed the stability of the **C**-**I** complex as compared to the **C-0** complex, i.e., the initial reactants. The reaction proceeded further with the deprotonation of the Ru-coordinated phenol in **C**-**I** (in the presence of BF_4_^-^), leading to the formation of neutral complex **C**-**II.** The **C**-**II** complex (**Int2**) was about 18.9 kcal/mol more stable than the initial reactant (**C**-**I** complex). At the **C**-**II** complex, the C–O bond distance decreased to 1.24 Å from 1.36 Å of **C**-**I** complex.

The complex **C**-**II** was further stabilized by the interaction with solid adsorbent ZrN [59,60]. This superconducting material, having cationic vacancies, induces, under mechanochemical conditions, ligand-to-metal charge transfer (LMCT) and generated **Int3** with phenoxyl radical ligand. The radical character of phenoxyl radicals significantly lowers the activation barrier for the nucleophilic attack of the anionic form pinacolborane on the *δ*^+^ carbon in the carbonyl groups. At **Int3**, anionic pinacolborane was introduced to the oxycyclohexadienyl-Ru-cyclopentadienyl complex with ZrN, which was energetically stabilized at −15.3 kcal/mol with respect to the **C**-**0** complex. At **Int3**, anionic pinacolborane interacted with the oxycyclohexadienyl-Ru-cyclopentadienyl complex, with an interaction distance of 3.01 Å. We performed natural bond orbital (NBO) analysis to validate the mode of charge transfer while moving from **Int2** to **Int3**. At **Int3**, the NBO charge calculated for ruthenium was −0.093e^−^ while at **Int2**, this charge was −0.015e^−^. The higher negative charge of **Int3** compared to that of Ru metal revealed that the charge was being transferred from the ligand to metal atom, which suggests that the mechanism followed ligand-to-metal charge transfer (LMCT).

This nucleophilic attack of anionic pinacolborane proceeded via a transition state **TS** with an activation barrier of 31.6 kcal/mol with respect to **Int3** and 16.3 kcal/mol with respect to complex **C**-**0**. For **TS**, the pinacolborane interacted with the *δ*^+^ carbon atoms of oxycyclohexadienyl rings at a distance of 2.14 Å, while the B–O interaction distance was 1.92 Å. Similarly, the B–B bond distance of pinacolborane increased to 2.10 Å from 1.74 Å at the **TS** (see Figure 2). Moreover, the C–O bond length also increased to 1.59 Å at the transition state. Similarly, we tried this step without the inclusion of fluorine anions. In that scenario, the new transition state **TS’** was located at a barrier height of 35.7 kcal/mol with respect to **Int3** and 20.4 kcal/mol with respect to **R**. The activation energy with and without fluorine anions revealed that the anionic form of pinacolborane attacked the *δ*^+^ carbon of the carbonyl group more effectively and was thermodynamically feasible due to lower energy barrier.

The nucleophilic attack of pinacolborane led to the formation of the **Int4** complex. The **Int4** complex was stable at 42.2 kcal/mol with reference to the initial reactants (**C**-**0** complex). The negative free stabilization energy revealed the thermodynamic stability of **Int4**. The removal of ZrN resulted in the formation of the **C**-**III** complex (**Int5**). **Int5** was stabilized at 27.4 kcal/mol with reference to the **C**-**0** complex. The **C**-**III** complex, in turn, liberated pinacolborane ester BPinOH upon interaction with HBF_4_, resulting in the formation of complex **C**-**IV** (**Int6**). The **C**-**IV** complex was located at 13.7 kcal/mol as compared to the **C-0** complex. The resulting cationic *η*^6^-(trifluoromethyl)arene complex **C**-**IV** entered an aryl exchange step with phenolic starting material **C**-**I**, liberating product **P** and reconstituting the active complex **C**-**I** for another turnover. The product **P** was located at −58.9 kcal/mol with respect to the initial reactant.

DFT studies revealed the feasibility of the proposed method for the Ru-catalyzed deoxygenative borylation of unprotected phenols via C–O bond cleavage under mechanochemical conditions. The thermodynamic and kinetic aspects of the proposed mechanism indicated that the Ru-catalyzed activation of deoxygenative borylation resulted in phenylboronic acid pinacol ester product **P (2)**, which is, thermodynamically, a feasible process due to the lower energy barrier of 31.6 kcal/mol under mechanochemical conditions.

## 3. Experimental Section

### 3.1. Materials and Methods

Commercially available starting materials, reagents, catalysts, anhydrous and degassed solvents were used without further purification. Flash column chromatography was performed with Merck Silica gel 60 (230–400 mesh). The solvents for column chromatography were distilled before use. Thin layer chromatography was carried out using Merck TLC Silica gel 60 F_254_ and visualized by short-wavelength ultraviolet light or by treatment with potassium permanganate (KMnO_4_) stain. ^1^H, ^13^C, and ^19^F NMR spectra were recorded on a Bruker 250, 400, and 500 MHz at 20 °C. All ^1^H NMR spectra are reported in parts per million (ppm) downfield of TMS and were measured relative to the signals for CHCl_3_ (7.26 ppm) and DMSO (2.50 ppm). All ^13^C{^1^H} NMR spectra are reported in ppm relative to residual CHCl_3_ (77.00 ppm) or DMSO (39.70 ppm) and were obtained with ^1^H decoupling. Coupling constants, *J*, are reported in Hertz (Hz). Mechanochemical synthesis was performed using the Retsch MM400 mill using the standard kit with a 5 mL grinding vessel (made of stainless steel) equipped with four balls (made of stainless steel, diameter: 5 mm). An A7820A gas chromatograph with quadrupole mass detector and flame ionization detector (Agilent, Santa Clara, CA, USA) were used. Melting points were measured using a Cole-Parmer^®^ MP-100 series Stuart analog melting point apparatus and are uncorrected. Liquid chemicals were dosed using gas-tight micro syringes. Isolation of obtained compounds was achieved by column chromatography on silica gel using *n*-hexane and ethyl acetate system as an eluent. All commercially available compounds were purchased from appropriate vendors.

### 3.2. Reaction Procedure with Optimised Reaction Conditions

#### 3.2.1. General Procedure for the Synthesis of Aryl Pinacolboranes **2** Starting from Phenol **1**

In a glovebox under a constant purge of argon, a 5 mL grinding vessel (made of stainless steel) equipped with two balls (made of stainless steel, diameter: 5 mm) was loaded consecutively with phenol starting material **1** (1 mmol, 1 equiv.), [Cp*Ru(Napht)]BF_4_ (22.6 mg, 0.05 mmol, 0.05 equiv.), bis(pinacolato)diboron (279 mg, 1.1 mmol, 1.1 equiv.), NaF (50 mg, 1.2 mmol, 1.2 equiv.), DABCO (135 mg, 1.2 mmol, 1.2 equiv.), and ZrN (421 mg, 4 mmol, 4 equiv.). Finaly, 1,4-dioxane (0.2 mL) was added. The reaction vessel was properly capped, installed on the mill, and subjected to milling at 30 Hz for 60 min at room temperature. The vessel was opened, and the content of the vessel was generously treated with distilled water, filtrated, and properly dried in a vacuum. The resulting crude product was directly subjected to gradient flash chromatography on silica gel using *n*-hexane and an ethyl acetate system as an eluent to isolate the desired product **2**.

#### 3.2.2. General Procedure for the Synthesis of Biphenyls **4** Starting from Phenol **1**

In a glovebox under a constant purge of argon, a 5 mL grinding vessel (made of stainless steel) equipped with three balls (made of stainless steel, diameter: 5 mm) was loaded consecutively with phenol starting material **1** (1 mmol, 1 equiv.), [Cp*Ru(Napht)]BF_4_ (22.6 mg, 0.05 mmol, 0.05 equiv.), bis(pinacolato)diboron (279 mg, 1.1 mmol, 1.1 equiv.), NaF (50 mg, 1.2 mmol, 1.2 equiv.), DABCO (135 mg, 1.2 mmol, 1.2 equiv.), appropriate aryl bromide **3** (1.2 mmol, 1.2 equiv.), NiCl_2_(PCy_3_)_2_ (14 mg, 0.02 mmol, 0.02 equiv.), and ZrN (421 mg, 4 mmol, 4 equiv.). Finaly, 1,4-dioxane (0.2 mL) was added. The reaction vessel was properly capped, installed on the mill, and subjected to milling at 30 Hz for 90 min at room temperature. The vessel was opened, and the contents were generously treated with distilled water, filtrated, and properly dried in vacuum. The resulting crude product was directly subjected to gradient flash chromatography on silica gel using *n*-hexane and an ethyl acetate system as an eluent to isolate the desired product **4**.

## 4. Conclusions

In conclusion, we have developed an efficient mechanochemical approach for the Ru-catalyzed deoxygenative borylation of unprotected phenols and demonstrated that this approach can also be extended to the Suzuki-Miyaura-type cross-coupling reaction of phenols with aryl bromides via in situ generated aryl pinacolboranes under the synergy of mechanochemical ball milling and Ru-Ni catalysis. The protocol was found to be simple and cost-effective for the selective cross-coupling of a variety of unprotected phenols under mechanochemical *π*-coordination-activation in the solid phase.

## Data Availability

Data are contained within the article and Supplementary Materials.

## Supplementary Materials

The following supporting information can be downloaded at: https://www.mdpi.com/article/10.3390/molecules30081835/s1, Scheme S1: List of phenols **1** used for the synthesis of aryl pinacolboranes **2**.; Scheme S2: List of phenols **1** used for the synthesis of biaryl compounds **4**.; Scheme S3: List of aryl bromides **3** used for the synthesis of biaryl compounds **4**.; Figure S1: Optimized geometries of reactants, intermediates, transitions state and final product along with important bond lengths.; Table S1: Optimization of the reaction conditions for the deoxygenative borylation of 4-methylphenol **1a**. Table S2: Optimization of the mechanochemical reaction conditions. References [61,62,63,64] are cited in the Supplementary Materials.
