# Supplementary material for: Mechanochemical Sequential Deoxygenative Cross-Coupling Reactions of Phenols Under Ruthenium-Nickel Catalysis
Source: Molecules. 2025 Apr 19;30(8):1835. doi: 10.3390/molecules30081835 (PMC12029765; doi:10.3390/molecules30081835)
Supplement: Supplementary file 1 [file molecules-30-01835-s001.zip › molecules-3594080-supplementary.pdf]

Supporting Information

Mechanochemical sequential deoxygenative cross-coupling reactions of phenols under Ruthenium-Nickel catalysis.

Table of Contents

(A) Experimental Section S3
(A-1) Scope of reagents used S3
(A-2) Reaction conditions screening S5
(B) Characterization of products S8
(C) Copies ^{1}H and ^{13}C NMR spectra S22
(D) Computational DFT studies Data S78

(A) Experimental Section.

Commercially available starting materials, reagents, catalysts, anhydrous and degassed solvents were used without further purification. Flash column chromatography was performed with Merck Silica gel 60 (230-400 mesh). The solvents for column chromatography were distilled before the use. Thin layer chromatography was carried out using Merck TLC Silica gel 60 F_{254} and visualized by short-wavelength ultraviolet light or by treatment with potassium permanganate ($KMnO_4$) stain. ^{1}H, ^{13}C and ^{19}F NMR spectra were recorded on a Bruker 250, 400 and 500 MHz at 20ºC. All ^{1}H NMR spectra are reported in parts per million (ppm) downfield of TMS and were measured relative to the signals for $CHCl_3$ (7.26 ppm) and DMSO (2.50 ppm). All ^{13}C{^{1}H} NMR spectra were reported in ppm relative to residual $CHCl_3$ (77.00 ppm) or DMSO (39.70 ppm) and were obtained with ^{1}H decoupling. Coupling constants, *J*, are reported in Hertz (Hz). Mechanochemical synthesis was performed using the Retsch MM400 mill using the standard kit with 10 mL grinding vessel (made of stainless steel) equipped with 4 balls (made of stainless steel, diameter: 5 mm). Gas chromatograph 7820A with quadrupole mass detector and flame ionization detector (Agilent, USA). Melting points were measured using Cole-Parmer® MP-100 series stuart analog melting point apparatus and are uncorrected. Liquid chemicals were dosed using gas tight micro syringes. Isolation of obtained compounds was achieved by column chromatography on Silica gel using n-hexane and ethyl acetate system as an eluent. All commercially available compounds were purchased from appropriate vendors.

A-1. Scope of reagents used.

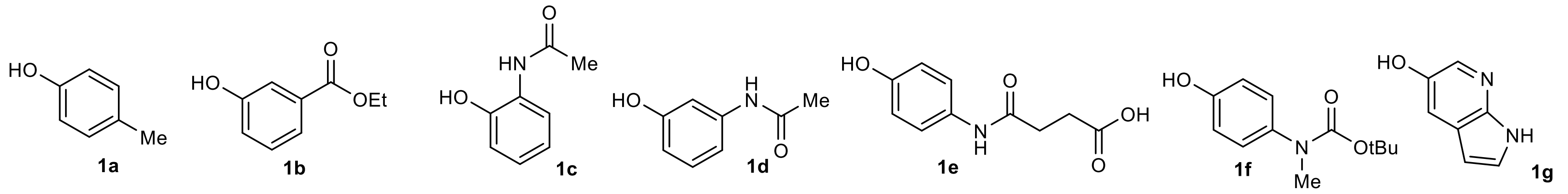

Scheme S1. List of phenols 1 used for the synthesis of aryl pinacolboranes 2.

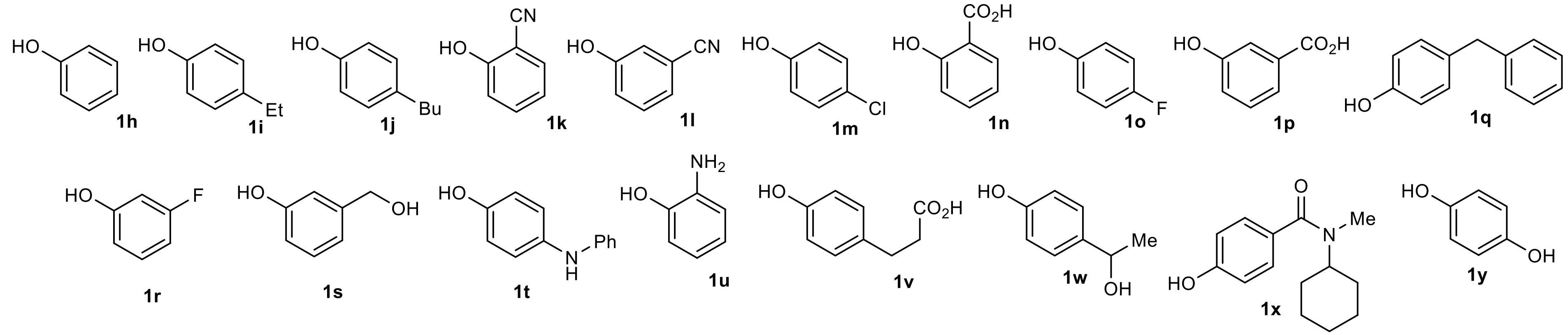

Scheme S2. List of phenols 1 used for the synthesis of biaryl compounds 4.

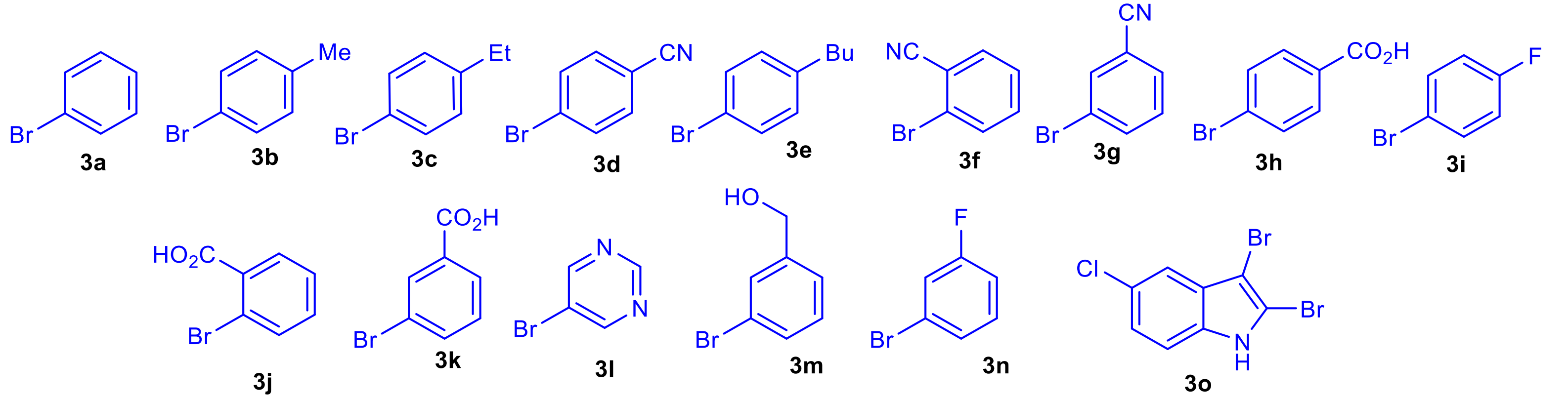

Scheme S3. List of aryl bromides 3 used for the synthesis of biaryl compounds 4.

A-2. Reaction conditions screening.

Table S1: Optimization of the reaction conditions for the deoxygenative borylation of 4-methylphenol **1a**.

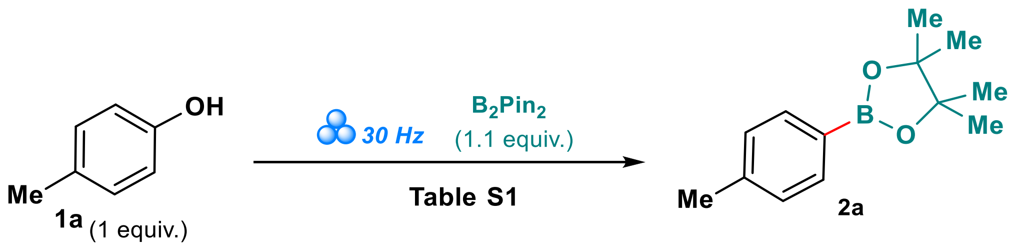

Table S1

Entry	Reaction components	Frequency/Time/ Temperature	Yield (%) 2a
**Reactions in solid phase**			
1	[Cp*$RuCl_2$]$_2$ (0.1 equiv.), KF (1.5 equiv.), DABCO (1.2 equiv.), 1,4-dioxane (0.2 mL), ZrN (4 equiv.).	30 Hz/60 min/r.t.	0
2	[(*p*-cymene)$RuCl_2$]$_2$ (0.1 equiv.), KF (1.5 equiv.), DABCO (1.2 equiv.), 1,4-dioxane (0.2 mL), ZrN (4 equiv.).	30 Hz/60 min/r.t.	0
3	[$(C_6H_6)_2Ru$]$(BF_4)_2$ (0.1 equiv.), KF (1.5 equiv.), DABCO (1.2 equiv.), 1,4-dioxane (0.2 mL), ZrN (4 equiv.).	30 Hz/60 min/r.t.	0
4	[Cp*Ru$(MeCN)_3$]BF_4 (0.1 equiv.), KF (1.5 equiv.), DABCO (1.2 equiv.), 1,4-dioxane (0.2 mL), ZrN (4 equiv.).	30 Hz/60 min/r.t.	42
5	[Cp*Ru(PhCl)]PF_6 (0.1 equiv.), KF (1.5 equiv.), DABCO (1.2 equiv.), 1,4-dioxane (0.2 mL), ZrN (4 equiv.).	30 Hz/60 min/r.t.	77
6	[Cp*Ru(Napht)]BF_4 (0.1 equiv.), KF (1.5 equiv.), DABCO (1.2 equiv.), 1,4-dioxane (0.2 mL), ZrN (4 equiv.).	30 Hz/60 min/r.t.	96
7	[Cp*Ru(Napht)]BF_4 (0.1 equiv.), NaF (1.5 equiv.), DABCO (1.2 equiv.), 1,4-dioxane (0.2 mL), ZrN (4 equiv.).	30 Hz/60 min/r.t.	96
8	[Cp*Ru(Napht)]BF_4 (0.1 equiv.), NaF (1.2 equiv.), DABCO (1.2 equiv.), 1,4-dioxane (0.2 mL), ZrN (4 equiv.).	30 Hz/60 min/r.t.	96
**9**	**[Cp*Ru(Napht)]BF_4 (0.05 equiv.), NaF (1.2 equiv.), DABCO (1.2 equiv.), 1,4-dioxane (0.2 mL), ZrN (4 equiv.).**	**30 Hz/60 min/r.t.**	96
10	[Cp*Ru(Napht)]BF_4 (0.05 equiv.), NaF (1.0 equiv.), DABCO (1.2 equiv.), 1,4-dioxane (0.2 mL), ZrN (4 equiv.).	30 Hz/60 min/r.t.	88
11	[Cp*Ru(Napht)]BF_4 (0.05 equiv.), DABCO (1.2 equiv.), 1,4-dioxane (0.2 mL), ZrN (4 equiv.).	30 Hz/60 min/r.t.	40
12	[Cp*Ru(Napht)]BF_4 (1.0 equiv.), NaF (1.2 equiv.), DABCO (1.2 equiv.), 1,4-dioxane (0.2 mL), ZrN (4 equiv.).	30 Hz/60 min/r.t.	73
13	[Cp*Ru(Napht)]BF_4 (0.05 equiv.), NaF (1.0 equiv.), 1,4-dioxane (0.2 mL), ZrN (4 equiv.).	30 Hz/60 min/r.t.	0
14	[Cp*Ru(Napht)]BF_4 (0.05 equiv.), NaF (1.0 equiv.), DABCO (1.2 equiv.), ZrN (4 equiv.).	30 Hz/60 min/r.t.	57
15	[Cp*Ru(Napht)]BF_4 (0.05 equiv.), NaF (1.0 equiv.), DABCO (1.2 equiv.), 1,4-dioxane (0.2 mL).	30 Hz/60 min/r.t.	0
**Reactions in solution**			
16	[Cp*Ru(Napht)]BF_4 (0.05 equiv.), NaF (1.2 equiv.), DABCO (1.2 equiv.), ZrN (4 equiv.), toluene, reflux.	------/24 h	0
17	[Cp*Ru(Napht)]BF_4 (0.05 equiv.), NaF (1.2 equiv.), DABCO (1.2 equiv.), ZrN (4 equiv.), benzene, reflux.	------/24 h	0
18	[Cp*Ru(Napht)]BF_4 (0.05 equiv.), NaF (1.2 equiv.), DABCO (1.2 equiv.), ZrN (4 equiv.), 1,4-dioxan, reflux.	------/24h	0
19	[Cp*Ru(Napht)]BF_4 (0.05 equiv.), NaF (1.2 equiv.), DABCO (1.2 equiv.), ZrN (4 equiv.), xylenes, 130 ^oC.	------/24h	0
20	[Cp*Ru(Napht)]BF_4 (0.05 equiv.), NaF (1.2 equiv.), DABCO (1.2 equiv.), ZrN (4 equiv.), neat, 170 ^oC.	------/24h	0

Table S2: Optimization of the mechanochemical reaction conditions.

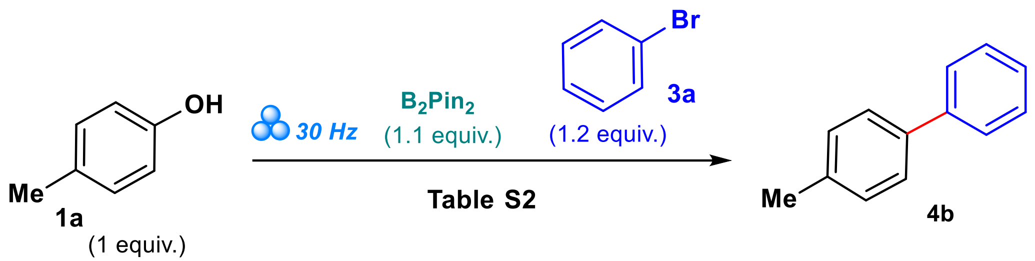

Table S2			
**Entry**	**Reaction components**	**Frequency/Time/ Temperature**	**Yield (%) 4a**
1	[Cp*Ru(Napht)]BF_4 (0.05 equiv.), NaF (1.2 equiv.), DABCO (1.2 equiv.), 1,4-dioxane (0.2 mL), ZrN (4 equiv.).	30 Hz/90 min/r.t.	0
2	[Cp*Ru(Napht)]BF_4 (0.05 equiv.), NaF (1.2 equiv.), DABCO (1.2 equiv.), 1,4-dioxane (0.2 mL), ZrN (4 equiv.), $PdCl_2(PPh_3)_2$ (0.1 equiv.).	30 Hz/90 min/r.t.	79
3	[Cp*Ru(Napht)]BF_4 (0.05 equiv.), NaF (1.2 equiv.), DABCO (1.2 equiv.), 1,4-dioxane (0.2 mL), ZrN (4 equiv.), $Pd(PPh_3)_4$ (0.1 equiv.).	30 Hz/90 min/r.t.	87
4	[Cp*Ru(Napht)]BF_4 (0.05 equiv.), NaF (1.2 equiv.), DABCO (1.2 equiv.), 1,4-dioxane (0.2 mL), ZrN (4 equiv.), $NiCl_2(PPh_3)_2$ (0.1 equiv.).	30 Hz/90 min/r.t.	31
5	[Cp*Ru(Napht)]BF_4 (0.05 equiv.), NaF (1.2 equiv.), DABCO (1.2 equiv.), 1,4-dioxane (0.2 mL), ZrN (4 equiv.), $NiBr_2(PPh_3)_2$ (0.1 equiv.).	30 Hz/90 min/r.t.	44
6	[Cp*Ru(Napht)]BF_4 (0.05 equiv.), NaF (1.2 equiv.), DABCO (1.2 equiv.), 1,4-dioxane (0.2 mL), ZrN (4 equiv.), $NiCl_2$(dppe) (0.1 equiv.).	30 Hz/90 min/r.t.	42
7	[Cp*Ru(Napht)]BF_4 (0.05 equiv.), NaF (1.2 equiv.), DABCO (1.2 equiv.), 1,4-dioxane (0.2 mL), ZrN (4 equiv.), $NiBr_2$(dppe) (0.1 equiv.).	30 Hz/90 min/r.t.	48
8	[Cp*Ru(Napht)]BF_4 (0.05 equiv.), NaF (1.2 equiv.), DABCO (1.2 equiv.), 1,4-dioxane (0.2 mL), ZrN (4 equiv.), $NiBr_2$ (0.1 equiv.), Xphos (0.1 equiv.).	30 Hz/90 min/r.t.	62
9	[Cp*Ru(Napht)]BF_4 (0.05 equiv.), NaF (1.2 equiv.), DABCO (1.2 equiv.), 1,4-dioxane (0.2 mL), ZrN (4 equiv.), $NiBr_2$ (0.1 equiv.), Xantphos (0.1 equiv.).	30 Hz/90 min/r.t.	40

10	[Cp*Ru(Napht)]BF_4 (0.05 equiv.), NaF (1.2 equiv.), DABCO (1.2 equiv.), 1,4-dioxane (0.2 mL), ZrN (4 equiv.), $(PPh_3)_2$Ni(o-Tol)(Cl) (0.05 equiv.).	30 Hz/90 min/r.t.	74
11	[Cp*Ru(Napht)]BF_4 (0.05 equiv.), NaF (1.2 equiv.), DABCO (1.2 equiv.), 1,4-dioxane (0.2 mL), ZrN (4 equiv.), $NiCl_2(PCy_3)_2$ (0.1 equiv.).	30 Hz/90 min/r.t.	85
12	[Cp*Ru(Napht)]BF_4 (0.05 equiv.), NaF (1.2 equiv.), DABCO (1.2 equiv.), 1,4-dioxane (0.2 mL), ZrN (4 equiv.), $NiCl_2(PCy_3)_2$ (0.05 equiv.).	30 Hz/90 min/r.t.	85
13	[Cp*Ru(Napht)]BF_4 (0.05 equiv.), NaF (1.2 equiv.), DABCO (1.2 equiv.), 1,4-dioxane (0.2 mL), ZrN (4 equiv.), $NiCl_2(PCy_3)_2$ (0.04 equiv.).	30 Hz/90 min/r.t.	85
14	[Cp*Ru(Napht)]BF_4 (0.05 equiv.), NaF (1.2 equiv.), DABCO (1.2 equiv.), 1,4-dioxane (0.2 mL), ZrN (4 equiv.), $NiCl_2(PCy_3)_2$ (0.03 equiv.).	30 Hz/90 min/r.t.	85
**15**	**[Cp*Ru(Napht)]BF_4 (0.05 equiv.), NaF (1.2 equiv.), DABCO (1.2 equiv.), 1,4-dioxane (0.2 mL), ZrN (4 equiv.), $NiCl_2(PCy_3)_2$ (0.02 equiv.).**	**30 Hz/90 min/r.t.**	**85**
16	[Cp*Ru(Napht)]BF_4 (0.05 equiv.), NaF (1.2 equiv.), DABCO (1.2 equiv.), 1,4-dioxane (0.2 mL), ZrN (4 equiv.), $NiCl_2(PCy_3)_2$ (0.01 equiv.).	30 Hz/90 min/r.t.	61
17	[Cp*Ru(Napht)]BF_4 (0.05 equiv.), NaF (1.2 equiv.), DABCO (1.2 equiv.), 1,4-dioxane (0.2 mL), ZrN (4 equiv.), $NiBr_2(PCy_3)_2$ (0.02 equiv.).	30 Hz/90 min/r.t.	85

Reaction procedure with optimised reaction conditions.

General procedure for the synthesis of aryl pinacolboranes 2 starting from phenols 1:

In a glovebox under the constant purge of argon, 5 mL grinding vessel (made of stainless steel) equipped with two balls (made of stainless steel, diameter: 5 mm) was loaded consecutively with the phenol starting material **1** (1 mmol, 1 equiv.), [Cp*Ru(Napht)]BF_4 (22.6 mg, 0.05 mmol, 0.05 equiv.), bis(pinacolato)diboron (279 mg, 1.1 mmol, 1.1 equiv.), NaF (50 mg, 1.2 mmol, 1.2 equiv.), DABCO (135 mg, 1.2 mmol, 1.2 equiv.) and ZrN (421 mg, 4 mmol, 4 equiv.). Finaly, 1,4-dioxane (0.2 mL) was added. The reaction vessel was properly capped, installed on the mill and subjected to milling at 30 Hz for 60 minutes at room temperature. The vessel was opened and the content of the vessel was generously treated with distilled water, filtrated and properly dried in vacuum. The resulting crude product was directly subjected to gradient flash chromatography on silica gel using n-hexane and ethyl acetate system as an eluent to isolate the desired product **2**.

General procedure for the synthesis of biphenyls 4 starting from phenols 1:

In a glovebox under the constant purge of argon, 5 mL grinding vessel (made of stainless steel) equipped with three balls (made of stainless steel, diameter: 5 mm) was loaded consecutively with the phenol starting material **1** (1 mmol, 1 equiv.), [Cp*Ru(Napht)]BF_4 (22.6 mg, 0.05 mmol, 0.05 equiv.), bis(pinacolato)diboron (279 mg, 1.1 mmol, 1.1 equiv.), NaF (50 mg, 1.2 mmol, 1.2 equiv.), DABCO (135 mg, 1.2 mmol, 1.2 equiv.), appropriate aryl bromide **3** (1.2 mmol, 1.2 equiv.), $NiCl_2(PCy_3)_2$ (14 mg, 0.02 mmol, 0.02 equiv.) and ZrN (421 mg, 4 mmol, 4 equiv.). Finaly, 1,4-dioxane (0.2 mL) was added. The reaction vessel was properly capped, installed on the mill and subjected to milling at 30 Hz for 90 minutes at room temperature. The vessel was opened and the content of the vessel was generously treated with distilled water, filtrated and properly dried in vacuum. The resulting crude product was directly subjected to gradient flash chromatography on silica gel using n-hexane and ethyl acetate system as an eluent to isolate the desired product **4**.

(B) Characterization of products.

4,4,5,5-tetramethyl-2-(p-tolyl)-1,3,2-dioxaborolane **(2a)**

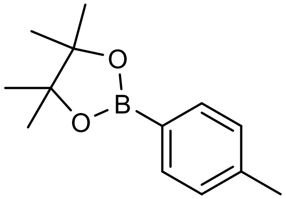

The title compound was prepared starting from phenol phenol **1a** (108 mg, 1 mmol, 1 equiv.), Cp*Ru(Napht)BF_4 (22.6 mg, 0.05 mmol, 0.05 equiv.), bis(pinacolato)diboron (279 mg, 1.1 mmol, 1.1 equiv.), NaF (50 mg, 1.2 mmol, 1.2 equiv.), DABCO (135 mg, 1.2 mmol, 1.2 equiv.), ZrN (421 mg, 4 mmol, 4 equiv.) and 1,4-dioxane (0.2 mL). The purification was accomplished by flash chromatography on silica gel to provide the desired product **2a** (209 mg, 0.96 mmol, 96%).

White solid, mp 53-54 °C. **^{1}H NMR (400 MHz, $CDCl_3$):** δ 1.36 (s, 12H, CH_3), 2.39 (s, 3H, CH_3),7.21 (d, 2H, ^3J = 7.6 Hz, CH_{Ar}), 7.74 (d, 2H, ^3J = 7.8 Hz, CH_{Ar}).

^{13}C NMR (100 MHz, $CDCl_3$): δ 21.8, 24.9, 83.6, 125.6, 128.6, 134.9, 141.4.

MS (GC, 70eV): m/z (%) = 218 (M^+, 37), 203 (47), 132 (66), 119 (100).

Anal. calcd. for $C_{13}H_{19}BO_2$: C, 71.59; H, 8.78. Found: C, 71.69; H, 8.71.

ethyl 3-(4,4,5,5-tetramethyl-1,3,2-dioxaborolan-2-yl)benzoate **(2b)**

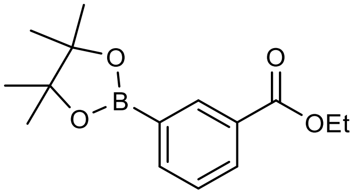

The title compound was prepared starting from phenol phenol **1b** (166 mg, 1 mmol, 1 equiv.), Cp*Ru(Napht)BF_4 (22.6 mg, 0.05 mmol, 0.05 equiv.), bis(pinacolato)diboron (279 mg, 1.1 mmol, 1.1 equiv.), NaF (50 mg, 1.2 mmol, 1.2 equiv.), DABCO (135 mg, 1.2 mmol, 1.2 equiv.), ZrN (421 mg, 4 mmol, 4 equiv.) and 1,4-dioxane (0.2 mL). The purification was accomplished by flash chromatography on silica gel to provide the desired product **2b** (262 mg, 0.95 mmol, 95%).

White solid, mp 40-42 °C. **^{1}H NMR (400 MHz, $CDCl_3$):** δ 1.34 (s, 12H, CH_3), 1.39 (t, 3H, ^3J = 7.2 Hz, CH_3) 4.37 (q, 2H, ^3J = 7.2 Hz, CH_2), 7.43 (t, 1H, ^3J = 7.6 Hz, CH_{Ar}), 7.97 (d, 1H, ^3J = 7.4 Hz, CH_{Ar}). 8.13 (dt, 1H, ^3J = 7.8 Hz, ^4J = 1.5 Hz, CH_{Ar}), 8.46 (s, 1H, CH_{Ar}).

^{13}C NMR (100 MHz, $CDCl_3$): δ 14.4, 24.9, 60.9, 84.1, 127.7, 129.9, 132.3, 135.7, 139.1, 166.7.

MS (GC, 70eV): m/z (%) = 276 (M^+, 21), 261 (36), 238 (100), 205 (75), 177 (82).

Anal. calcd. for $C_{15}H_{21}BO_4$: C, 65.24; H, 7.67. Found: C, 65.33; H, 7.71.

N-(2-(4,4,5,5-tetramethyl-1,3,2-dioxaborolan-2-yl)phenyl)acetamide **(2c)**

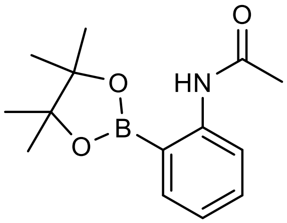

The title compound was prepared starting from phenol phenol **1c** (151 mg, 1 mmol, 1 equiv.), Cp*Ru(Napht)BF_4 (22.6 mg, 0.05 mmol, 0.05 equiv.), bis(pinacolato)diboron (279 mg, 1.1 mmol, 1.1 equiv.), NaF (50 mg, 1.2 mmol, 1.2 equiv.), DABCO (135 mg, 1.2 mmol, 1.2 equiv.), ZrN (421 mg, 4 mmol, 4 equiv.) and 1,4-dioxane (0.2 mL). The purification was accomplished by flash chromatography on silica gel to provide the desired product **2c** (240 mg, 0.92 mmol, 92%).

Beige solid, mp 173-174 °C. **^{1}H NMR (400 MHz, DMSO-*d₆*):** δ 1.15 (s, 12H, CH_3), 2.25 (s, 3H, CH_3), 6.98 (d, 1H, ^3J = 7.8 Hz, CH_{Ar}), 7.11 (d, 1H, ^3J = 7.3 Hz, CH_{Ar}), 7.22 (td, 1H, ^3J = 7.7 Hz, ^4J = 1.6 Hz, CH_{Ar}), 7.40 (d, 1H, ^3J = 7.3 Hz, CH_{Ar}), 11.79 (s, 1H, NH).

^{13}C NMR (100 MHz, DMSO-d_6): δ 22.3, 26.5, 80.0, 116.0, 125.7, 127.9, 133.1, 138.6, 170.3.

MS (GC, 70eV): m/z (%) = 261 (M^+, 36), 246 (15), 202 (100).
Anal. calcd. for $C_{14}H_{20}BNO_3$: C, 64.40; H, 7.72; N; 5.36. Found: C, 64.31; H, 7.79, N; 5.28.

N-(3-(4,4,5,5-tetramethyl-1,3,2-dioxaborolan-2-yl)phenyl)acetamide **(2d)**

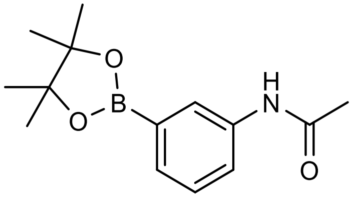

The title compound was prepared starting from phenol phenol **1d** (151 mg, 1 mmol, 1 equiv.), Cp*Ru(Napht)BF_4 (19 mg, 0.05 mmol, 0.05 equiv.), bis(pinacolato)diboron (279 mg, 1.1 mmol, 1.1 equiv.), NaF (50 mg, 1.2 mmol, 1.2 equiv.), DABCO (135 mg, 1.2 mmol, 1.2 equiv.), ZrN (421 mg, 4 mmol, 4 equiv.) and 1,4-dioxane (0.2 mL). The purification was accomplished by flash chromatography on silica gel to provide the desired product **2d** (248 mg, 0.95 mmol, 95%).
White solid, mp 186-187 °C. **^{1}H NMR (400 MHz, $CDCl_3$):** δ 1.30 (s, 12H, CH_3), 2.14 (s, 3H, CH_3), 7.30 (t, 1H, ^3J = 7.6 Hz, CH_{Ar}), 7.52 (d, 1H, ^3J = 7.2 Hz, CH_{Ar}), 7.73 (s, 1H, CH_{Ar}), 7.81 (d, 1H, ^3J = 7.8, CH_{Ar}), 7.92 (s, 1H, NH).
^{13}C NMR (100 MHz, $CDCl_3$): δ 24.5, 24.9, 83.9, 123.2, 126.1, 128.5, 130.5, 137.6, 168.8.
MS (GC, 70eV): m/z (%) = 261 (M^+, 78), 246 (10), 218 (100), 200 (19), 165 (35) 133 (27), 119 (67).
Anal. calcd. for $C_{14}H_{20}BNO_3$: C, 64.40; H, 7.72; N; 5.36. Found: C, 64.43; H, 7.76, N; 5.42.

4-oxo-4-((4-(4,4,5,5-tetramethyl-1,3,2-dioxaborolan-2-yl)phenyl)amino)butanoic acid **(2e)**

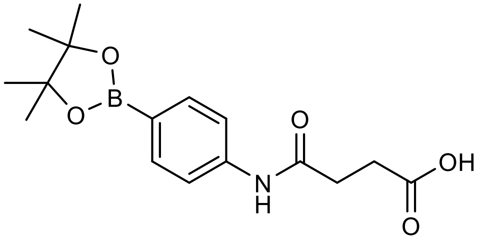

The title compound was prepared starting from phenol phenol **1e** (209 mg, 1 mmol, 1 equiv.), Cp*Ru(Napht)BF_4 (22.6 mg, 0.05 mmol, 0.05 equiv.), bis(pinacolato)diboron (279 mg, 1.1 mmol, 1.1 equiv.), NaF (50 mg, 1.2 mmol, 1.2 equiv.), DABCO (135 mg, 1.2 mmol, 1.2 equiv.), ZrN (421 mg, 4 mmol, 4 equiv.) and 1,4-dioxane (0.2 mL). The purification was accomplished by flash chromatography on silica gel to provide the desired product **2e** (287 mg, 0.90 mmol, 90%).
White solid, mp 176-177 °C. **^{1}H NMR (400 MHz, DMSO-d_6):** δ 1.25 (s, 12H, CH_3), 2.52 (d, 2H, ^3J = 5.7 Hz, CH_{Ar}), 2.56 (d, 2H, ^3J = 5.7 Hz, CH_{Ar}), 7.59 (m, 4H, CH_{Ar}), 10.05 (s, 1H, NH), 12.12 (br, 1H, COOH).

^{13}C NMR (100 MHz, DMSO-d_6): δ 25.1, 29.2, 31.6, 83.9, 118.4, 123.0, 135.7, 142.6, 170.9, 174.3.
Anal. calcd. for $C_{16}H_{22}BNO_5$: C, 60.21; H, 6.95; N; 4.39. Found: C, 60.32; H, 7.04, N; 4.31.

***tert-butyl methyl(4-(4,4,5,5-tetramethyl-1,3,2-dioxaborolan-2-yl)phenyl)carbamate* (2f)**

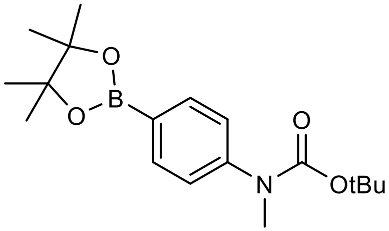

The title compound was prepared starting from phenol phenol **1f** (223 mg, 1 mmol, 1 equiv.), Cp*Ru(Napht)BF_4 (22.6 mg, 0.05 mmol, 0.05 equiv.), bis(pinacolato)diboron (279 mg, 1.1 mmol, 1.1 equiv.), NaF (50 mg, 1.2 mmol, 1.2 equiv.), DABCO (135 mg, 1.2 mmol, 1.2 equiv.), ZrN (421 mg, 4 mmol, 4 equiv.) and 1,4-dioxane (0.2 mL). The purification was accomplished by flash chromatography on silica gel to provide the desired product **2f** (310 mg, 0.93 mmol, 93%).
White solid, mp 108-109 ^{o}C. **^{1}H NMR (400 MHz, CDCl$_3$):** δ 1.29 (s, 12H, CH_3), 1.41 (s, 9H, CH_3) 3.22 (s, 3H, CH_3), 7.21 (d, 2H, ^{3}J = 8.4 Hz, CH_{Ar}), 7.73 (d, 2H, ^{3}J = 8.2 Hz, CH_{Ar}).
^{13}C NMR (100 MHz, CDCl$_3$): δ 24.9, 28.3, 37.0, 80.3, 83.7, 124.3, 135.1, 146.5, 154.4.
MS (GC, 70eV): m/z (%) = 333 (M^+, 3), 277 (44), 233 (100), 218 (11), 160 (23).
Anal. calcd. for $C_{18}H_{28}BNO_4$: C, 64.88; H, 8.47; N; 4.20. Found: C, 64.91; H, 8.31, N; 4.10.

***5-(4,4,5,5-tetramethyl-1,3,2-dioxaborolan-2-yl)-1H-pyrrolo[2,3-b]pyridine* (2g)**

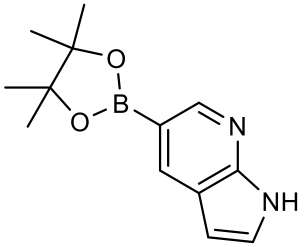

The title compound was prepared starting from phenol phenol **1g** (134 mg, 1 mmol, 1 equiv.), Cp*Ru(Napht)BF_4 (22.6 mg, 0.05 mmol, 0.05 equiv.), bis(pinacolato)diboron (279 mg, 1.1 mmol, 1.1 equiv.), NaF (50 mg, 1.2 mmol, 1.2 equiv.), DABCO (135 mg, 1.2 mmol, 1.2 equiv.), ZrN (421 mg, 4 mmol, 4 equiv.) and 1,4-dioxane (0.2 mL). The purification was accomplished by flash chromatography on silica gel to provide the desired product **2g** (227 mg, 0.93 mmol, 93%).

White solid, mp 242-243 °C. **^{1}H NMR (400 MHz, $CDCl_3$):** δ 1.39 (s, 12H, CH_3), 6.53 (d, 1H, ^4J = 3.2 Hz, CH_{Ar}), 7.40 (d, 1H, ^4J = 3.1 Hz, CH_{Ar}), 8.44 (s, 1H, CH_{Ar}), 8.77 (s, 1H, CH_{Ar}), 12.44 (s, 1H, NH).
^{13}C NMR (100 MHz, $CDCl_3$): δ 24.9, 83.8, 101.0, 114.8, 120.3, 125.5, 136.2, 148.4, 150.5.
MS (GC, 70eV): m/z (%) = 244 (M^+, 76), 229 (23), 158 (40), 144 (100).
Anal. calcd. for $C_{13}H_{17}BN_2O_2$: C, 63.97; H, 7.02; N; 11.48. Found: C, 64.07; H, 7.11, N; 11.52.

1,1'-biphenyl **(4a)**

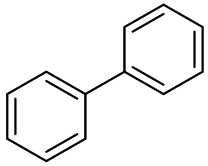

The title compound was prepared starting from phenol **1h** (94 mg, 1 mmol, 1 equiv.), Cp*Ru(Napht)BF_4 (22.6 mg, 0.05 mmol, 0.05 equiv.), bis(pinacolato)diboron (279 mg, 1.1 mmol, 1.1 equiv.), NaF (50 mg, 1.2 mmol, 1.2 equiv.), DABCO (135 mg, 1.2 mmol, 1.2 equiv.), aryl bromide **3a** (188 mg, 1.2 mmol, 1.2 equiv.), $NiCl_2(PCy_3)_2$ (14 mg, 0.02 mmol, 0.02 equiv.), ZrN (421 mg, 4 mmol, 4 equiv.). and 1,4-dioxane (0.2 mL). The purification was accomplished by flash chromatography on silica gel to provide the desired product **4a** (227 mg, 0.90 mmol, 90%).
White solid, mp 68-69 °C. **^{1}H NMR (400 MHz, DMSO-d_6):** δ 7.32 (t, 2H, ^3J = 7.1 Hz, CH_{Ar}), 7.42 (t, 4H, ^3J = 7.6 Hz, CH_{Ar}), 7.61 (d, 4H, ^3J = 7.6 Hz, CH_{Ar}).
^{13}C NMR (100 MHz, DMSO-d_6): δ 127.2, 127.9, 129.4, 140.7.
MS (GC, 70eV): m/z (%) = 154 (M^+, 100), 76 (18).
Anal. calcd. for $C_{12}H_{10}$: C, 93.46; H, 6.54. Found: C, 93.57; H, 6.43.

4-methyl-1,1'-biphenyl **(4b)**

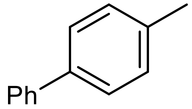

The title compound was prepared starting from phenol **1h** (94 mg, 1 mmol, 1 equiv.), Cp*Ru(Napht)BF_4 (22.6 mg, 0.05 mmol, 0.05 equiv.), bis(pinacolato)diboron (279 mg, 1.1 mmol, 1.1 equiv.), NaF (50 mg, 1.2 mmol, 1.2 equiv.), DABCO (135 mg, 1.2 mmol, 1.2 equiv.), aryl bromide **3b** (205 mg, 1.2 mmol, 1.2 equiv.), $NiCl_2(PCy_3)_2$ (14 mg, 0.02 mmol, 0.02 equiv.), ZrN (421 mg, 4 mmol, 4 equiv.). and 1,4-dioxane (0.2 mL). The purification was accomplished by flash chromatography on silica gel to provide the desired product **4b** (148 mg, 0.88 mmol, 88%).
Alternatively, the title compound was prepared starting from phenol **1a** (108 mg, 1 mmol, 1 equiv.), Cp*Ru(Napht)BF_4 (22.6 mg, 0.05 mmol, 0.05 equiv.), bis(pinacolato)diboron (279 mg, 1.1 mmol, 1.1 equiv.), NaF (50 mg, 1.2 mmol, 1.2 equiv.), DABCO (135 mg, 1.2 mmol, 1.2 equiv.), aryl

bromide **3a** (188 mg, 1.2 mmol, 1.2 equiv.), $NiCl_2(PCy_3)_2$ (14 mg, 0.02 mmol, 0.02 equiv.), ZrN (421 mg, 4 mmol, 4 equiv.). and 1,4-dioxane (0.2 mL). The purification was accomplished by flash chromatography on silica gel to provide the desired product **4b** (143 mg, 0.85 mmol, 85%).
Yellowish solid, mp 44-45 °C. **^{1}H NMR (500 MHz, DMSO-*d*$_6$):** δ 2.30 (s, 3H, CH_3), 7.22 (d, 2H, ^3J = 8.0 Hz, CH_{Ar}), 7.30 (t, 1H, ^3J = 7.4 Hz, CH_{Ar}), 7.40 (t, 2H, ^3J = 7.7 Hz, CH_{Ar}), 7.51 (d, 2H, ^3J = 8.1 Hz, CH_{Ar}), 7.59 (d, 2H, ^3J = 8.4 Hz, CH_{Ar}).
^{13}C NMR (126 MHz, DMSO-*d*$_6$): δ 21.1, 126.9, 127.0, 127.6, 129.3, 130.0, 137.2, 137.8, 140.6.
Anal. calcd. for $C_{13}H_{12}$: C, 92.81; H, 7.19. Found: C, 92.69; H, 7.31.

4-ethyl-1,1'-biphenyl **(4c)**

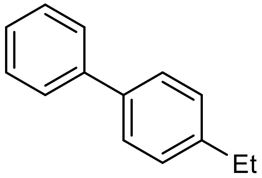

The title compound was prepared starting from phenol **1h** (94 mg, 1 mmol, 1 equiv.), Cp*Ru(Napht)BF_4 (22.6 mg, 0.05 mmol, 0.05 equiv.), bis(pinacolato)diboron (279 mg, 1.1 mmol, 1.1 equiv.), NaF (50 mg, 1.2 mmol, 1.2 equiv.), DABCO (135 mg, 1.2 mmol, 1.2 equiv.), aryl bromide **3c** (205 mg, 1.2 mmol, 1.2 equiv.), $NiCl_2(PCy_3)_2$ (14 mg, 0.02 mmol, 0.02 equiv.), ZrN (421 mg, 4 mmol, 4 equiv.). and 1,4-dioxane (0.2 mL). The purification was accomplished by flash chromatography on silica gel to provide the desired product **4c** (148 mg, 0.88 mmol, 88%).
White solid, mp 34-35 °C **^{1}H NMR (400 MHz, DMSO-*d*$_6$):** δ 1.17 (t, 3H, ^3J = 7.6 Hz, CH_3), 2.60 (q, 2H, ^3J = 7.4 Hz, CH_2), 7.25 – 7.31 (m, 3H, CH_{Ar}), 7.40 (t, 2H, ^3J = 7.6 Hz, CH_{Ar}), 7.53 (dd, 2H, ^3J = 8.0 Hz, ^4J = 2.5 Hz,CH_{Ar}), 7.59 (d, 2H, ^3J = 8.0 Hz, CH_{Ar}).
^{13}C NMR (100 MHz, DMSO-*d*$_6$): δ 16.1, 28.3, 127.0, 127.1, 127.6, 128.8, 129.4, 138.1, 140.6, 143.5.
MS (GC, 70eV): m/z (%) = 182 (M^+, 48), 167 (100), 152 (15).
Anal. calcd. for $C_{14}H_{14}$: C, 92.26; H, 7.74. Found: C, 92.13; H, 7.87.

4'-ethyl-[1,1'-biphenyl]-4-carbonitrile **(4d)**

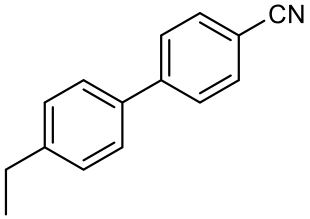

The title compound was prepared starting from phenol **1i** (122 mg, 1 mmol, 1 equiv.), Cp*Ru(Napht)BF_4 (22.6 mg, 0.05 mmol, 0.05 equiv.), bis(pinacolato)diboron (279 mg, 1.1 mmol, 1.1 equiv.), NaF (50 mg, 1.2 mmol, 1.2 equiv.), DABCO (135 mg, 1.2 mmol, 1.2 equiv.), aryl bromide **3d**

(218 mg, 1.2 mmol, 1.2 equiv.), $NiCl_2(PCy_3)_2$ (14 mg, 0.02 mmol, 0.02 equiv.), ZrN (421 mg, 4 mmol, 4 equiv.). and 1,4-dioxane (0.2 mL). The purification was accomplished by flash chromatography on silica gel to provide the desired product **4d** (180 mg, 0.87 mmol, 87%).
White solid, mp 74-75 °C. **^{1}H NMR (500 MHz, $CDCl_3$):** δ 1.30 (t, 3H, ^3J = 7.6 Hz, CH_3), 2.73 (q, 2H, ^3J = 7.6 Hz, CH_2), 7.33 (d, 2H, ^3J = 8.0 Hz, CH_{Ar}), 7.53 (d, 2H, ^3J = 8.2 Hz, CH_{Ar}), 7.67 (d, 2H, ^3J = 8.4 Hz, CH_{Ar}), 7.71 (d, 2H, ^3J = 8.3 Hz, CH_{Ar}).
^{13}C NMR (126 MHz, $CDCl_3$): δ 15.6, 28.6, 110.6, 119.1, 127.2, 127.5, 128.7, 132.6, 136.5, 145.1, 145.6.
MS (GC, 70eV): m/z (%) = 207 (M^+, 48), 192 (100).
Anal. calcd. for $C_{15}H_{13}N$: C, 86.92; H, 6.32; N, 6.76. Found: C, 86.79; H, 6.13; N, 7.08.

4'-butyl-[1,1'-biphenyl]-4-carbonitrile **(4e)**

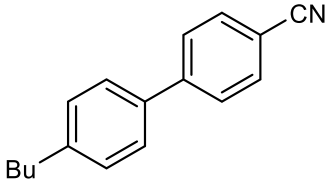

The title compound was prepared starting from phenol **1j** (119 mg, 1 mmol, 1 equiv.), Cp*Ru(Napht)BF_4 (22.6 mg, 0.05 mmol, 0.05 equiv.), bis(pinacolato)diboron (279 mg, 1.1 mmol, 1.1 equiv.), NaF (50 mg, 1.2 mmol, 1.2 equiv.), DABCO (135 mg, 1.2 mmol, 1.2 equiv.), aryl bromide **3d** (218 mg, 1.2 mmol, 1.2 equiv.), $NiCl_2(PCy_3)_2$ (14 mg, 0.02 mmol, 0.02 equiv.), ZrN (421 mg, 4 mmol, 4 equiv.). and 1,4-dioxane (0.2 mL). The purification was accomplished by flash chromatography on silica gel to provide the desired product **4e** (197 mg, 0.84 mmol, 84%).
White solid, mp 50-51 °C. **^{1}H NMR (500 MHz, DMSO-d_6):** δ 0.88 (t, 3H, ^3J = 7.2 Hz, CH_3), 1.31 (sextet, 2H, ^3J = 7.2 Hz, CH_2), 1.55 (sextet, 2H, ^3J = 8.1 Hz, CH_2), 2.60 (t, 2H, ^3J = 8.0 Hz, CH_2), 7.29 (d, 2H, ^3J = 8.0 Hz, CH_{Ar}), 7.63 (d, 2H, ^3J = 8.4 Hz, CH_{Ar}), 7.83 (d, 2H, ^3J = 8.5 Hz, CH_{Ar}), 7.88 (d, 2H, ^3J = 8.5 Hz, CH_{Ar}).
^{13}C NMR (126 MHz, DMSO-d_6): δ 13.7, 21.8, 33.0, 34.4, 109.7, 118.9, 126.9, 127.3, 129.1, 132.8, 135.6, 143.2, 144.6.
Anal. calcd. for $C_{17}H_{17}N$: C, 86.77; H, 7.28; N, 5.95. Found: C, 86.86; H, 7.18; N, 5.96.

[1,1'-biphenyl]-2-carbonitrile **(4f)**

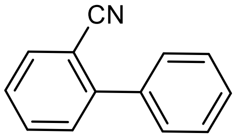

The title compound was prepared starting from phenol **1k** (119 mg, 1 mmol, 1 equiv.), Cp*Ru(Napht)BF_4 (22.6 mg, 0.05 mmol, 0.05 equiv.), bis(pinacolato)diboron (279 mg, 1.1 mmol, 1.1 equiv.), NaF (50 mg, 1.2 mmol, 1.2 equiv.), DABCO (135 mg, 1.2 mmol, 1.2 equiv.), aryl bromide **3a** (188 mg, 1.2 mmol, 1.2 equiv.), $NiCl_2(PCy_3)_2$ (14 mg, 0.02 mmol, 0.02 equiv.), ZrN (421 mg, 4 mmol, 4 equiv.). and 1,4-dioxane (0.2 mL). The purification was accomplished by flash chromatography on silica gel to provide the desired product **4f** (145 mg, 0.81 mmol, 81%).

Alternatively, the title compound was prepared starting from phenol **1h** (94 mg, 1 mmol, 1 equiv.), Cp*Ru(Napht)BF_4 (22.6 mg, 0.05 mmol, 0.05 equiv.), bis(pinacolato)diboron (279 mg, 1.1 mmol, 1.1 equiv.), NaF (50 mg, 1.2 mmol, 1.2 equiv.), DABCO (135 mg, 1.2 mmol, 1.2 equiv.), aryl bromide **3f** (218 mg, 1.2 mmol, 1.2 equiv.), $NiCl_2(PCy_3)_2$ (14 mg, 0.02 mmol, 0.02 equiv.), ZrN (421 mg, 4 mmol, 4 equiv.). and 1,4-dioxane (0.2 mL). The purification was accomplished by flash chromatography on silica gel to provide the desired product **4f** (140 mg, 0.78 mmol, 78%).
White solid, mp 35-37 ^oC. **^{1}H NMR (400 MHz, $CDCl_3$):** δ 7.41-7.50 (m, 5H, CH_{Ar}), 7.56 (dd, 2H, ^3J = 8.4 Hz, ^4J = 1.5 Hz, CH_{Ar}), 7.63 (td, 1H, ^3J = 7.7 Hz, ^4J = 1.2 Hz, CH_{Ar}), 7.76 (dd, 1H, ^3J = 7.7 Hz, ^4J = 1.0 Hz, CH_{Ar})
^{13}C NMR (100 MHz, $CDCl_3$): 111.3, 118.7, 127.5, 128.7, 128.8, 130.1, 132.8, 133.7, 138.1, 145.5.
MS (GC, 70eV): m/z (%) = 179 (M^+, 100), 151 (12).
Anal. calcd. for $C_{13}H_9N$: C, 87.12; H, 5.06; N, 7.82. Found: C, 87.21; H, 5.13; N, 7.66.

[1,1'-biphenyl]-3-carbonitrile **(4g)**

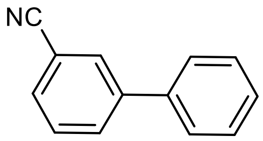

The title compound was prepared starting from phenol **1l** (119 mg, 1 mmol, 1 equiv.), Cp*Ru(Napht)BF_4 (22.6 mg, 0.05 mmol, 0.05 equiv.), bis(pinacolato)diboron (279 mg, 1.1 mmol, 1.1 equiv.), NaF (50 mg, 1.2 mmol, 1.2 equiv.), DABCO (135 mg, 1.2 mmol, 1.2 equiv.), aryl bromide **3a** (188 mg, 1.2 mmol, 1.2 equiv.), $NiCl_2(PCy_3)_2$ (14 mg, 0.02 mmol, 0.02 equiv.), ZrN (421 mg, 4 mmol, 4 equiv.). and 1,4-dioxane (0.2 mL). The purification was accomplished by flash chromatography on silica gel to provide the desired product **4g** (161 mg, 0.90 mmol, 90%).
Alternatively, the title compound was prepared starting from phenol **1h** (94 mg, 1 mmol, 1 equiv.), Cp*Ru(Napht)BF_4 (22.6 mg, 0.05 mmol, 0.05 equiv.), bis(pinacolato)diboron (279 mg, 1.1 mmol, 1.1 equiv.), NaF (50 mg, 1.2 mmol, 1.2 equiv.), DABCO (135 mg, 1.2 mmol, 1.2 equiv.), aryl bromide **3g** (218 mg, 1.2 mmol, 1.2 equiv.), $NiCl_2(PCy_3)_2$ (14 mg, 0.02 mmol, 0.02 equiv.), ZrN (421 mg, 4 mmol, 4 equiv.). and 1,4-dioxane (0.2 mL). The purification was accomplished by flash chromatography on silica gel to provide the desired product **4g** (140 mg, 0.87 mmol, 87%).
White solid, mp 45-46 ^oC. **^{1}H NMR (400 MHz, $CDCl_3$):** δ 7.40 (t, 1H, ^3J = 7.2 Hz, CH_{Ar}), 7.47 (t, 2H, ^3J = 7.7 Hz, CH_{Ar}), 7.55 (dd, 3H, ^3J = 7.8 Hz, ^4J = 2.5 Hz, CH_{Ar}), 7.61 (d, 1H, ^3J = 7.7 Hz, CH_{Ar}), 7.80 (d, 1H, ^3J = 7.8 Hz, CH_{Ar}), 7.85 (s, 1H, CH_{Ar}).
^{13}C NMR (100 MHz, $CDCl_3$): 112.9, 118.8, 127.1, 128.4, 129.1, 129.6, 130.7, 131.5, 138.8, 142.5.
MS (GC, 70eV): m/z (%) = 179 (M^+, 100), 151 (12).
Anal. calcd. for $C_{13}H_9N$: C, 87.12; H, 5.06; N, 7.82. Found: C, 87.06; H, 5.18; N, 7.76.

4'-chloro-[1,1'-biphenyl]-4-carboxylic acid **(4h)**

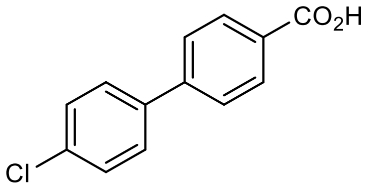

The title compound was prepared starting from phenol **1m** (128 mg, 1 mmol, 1 equiv.), Cp*Ru(Napht)BF_4 (22.6 mg, 0.05 mmol, 0.05 equiv.), bis(pinacolato)diboron (279 mg, 1.1 mmol, 1.1 equiv.), NaF (50 mg, 1.2 mmol, 1.2 equiv.), DABCO (135 mg, 1.2 mmol, 1.2 equiv.), aryl bromide **3h** (241 mg, 1.2 mmol, 1.2 equiv.), $NiCl_2(PCy_3)_2$ (14 mg, 0.02 mmol, 0.02 equiv.), ZrN (421 mg, 4 mmol, 4 equiv.). and 1,4-dioxane (0.2 mL). The purification was accomplished by flash chromatography on silica gel to provide the desired product **4h** (192 mg, 0.83 mmol, 83%).

White solid, mp >220 °C. **^{1}H NMR (400 MHz, DMSO-*d*$_6$):** δ 7.56 (d, 2H, ^3J = 8.5 Hz, CH_{Ar}), 7.77 (d, 2H, ^3J = 8.5 Hz, CH_{Ar}), 7.81 (d, 2H, ^3J = 8.3 Hz, CH_{Ar}), 8.04 (d, 2H, ^3J = 8.3 Hz, CH_{Ar}), 13.03 (s, 1H, COOH).

^{13}C NMR (100 MHz, DMSO-*d*$_6$): δ 127.3, 129.2, 129.5, 130.5, 133.7, 138.3, 143.4, 167.5.

Anal. calcd. for $C_{13}H_9ClO_2$: C, 67.11; H, 3.90. Found: C, 67.23; H, 3.81.

4'-fluoro-[1,1'-biphenyl]-2-carboxylic acid **(4i)**

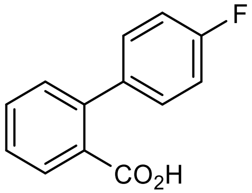

The title compound was prepared starting from phenol **1n** (138 mg, 1 mmol, 1 equiv.), Cp*Ru(Napht)BF_4 (22.6 mg, 0.05 mmol, 0.05 equiv.), bis(pinacolato)diboron (279 mg, 1.1 mmol, 1.1 equiv.), NaF (50 mg, 1.2 mmol, 1.2 equiv.), DABCO (135 mg, 1.2 mmol, 1.2 equiv.), aryl bromide **3i** (210 mg, 1.2 mmol, 1.2 equiv.), $NiCl_2(PCy_3)_2$ (14 mg, 0.02 mmol, 0.02 equiv.), ZrN (421 mg, 4 mmol, 4 equiv.). and 1,4-dioxane (0.2 mL). The purification was accomplished by flash chromatography on silica gel to provide the desired product **4i** (161 mg, 0.90 mmol, 90%).

Alternatively, the title compound was prepared starting from phenol **1o** (112 mg, 1 mmol, 1 equiv.), Cp*Ru(Napht)BF_4 (22.6 mg, 0.05 mmol, 0.05 equiv.), bis(pinacolato)diboron (279 mg, 1.1 mmol, 1.1 equiv.), NaF (50 mg, 1.2 mmol, 1.2 equiv.), DABCO (135 mg, 1.2 mmol, 1.2 equiv.), aryl bromide **3j** (241 mg, 1.2 mmol, 1.2 equiv.), $NiCl_2(PCy_3)_2$ (14 mg, 0.02 mmol, 0.02 equiv.), ZrN (421 mg, 4 mmol, 4 equiv.). and 1,4-dioxane (0.2 mL). The purification was accomplished by flash chromatography on silica gel to provide the desired product **4i** (161 mg, 0.72 mmol, 72%).

White solid, mp >225 °C. **^{1}H NMR (400 MHz, $CDCl_3$):** δ 7.04 – 7.09 (m, 2H, CH_{Ar}), 7.26 – 7.29 (m, 2H, CH_{Ar}), 7.33 (dd, 1H, ^3J = 7.6 Hz, ^4J = 1.0 Hz, CH_{Ar}), 7.42 (td, 1H, ^3J = 7.6 Hz, ^4J = 1.2 Hz, CH_{Ar}), 7.55 (td, 1H, ^3J = 7.5 Hz, ^4J = 1.4 Hz, CH_{Ar}), 7.96 (dd, 1H, ^3J = 7.8 Hz, ^4J = 1.2 Hz, CH_{Ar}).

^{13}C NMR (100 MHz, $CDCl_3$): δ 114.1 (d, J_{CF} = 21.2 Hz), 127.4, 129.1, 130.1 (d, J_{CF} = 8.1 Hz), 130.8, 131.2, 132.2, 137.0 (d, J_{CF} = 3.4 Hz), 142.5, 163.6 (d, $^1J_{CF}$ = 244.7 Hz), 172.9.

Anal. calcd. for $C_{13}H_9FO_2$: C, 72.22; H, 4.20. Found: C, 87.06; H, 5.18.

4'-methyl-[1,1'-biphenyl]-3-carboxylic acid **(4j)**

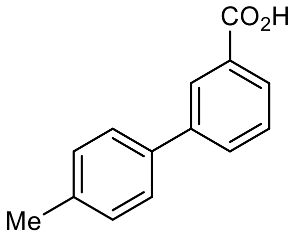

The title compound was prepared starting from phenol **1a** (108 mg, 1 mmol, 1 equiv.), Cp*Ru(Napht)BF_4 (22.6 mg, 0.05 mmol, 0.05 equiv.), bis(pinacolato)diboron (279 mg, 1.1 mmol, 1.1 equiv.), NaF (50 mg, 1.2 mmol, 1.2 equiv.), DABCO (135 mg, 1.2 mmol, 1.2 equiv.), aryl bromide **3k** (241 mg, 1.2 mmol, 1.2 equiv.), $NiCl_2(PCy_3)_2$ (14 mg, 0.02 mmol, 0.02 equiv.), ZrN (421 mg, 4 mmol, 4 equiv.). and 1,4-dioxane (0.2 mL). The purification was accomplished by flash chromatography on silica gel to provide the desired product **4j** (170 mg, 0.80 mmol, 80%).
Alternatively, the title compound was prepared starting from phenol **1p** (138 mg, 1 mmol, 1 equiv.), Cp*Ru(Napht)BF_4 (22.6 mg, 0.05 mmol, 0.05 equiv.), bis(pinacolato)diboron (279 mg, 1.1 mmol, 1.1 equiv.), NaF (50 mg, 1.2 mmol, 1.2 equiv.), DABCO (135 mg, 1.2 mmol, 1.2 equiv.), aryl bromide **3b** (205 mg, 1.2 mmol, 1.2 equiv.), $NiCl_2(PCy_3)_2$ (14 mg, 0.02 mmol, 0.02 equiv.), ZrN (421 mg, 4 mmol, 4 equiv.). and 1,4-dioxane (0.2 mL). The purification was accomplished by flash chromatography on silica gel to provide the desired product **4j** (161 mg, 0.82 mmol, 82%).
White solid, mp 132-133 °C. **^{1}H NMR (400 MHz, $CDCl_3$):** δ 2.41 (s, 3H, CH_3), 7.28 (d, 2H, ^3J = 7.8 Hz, CH_{Ar}), 7.54 (dd, 3H, ^3J = 7.8 Hz, ^4J = 2.5 Hz, CH_{Ar}), 7.81 – 7.84 (m, 1H, CH_{Ar}), 8.08 (dt, 1H, ^3J = 7.8 Hz, ^4J = 1.2 Hz, CH_{Ar}), 8.35 (t, 1H, ^4J = 1.6 Hz, CH_{Ar}).
^{13}C NMR (100 MHz, $CDCl_3$): 21.4, 127.0, 128.6, 128.7, 128.9, 129.7, 129.8, 132.2, 130.1, 137.4, 141.6, 172.1.
Anal. calcd. for $C_{14}H_{12}O_2$: C, 79.23; H, 5.70. Found: C, 79.11; H, 5.76.

3-(pyrimidin-5-yl)benzoic acid **(4k)**

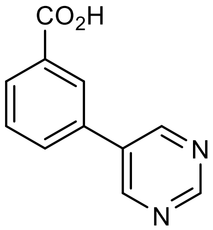

The title compound was prepared starting from phenol **1p** (138 mg, 1 mmol, 1 equiv.), Cp*Ru(Napht)BF_4 (22.6 mg, 0.05 mmol, 0.05 equiv.), bis(pinacolato)diboron (279 mg, 1.1 mmol, 1.1 equiv.), NaF (50 mg, 1.2 mmol, 1.2 equiv.), DABCO (135 mg, 1.2 mmol, 1.2 equiv.), aryl bromide **3l**

(190 mg, 1.2 mmol, 1.2 equiv.), $NiCl_2(PCy_3)_2$ (14 mg, 0.02 mmol, 0.02 equiv.), ZrN (421 mg, 4 mmol, 4 equiv.). and 1,4-dioxane (0.2 mL). The purification was accomplished by flash chromatography on silica gel to provide the desired product **4k** (152 mg, 0.76 mmol, 76%).

White solid, mp 188-189 **^{1}H NMR (400 MHz, DMSO-d_6):** δ 7.68 (t, 1H, ^3J = 7.7 Hz, CH_{Ar}), 8.04 – 8.07 (m, 2H, CH_{Ar}), 8.29 (s, 1H, CH_{Ar}), 9.18 (s, 2H, CH_{Ar}), 9.23 (s, 1H, CH_{Ar}), 13.21 (s, 1H, COOH).

^{13}C NMR (100 MHz, DMSO-d_6): 128.1, 130.0, 130.1, 131.9, 132.4, 133.1, 134.8, 155.4, 158.1, 167.4.

Anal. calcd. for $C_{14}H_{12}O_2$: C, 79.23; H, 5.70. Found: C, 79.11; H, 5.76.

***4-benzyl-1,1'-biphenyl* (4l)**

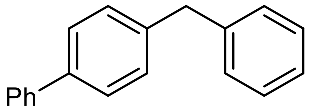

The title compound was prepared starting from phenol **1q** (184 mg, 1 mmol, 1 equiv.), Cp*Ru(Napht)BF_4 (22.6 mg, 0.05 mmol, 0.05 equiv.), bis(pinacolato)diboron (279 mg, 1.1 mmol, 1.1 equiv.), NaF (50 mg, 1.2 mmol, 1.2 equiv.), DABCO (135 mg, 1.2 mmol, 1.2 equiv.), aryl bromide **3a** (188 mg, 1.2 mmol, 1.2 equiv.), $NiCl_2(PCy_3)_2$ (14 mg, 0.02 mmol, 0.02 equiv.), ZrN (421 mg, 4 mmol, 4 equiv.). and 1,4-dioxane (0.2 mL). The purification was accomplished by flash chromatography on silica gel to provide the desired product **4l** (212 mg, 0.87 mmol, 87%).

White solid, mp 86-87 ^{o}C. **^{1}H NMR (500 MHz, DMSO-d_6):** δ 3.93 (s, 2H, CH_2), 7.15 (t, 1H, ^3J = 7.0 Hz, CH_{Ar}), 7.21 – 7.22 (m, 3H, CH_{Ar}), 7.25 – 7.31 (m, 4H, CH_{Ar}), 7.40 (t, 2H, ^3J = 7.8 Hz, CH_{Ar}), 7.54 (d, 2H, ^3J = 8.2 Hz, CH_{Ar}), 7.58 (d, 2H, ^3J = 8.4 Hz, CH_{Ar}).

^{13}C NMR (126 MHz DMSO-d_6): δ 41.2, 126.5, 127.0, 127.2, 127.7, 128.9, 129.2, 129.4, 129.7, 138.4, 140.5, 141.1, 141.7.

MS (GC, 70eV): m/z (%) = 244 (M^+, 100), 165 (35).

Anal. calcd. for $C_{19}H_{16}$: C, 93.40; H, 60.60. Found: C, 79.11; H, 5.76.

***(3'-fluoro-[1,1'-biphenyl]-3-yl)methanol* (4m)**

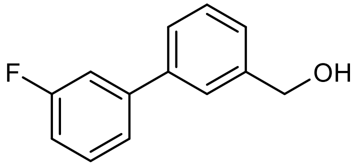

The title compound was prepared starting from phenol **1r** (112 mg, 1 mmol, 1 equiv.), Cp*Ru(Napht)BF_4 (22.6 mg, 0.05 mmol, 0.05 equiv.), bis(pinacolato)diboron (279 mg, 1.1 mmol, 1.1 equiv.), NaF (50 mg, 1.2 mmol, 1.2 equiv.), DABCO (135 mg, 1.2 mmol, 1.2 equiv.), aryl bromide **3m** (241 mg, 1.2 mmol, 1.2 equiv.), $NiCl_2(PCy_3)_2$ (14 mg, 0.02 mmol, 0.02 equiv.), ZrN (421 mg, 4 mmol, 4 equiv.). and 1,4-dioxane (0.2 mL). The purification was accomplished by flash chromatography on silica gel to provide the desired product **4m** (167 mg, 0.83 mmol, 83%).

Alternatively, the title compound was prepared starting from phenol **1s** (124 mg, 1 mmol, 1 equiv.), Cp*Ru(Napht)BF_4 (22.6 mg, 0.05 mmol, 0.05 equiv.), bis(pinacolato)diboron (279 mg, 1.1 mmol, 1.1 equiv.), NaF (50 mg, 1.2 mmol, 1.2 equiv.), DABCO (135 mg, 1.2 mmol, 1.2 equiv.), aryl

bromide **3n** (210 mg, 1.2 mmol, 1.2 equiv.), $NiCl_2(PCy_3)_2$ (14 mg, 0.02 mmol, 0.02 equiv.), ZrN (421 mg, 4 mmol, 4 equiv.). and 1,4-dioxane (0.2 mL). The purification was accomplished by flash chromatography on silica gel to provide the desired product **4m** (174 mg, 0.86 mmol, 86%).
Oringe oli. **^{1}H NMR (400 MHz, CDCl$_3$):** δ 1.87 (s, 1H, OH) 4.76 (d, 2H, ^3J = 4.5 Hz, CH_2), 7.01 – 7.06 (m, 1H, CH_{Ar}), 7.28 (dt, 1H, ^3J = 10.1 Hz, ^4J = 2.0 Hz, CH_{Ar}), 7.35 – 7.38 (m, 3H, CH_{Ar}), 7.43 (t, 1H, ^3J = 7.6 Hz, CH_{Ar}), 7.49 (d, 1H, ^3J = 7.7 Hz, CH_{Ar}), 7.57 (s, 1H, CH_{Ar}).
^{13}C NMR (100 MHz, CDCl$_3$): δ 65.2, 114.1 (dd, J_{CF} = 21.2 Hz, J_{CF} = 12.4 Hz), 122.8 (d, J_{CF} = 3.0 Hz), 125.7, 126.4, 127.0, 128.6, 129.1, 130.2 (d, J_{CF} = 8.24 Hz), 140.3 (d, J_{CF} = 2.0 Hz), 141.5, 143.3 (d, J_{CF} = 7.7 Hz), 163.2 (d, $^1J_{CF}$ = 245.6 Hz).
^{19}F NMR (376 MHz, CDCl$_3$): δ -113.06 (s, 1F).
MS (GC, 70eV): m/z (%) = 244 (M^+, 100), 165 (35).
Anal. calcd. for $C_{19}H_{16}$: C, 93.40; H, 60.60. Found: C, 79.11; H, 5.76.

N-phenyl-[1,1'-biphenyl]-4-amine (4n)

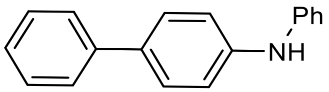

The title compound was prepared starting from phenol **1t** (185 mg, 1 mmol, 1 equiv.), Cp*Ru(Napht)BF_4 (22.6 mg, 0.05 mmol, 0.05 equiv.), bis(pinacolato)diboron (279 mg, 1.1 mmol, 1.1 equiv.), NaF (50 mg, 1.2 mmol, 1.2 equiv.), DABCO (135 mg, 1.2 mmol, 1.2 equiv.), aryl bromide **3a** (188 mg, 1.2 mmol, 1.2 equiv.), $NiCl_2(PCy_3)_2$ (14 mg, 0.02 mmol, 0.02 equiv.), ZrN (421 mg, 4 mmol, 4 equiv.). and 1,4-dioxane (0.2 mL). The purification was accomplished by flash chromatography on silica gel to provide the desired product **4n** (206 mg, 0.84 mmol, 84%).
White solid, mp 112-113 ^{o}C. **^{1}H NMR (400 MHz, CDCl$_3$):** δ 5.57 (s, 1H, NH), 6.94 (t, 1H, ^3J = 7.3 Hz, CH_{Ar}), 7.11 (t, 4H, ^3J = 8.4 Hz, CH_{Ar}), 7.29 (dd, 3H, ^3J = 7.5 Hz, ^4J = 2.0 Hz, CH_{Ar}), 7.40 (t, 2H, ^3J = 7.5 Hz, CH_{Ar}), 7.50 (d, 2H, ^3J = 8.5 Hz, CH_{Ar}), 7.56 (d, 2H, ^3J = 7.3, CH_{Ar}).
^{13}C NMR (100 MHz, CDCl$_3$): δ 117.8, 118.1, 121.2, 126.5, 126.6, 127.9, 128.7, 129.4, 133.7, 140.8, 142.5, 142.8.
MS (GC, 70eV): m/z (%) = 245 (M^+, 100).
Anal. calcd. for $C_{18}H_{15}N$: C, 88.13; H, 6.19; N, 5.71. Found: C, 88.29; H, 6.23; N, 5.48.

[1,1'-biphenyl]-2-amine (4o)

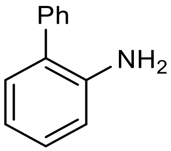

The title compound was prepared starting from phenol **1u** (109 mg, 1 mmol, 1 equiv.), Cp*Ru(Napht)BF_4 (22.6 mg, 0.05 mmol, 0.05 equiv.), bis(pinacolato)diboron (279 mg, 1.1 mmol, 1.1 equiv.), NaF (50 mg, 1.2 mmol, 1.2 equiv.), DABCO (135 mg, 1.2 mmol, 1.2 equiv.), aryl bromide **3a**

(188 mg, 1.2 mmol, 1.2 equiv.), $NiCl_2(PCy_3)_2$ (14 mg, 0.02 mmol, 0.02 equiv.), ZrN (421 mg, 4 mmol, 4 equiv.). and 1,4-dioxane (0.2 mL). The purification was accomplished by flash chromatography on silica gel to provide the desired product **4o** (118 mg, 0.70 mmol, 70%).
Brown solid, mp 48-49 °C. **^{1}H NMR (400 MHz, $CDCl_3$):** δ 3.73 (s, 2H, NH_2), 6.75 (d, 1H, ^3J = 8.0 Hz, CH_{Ar}), 6.81 (t, 1H, ^3J = 7.5 Hz, CH_{Ar}), 7.11 – 7.16 (m, 2H, CH_{Ar}), 7.31 – 7.35 (m, 1H, CH_{Ar}), 7.40 – 7.45 (m, 4H, CH_{Ar}).
^{13}C NMR (100 MHz, $CDCl_3$): 115.5, 118.6, 127.1, 127.6, 128.5, 128.8, 129.1, 130.4, 139.5, 143.5.
MS (GC, 70eV): m/z (%) = 169 (M^+, 100).
Anal. calcd. for $C_{12}H_{11}N$: C, 85.17; H, 6.55; N, 8.29. Found: C, 88.29; H, 6.23; N, 5.48.

3-([1,1'-biphenyl]-4-yl)propanoic acid **(4p)**

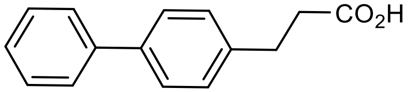

The title compound was prepared starting from phenol **1v** (166 mg, 1 mmol, 1 equiv.), Cp*Ru(Napht)BF_4 (22.6 mg, 0.05 mmol, 0.05 equiv.), bis(pinacolato)diboron (279 mg, 1.1 mmol, 1.1 equiv.), NaF (50 mg, 1.2 mmol, 1.2 equiv.), DABCO (135 mg, 1.2 mmol, 1.2 equiv.), aryl bromide **3a** (188 mg, 1.2 mmol, 1.2 equiv.), $NiCl_2(PCy_3)_2$ (14 mg, 0.02 mmol, 0.02 equiv.), ZrN (421 mg, 4 mmol, 4 equiv.). and 1,4-dioxane (0.2 mL). The purification was accomplished by flash chromatography on silica gel to provide the desired product **4p** (187 mg, 0.83 mmol, 83%).
White solid, mp 149-150 °C. **^{1}H NMR (400 MHz, $CDCl_3$):** δ 2.73 (t, 2H, ^3J = 7.8 Hz, CH_2), 3.01 (t, 2H, ^3J = 7.8 Hz, CH_2), 7.28 (d, 2H, ^3J = 7.9 Hz, CH_{Ar}), 7.33 (d, 1H, ^3J = 7.4 Hz, CH_{Ar}), 7.42 (t, 2H, ^3J = 7.5 Hz, CH_{Ar}), 7.52 (d, 2H, ^3J = 8.0 Hz, CH_{Ar}), 7.57 (d, 2H, ^3J = 7.8 Hz, CH_{Ar}), 11.21 (bs, 1H, COOH).
^{13}C NMR (100 MHz, $CDCl_3$): 30.2, 35.4, 127.0, 127.2, 127.3, 128.7, 128.8, 139.2, 139.4, 140.9, 178.6.
Anal. calcd. for $C_{15}H_{14}O_2$: C, 79.62; H, 6.24. Found: C, 79.73; H, 6.17.

1-([1,1'-biphenyl]-4-yl)ethan-1-ol **(4q)**

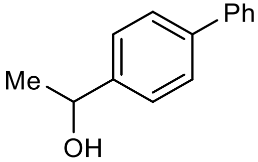

The title compound was prepared starting from phenol **1w** (138 mg, 1 mmol, 1 equiv.), Cp*Ru(Napht)BF_4 (22.6 mg, 0.05 mmol, 0.05 equiv.), bis(pinacolato)diboron (279 mg, 1.1 mmol, 1.1 equiv.), NaF (50 mg, 1.2 mmol, 1.2 equiv.), DABCO (135 mg, 1.2 mmol, 1.2 equiv.), aryl bromide **3a** (188 mg, 1.2 mmol, 1.2 equiv.), $NiCl_2(PCy_3)_2$ (14 mg, 0.02 mmol, 0.02 equiv.), ZrN (421 mg, 4 mmol, 4 equiv.). and 1,4-dioxane (0.2 mL). The purification was accomplished by flash chromatography on silica gel to provide the desired product **4q** (142 mg, 0.72 mmol, 72%).
White solid, mp 96-97 °C. **^{1}H NMR (400 MHz, $CDCl_3$):** δ 1.54 (d, 3H, ^3J = 6.0 Hz, CH_2), 1.91 (s, 1H, OH), 4.96 (q, 1H, ^3J = 6.3 Hz, CH_2), 7.34 – 7.37 (m, 1H, CH_{Ar}), 7.43 – 7.47 (m, 4H, CH_{Ar}), 7.58 – 7.61 (m, 4H, CH_{Ar}).

^{13}C NMR (100 MHz, $CDCl_3$): 25.1, 70.1, 125.8, 127.1, 127.3, 128.8, 140.5, 140.9, 144.8.
MS (GC, 70eV): m/z (%) = 198 (M^+, 53), 183 (89), 180 (31), 155 (100), 152 (42).
Anal. calcd. for $C_{14}H_{14}O$: C, 84.81; H, 7.12. Found: C, 84.69; H, 7.22.

N-cyclohexyl-N-methyl-[1,1'-biphenyl]-4-carboxamide **(4r)**

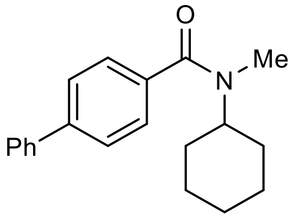

The title compound was prepared starting from phenol **1x** (233 mg, 1 mmol, 1 equiv.), Cp*Ru(Napht)BF_4 (22.6 mg, 0.05 mmol, 0.05 equiv.), bis(pinacolato)diboron (279 mg, 1.1 mmol, 1.1 equiv.), NaF (50 mg, 1.2 mmol, 1.2 equiv.), DABCO (135 mg, 1.2 mmol, 1.2 equiv.), aryl bromide **3a** (188 mg, 1.2 mmol, 1.2 equiv.), $NiCl_2(PCy_3)_2$ (14 mg, 0.02 mmol, 0.02 equiv.), ZrN (421 mg, 4 mmol, 4 equiv.). and 1,4-dioxane (0.2 mL). The purification was accomplished by flash chromatography on silica gel to provide the desired product **4r** (260 mg, 0.89 mmol, 89%).
White solid, mp 105-106 °C. **^1H NMR (400 MHz, DMSO-d_6):** δ 1.00 – 1.78 (m, 10H, Me, CH_2), 2.78 – 2.86 (s, 3H, CH_2), 3.41, 4.30 (s, 1H, CH), 7.37 – 7.49 (m, 5H, CH_{Ar}), 7.71 (m, 4H, CH_{Ar}).
^{13}C NMR (100 MHz, DMSO-d_6): 24.5, 25.2, 27.1, 29.0, 30.1, 31.7, 52.5, 57.7, 79.0 (m), 126.6, 126.7, 127.8, 129.0, 136.2, 139.3, 140.7, 170.1.
MS (GC, 70eV): m/z (%) = 293 (M^+, 24), 211 (18), 181 (100), 152 (37), 111 (15).
Anal. calcd. for $C_0H_{23}ON$: C, 81.87; H, 7.90; N, 4.77. Found: C, 81.93; H, 7.73; N, 4.63.

1,1':4',1''-terphenyl **(4s)**

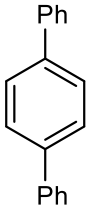

The title compound was prepared starting from phenol **1y** (110 mg, 1 mmol, 1 equiv.), Cp*Ru(Napht)BF_4 (22.6 mg, 0.05 mmol, 0.05 equiv.), bis(pinacolato)diboron (279 mg, 1.1 mmol, 1.1 equiv.), NaF (50 mg, 1.2 mmol, 1.2 equiv.), DABCO (135 mg, 1.2 mmol, 1.2 equiv.), aryl bromide **3a** (377 mg, 2.4 mmol, 2..4 equiv.), $NiCl_2(PCy_3)_2$ (14 mg, 0.02 mmol, 0.02 equiv.), ZrN (421 mg, 4 mmol, 4 equiv.). and 1,4-dioxane (0.2 mL). The purification was accomplished by flash chromatography on silica gel to provide the desired product **4s** (191 mg, 0.83 mmol, 83%).

White solid, mp 212-213 ^{1}**H NMR (400 MHz, CDCl$_3$):** δ 7.35 (t, 2H, ^3J = 7.4 Hz, CH_{Ar}), 7.45 (t, 4H, ^3J = 7.5 Hz, CH_{Ar}), 7.63 (d, 4H, ^3J = 7.8 Hz, CH_{Ar}), 7.67 (s, 4H, CH_{Ar}).

^{13}**C NMR (100 MHz, CDCl$_3$):** 127.0, 127.3, 127.5, 128.8, 140.1, 140.7.

MS (GC, 70eV): m/z (%) = 230 (M^+, 100).

Anal. calcd. for $C_{18}H_{14}$: C, 93.87; H, 6.13. Found: C, 93.79; H, 6.28.

5-chloro-2,3-diphenyl-1H-indole **(4t)**

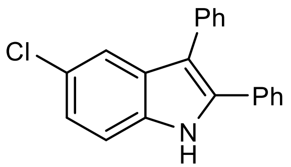

The title compound was prepared starting from phenol **1h** (197 mg, 2.1 mmol, 2.1 equiv.), Cp*Ru(Napht)BF_4 (38 mg, 0.1 mmol, 0.1 equiv.), bis(pinacolato)diboron (558 mg, 2.2 mmol, 2.2 equiv.), NaF (100 mg, 2.4 mmol, 2.4 equiv.), DABCO (270 mg, 2.4 mmol, 2.4 equiv.), aryl bromide **3o** (309 mg, 1.0 mmol, 1.0 equiv.), $NiCl_2(PCy_3)_2$ (28 mg, 0.04 mmol, 0.04 equiv.), ZrN (421 mg, 4 mmol, 4 equiv.). and 1,4-dioxane (0.3 mL). The purification was accomplished by flash chromatography on silica gel to provide the desired product **4t** (194 mg, 0.64 mmol, 64%).

White solid, mp 131-132 ^{o}C. ^{1}**H NMR (400 MHz, CDCl$_3$):** δ 7.18 (dd, 1H, ^3J = 8.5 Hz, ^4J = 2.0 Hz, CH_{Ar}), 7.28 – 7.35 (m, 5H, CH_{Ar}), 7.37 – 7.41 (m, 6H, CH_{Ar}), 7.62 (d, 1H, ^4J = 2.0 Hz, CH_{Ar}), 8.23 (s, 1H, NH).

^{13}**C NMR (100 MHz, CDCl$_3$):** δ 111.9, 114.8, 119.2, 123.0, 126.2, 126.6, 128.0, 128.1, 128.7, 128.8, 129.9, 130.1, 132.2, 134.2, 134.4, 135.4.

MS (GC, 70eV): m/z (%) = 247 (M^+, 100), 230 (14), 217 923), 189 (11).

Anal. calcd. for $C_{20}H_{14}ClN$: C, 79.07; H, 4.65; N, 4.61. Found: C, 79.16; H, 4.58; N, 4.55.

(C) Copies ^{1}H and ^{13}C NMR spectra.

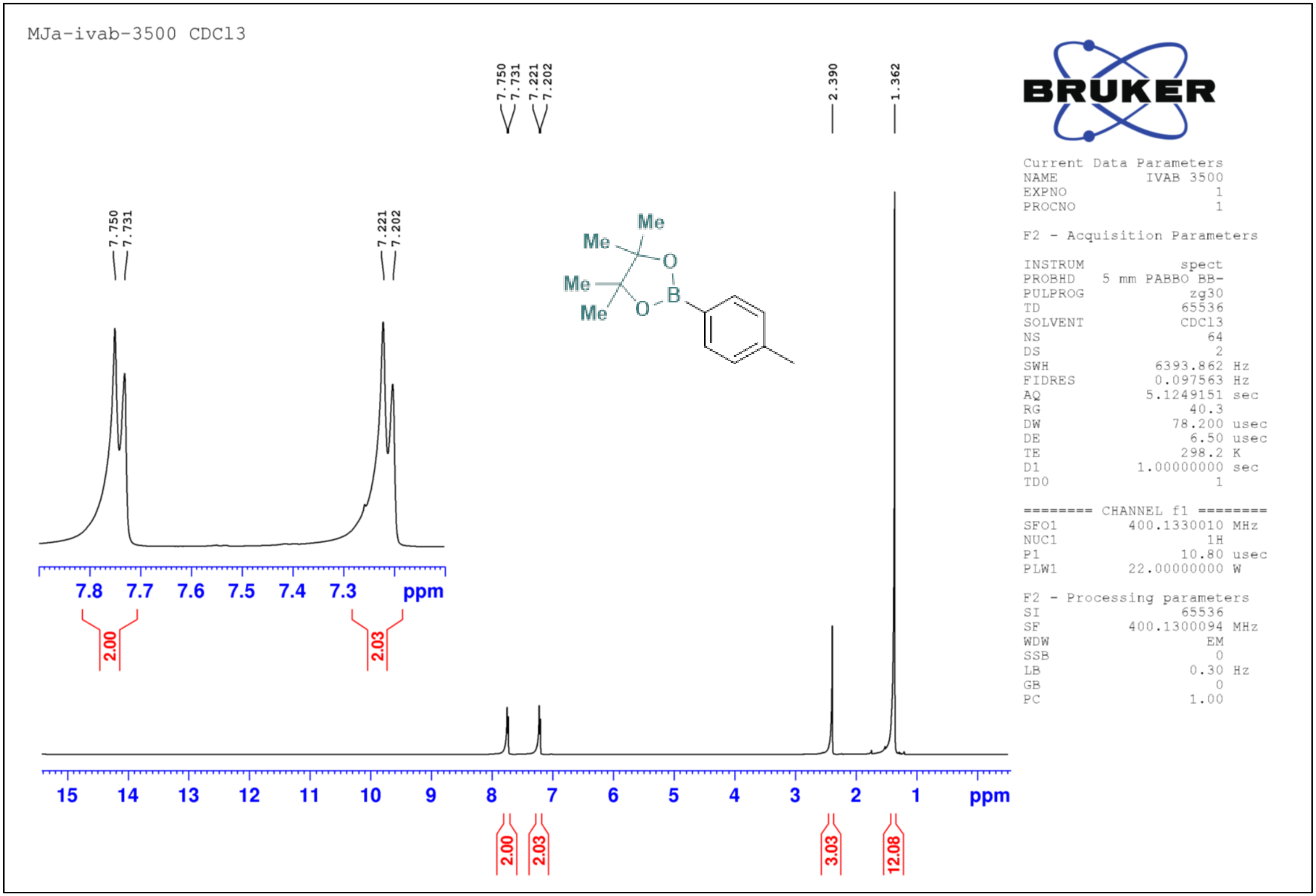

^{1}H NMR Spectrum of Compound 2a

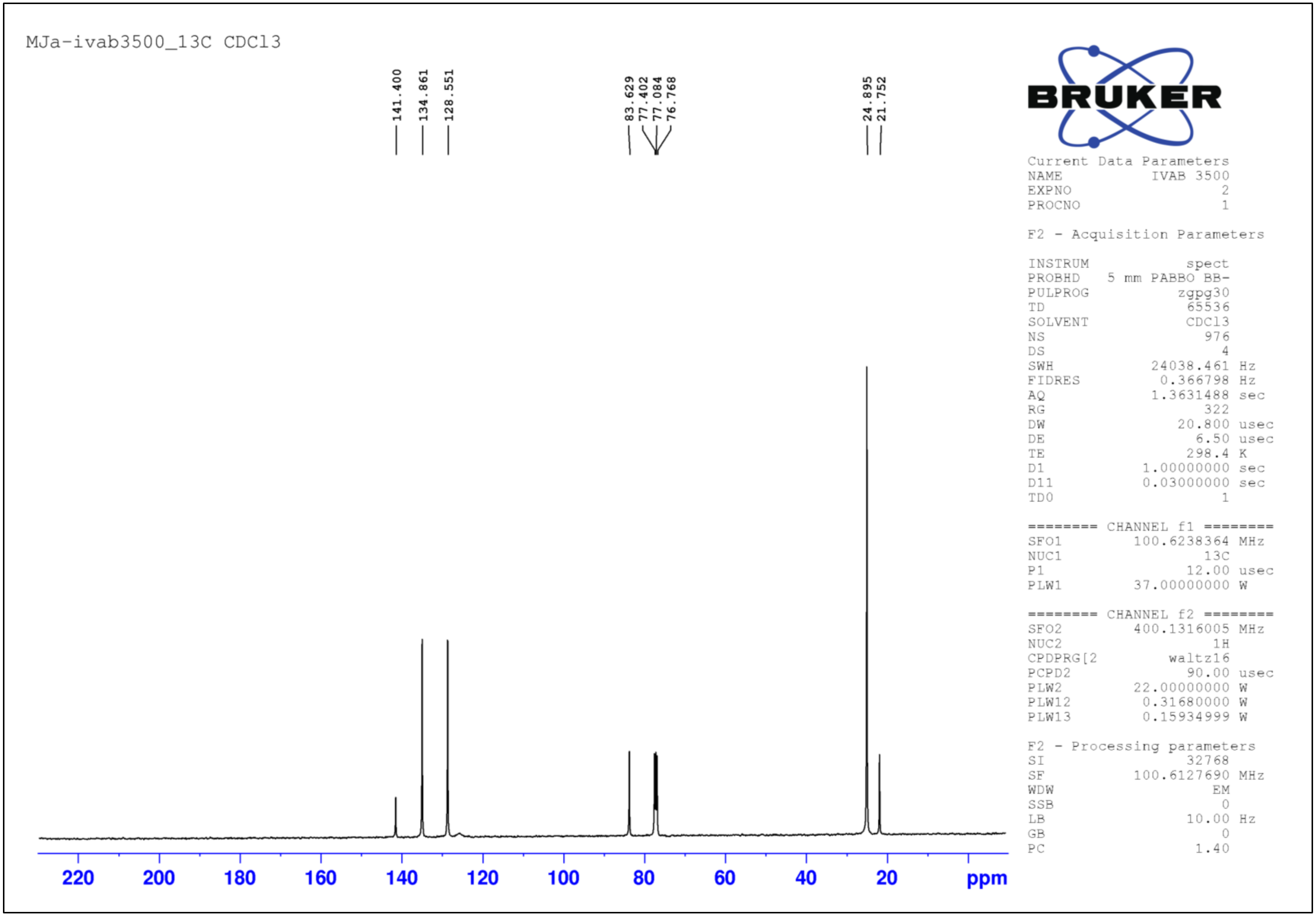

^{13}C NMR Spectrum of Compound 2a

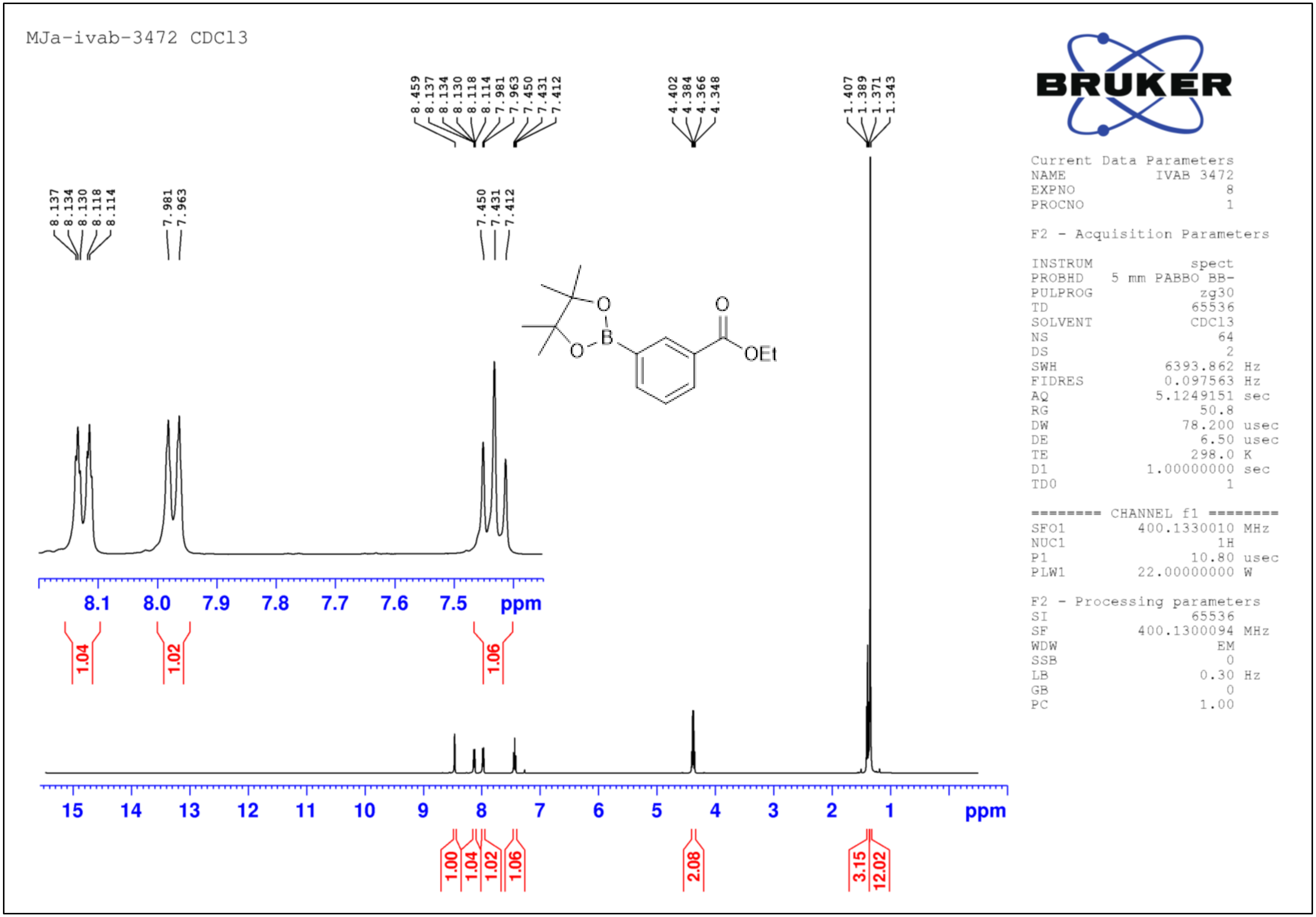

^{1}H NMR Spectrum of Compound 2b

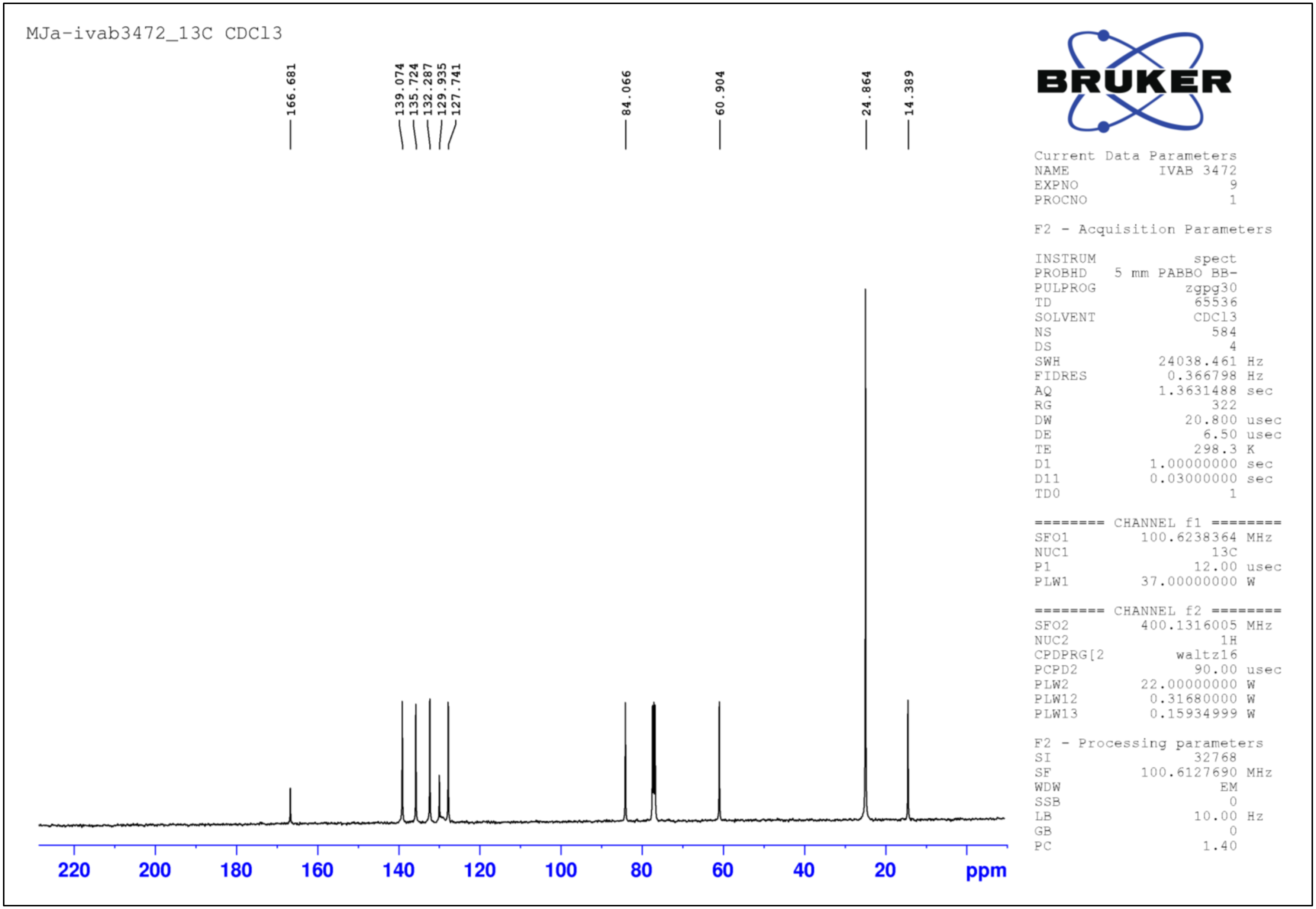

^{13}C NMR Spectrum of Compound 2b

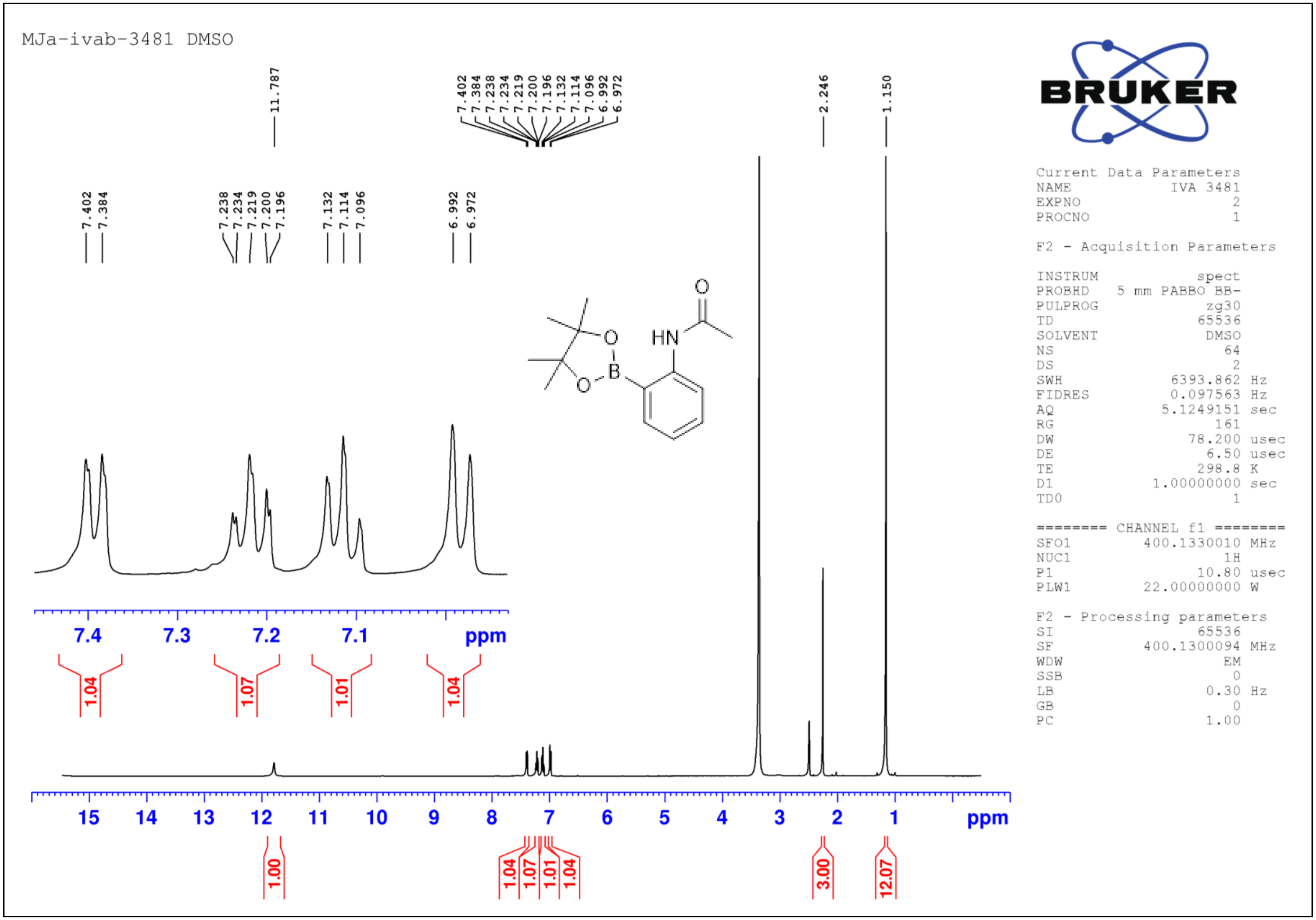

^{1}H NMR Spectrum of Compound 2c

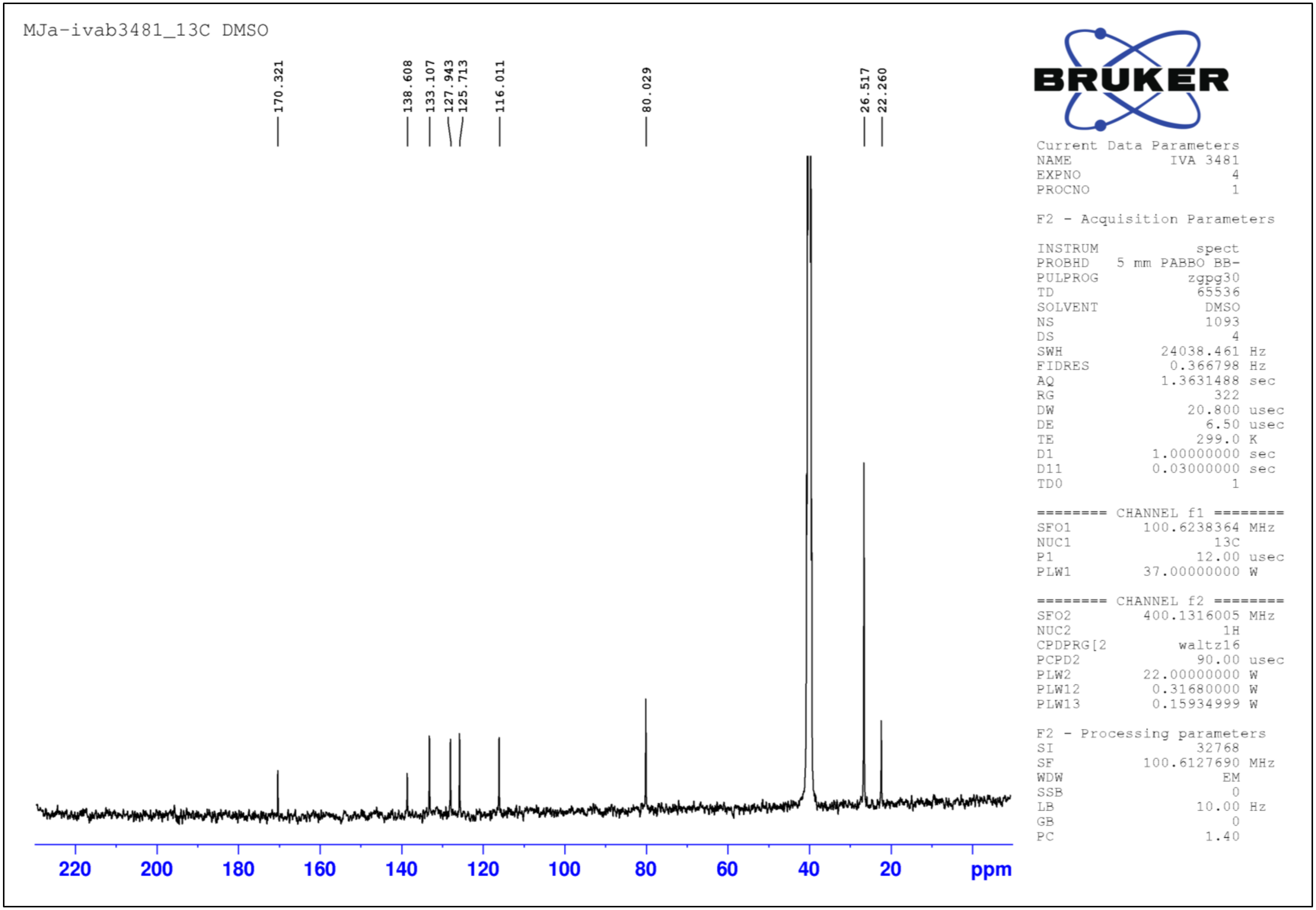

^{13}C NMR Spectrum of Compound 2c

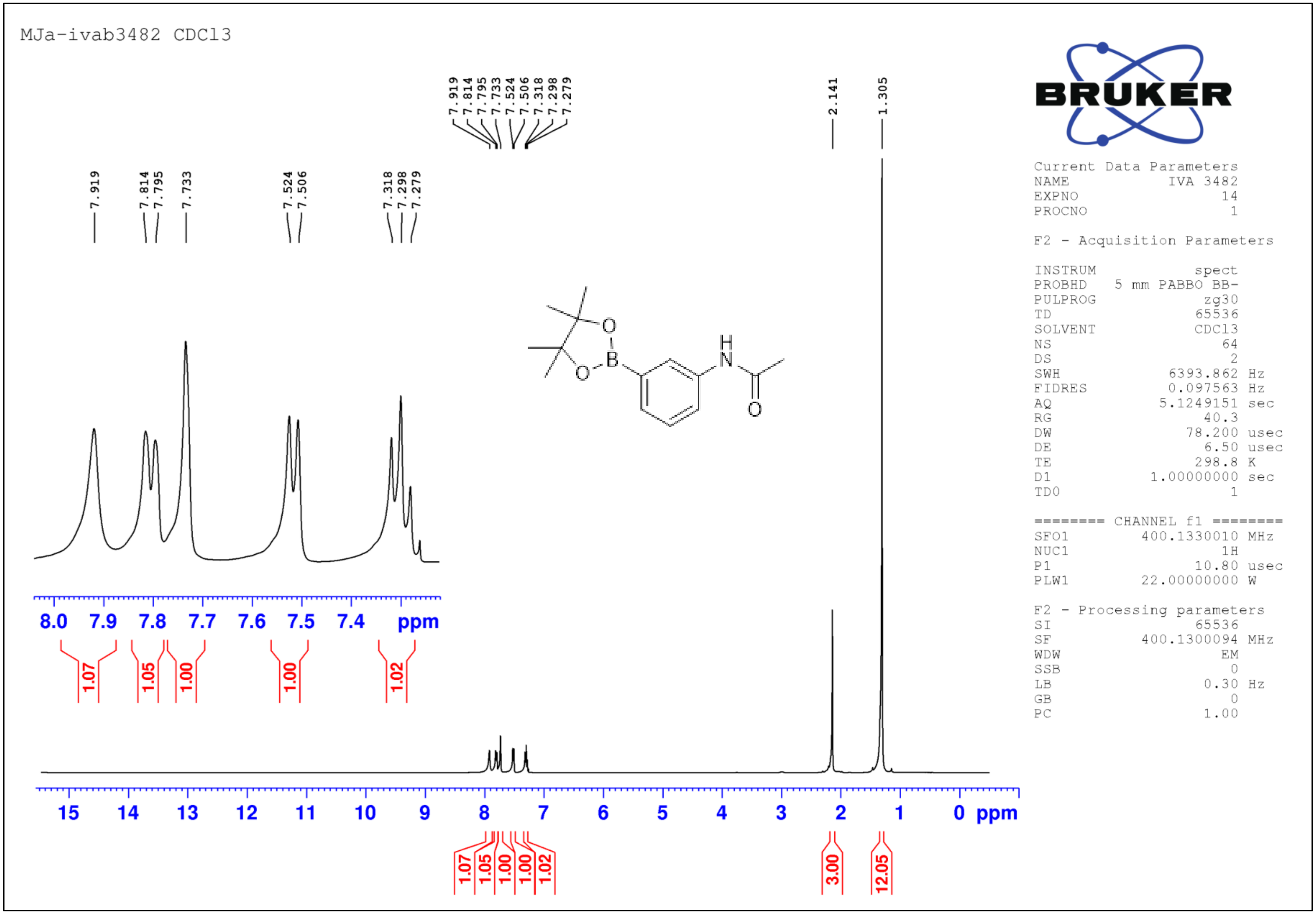

^{1}H NMR Spectrum of Compound 2d

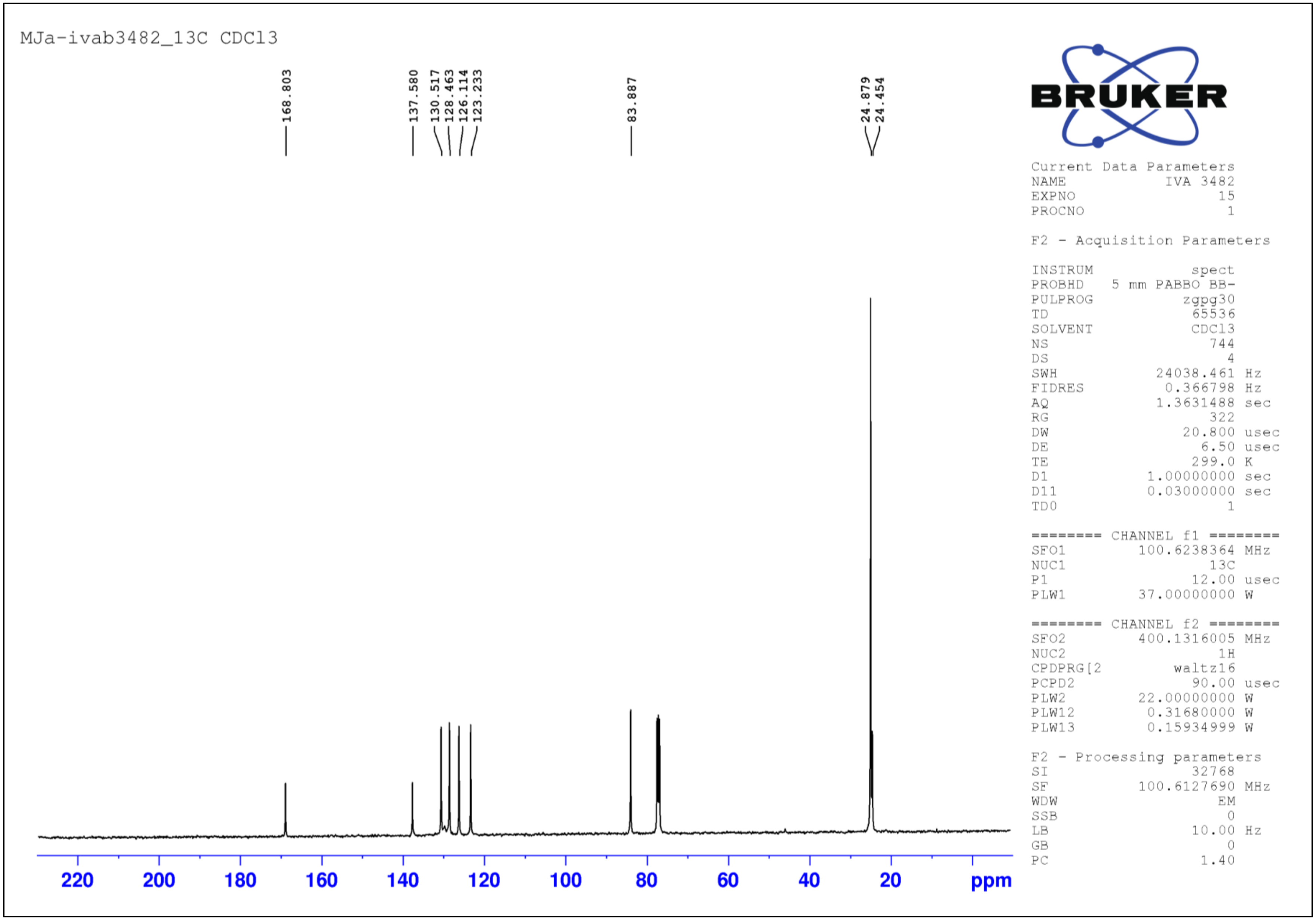

^{13}C NMR Spectrum of Compound 2d

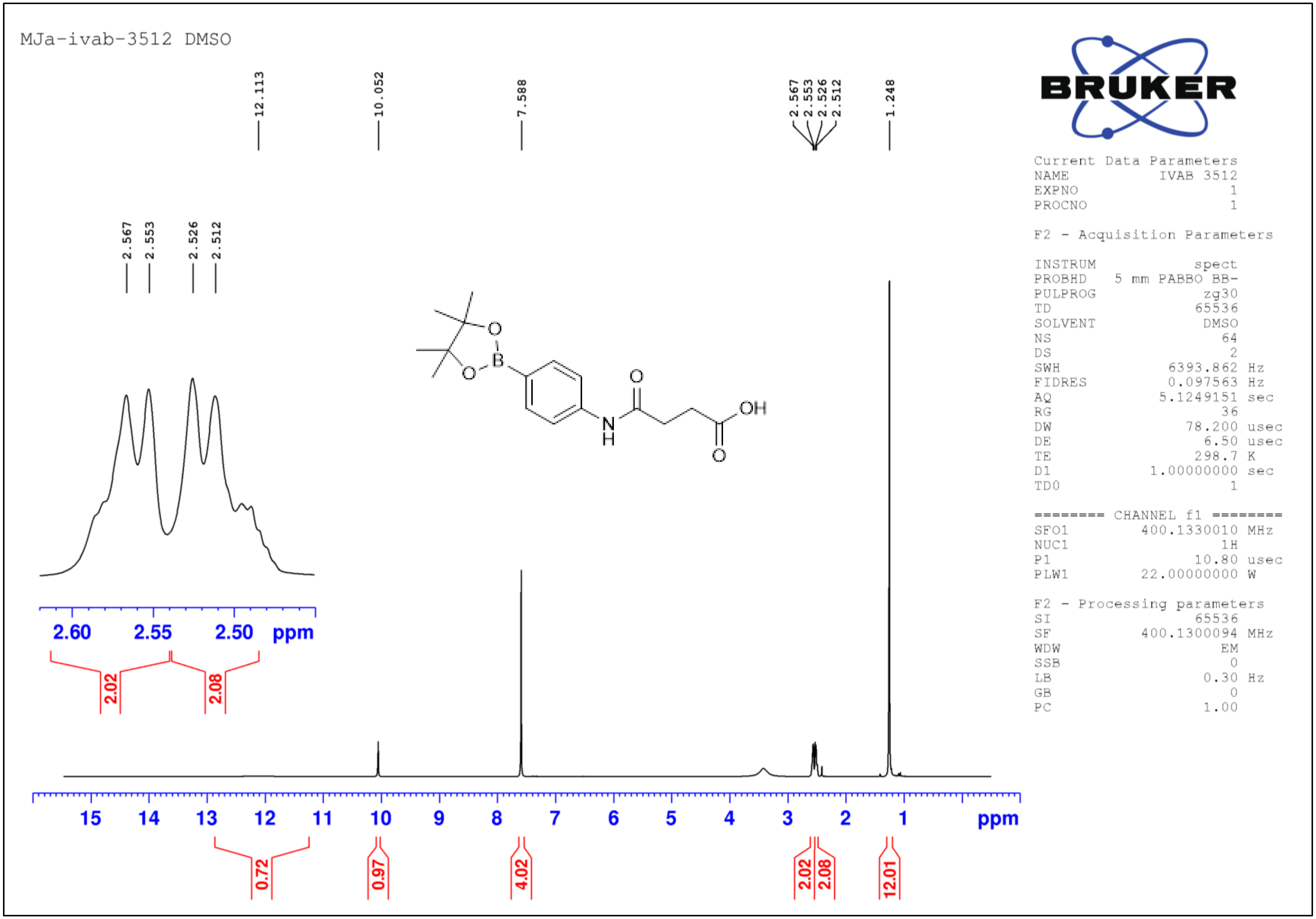

^{1}H NMR Spectrum of Compound 2e

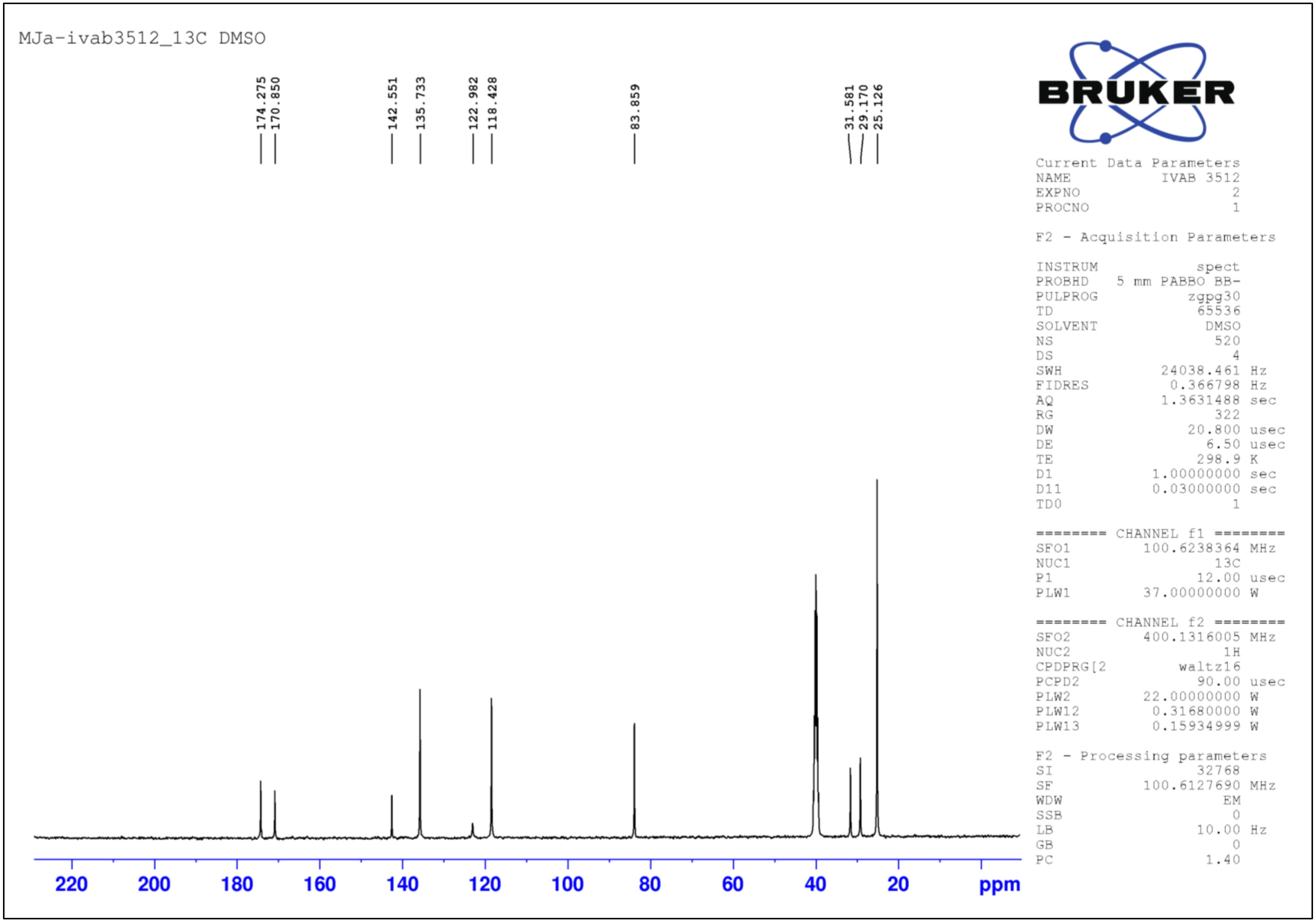

^{13}C NMR Spectrum of Compound 2e

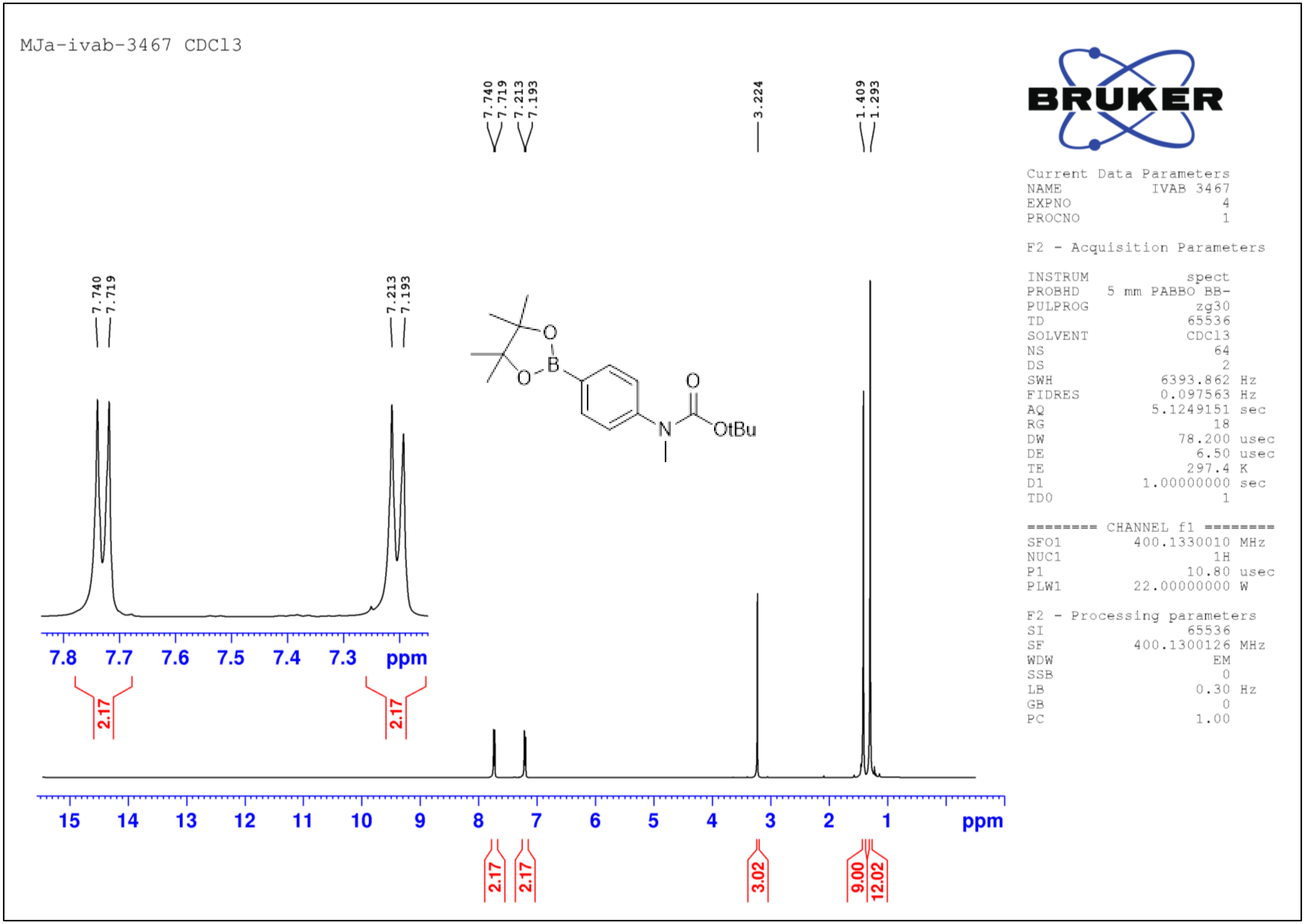

^{1}H NMR Spectrum of Compound 2f

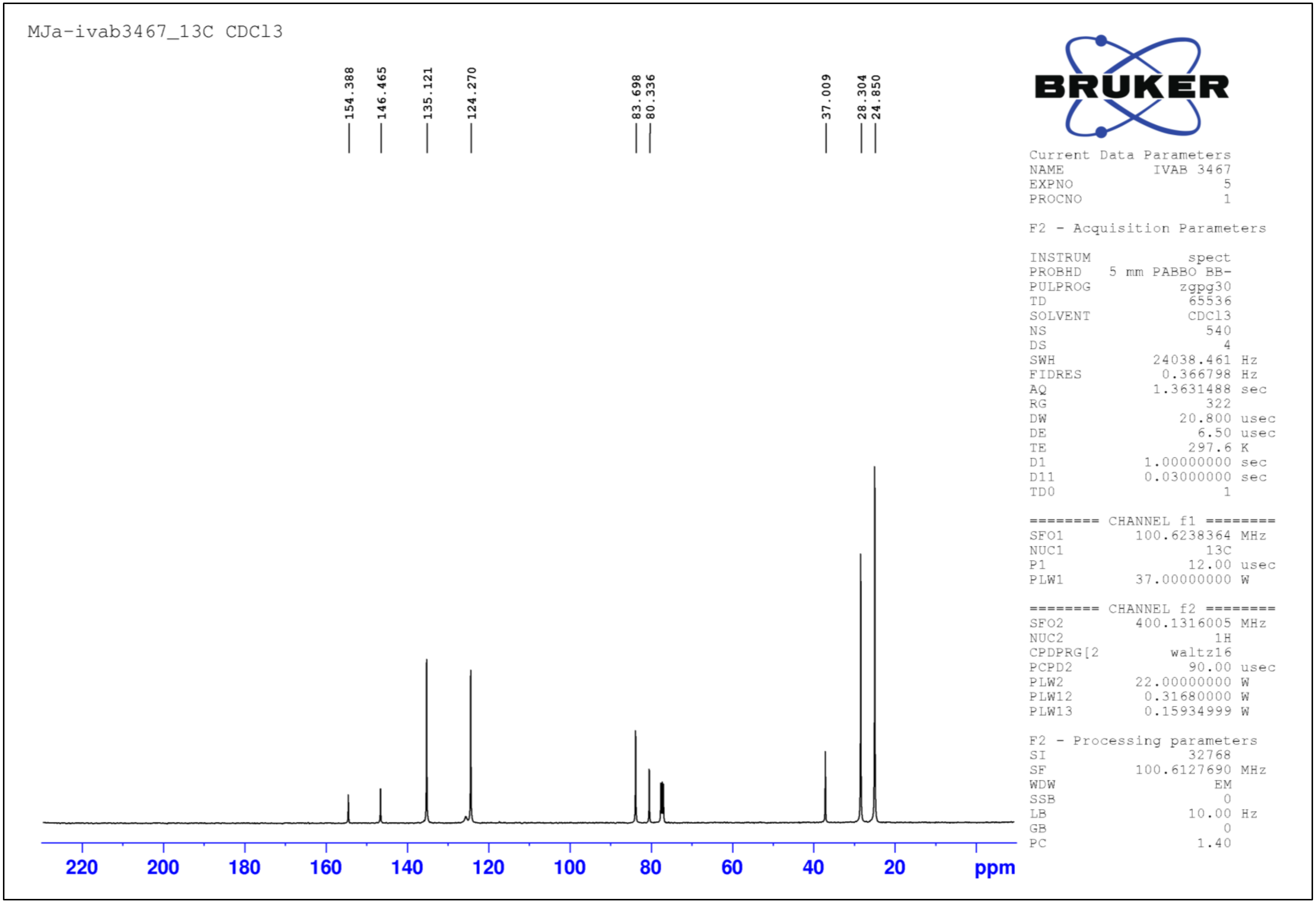

^{13}C NMR Spectrum of Compound 2f

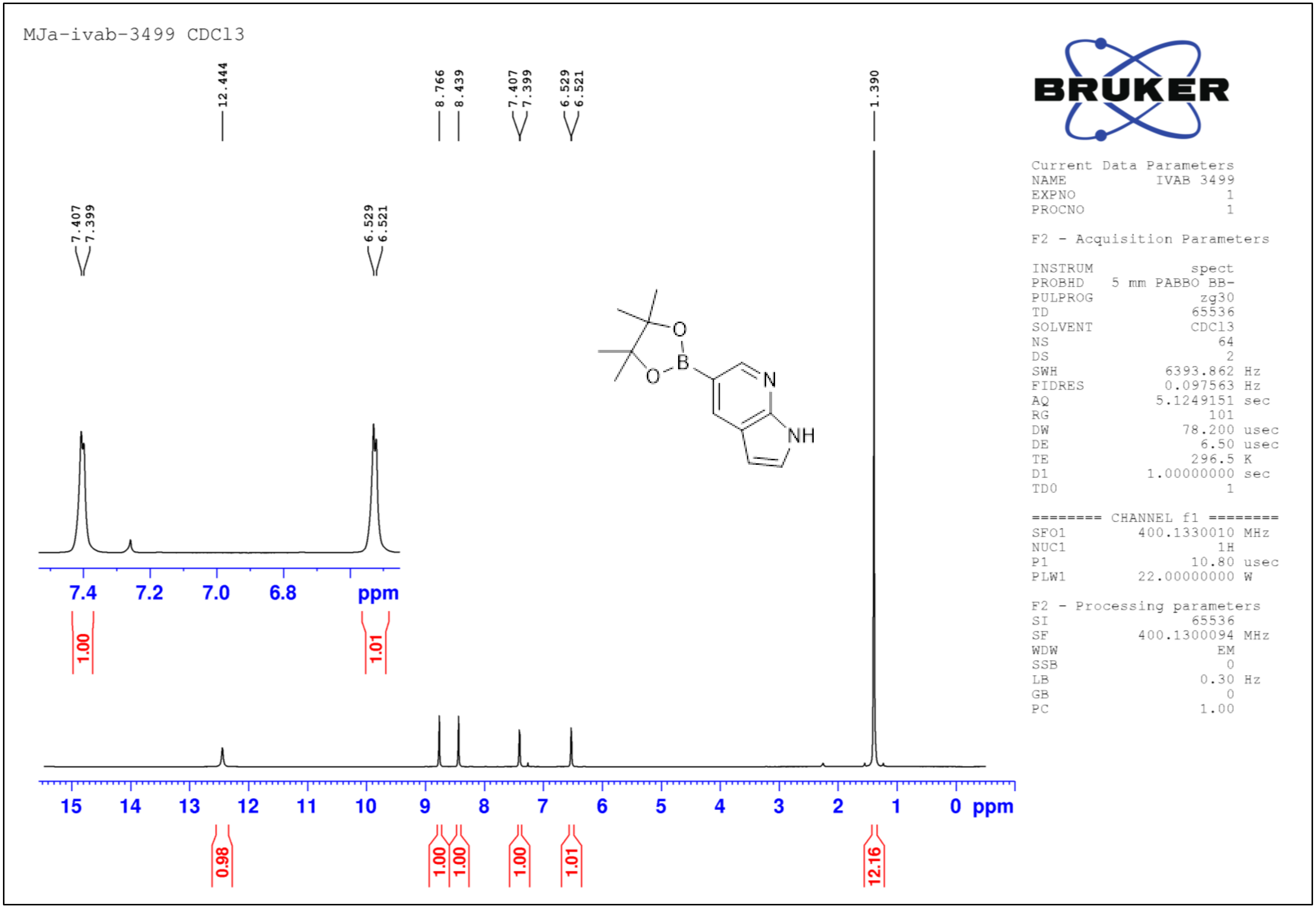

^{1}H NMR Spectrum of Compound 2g

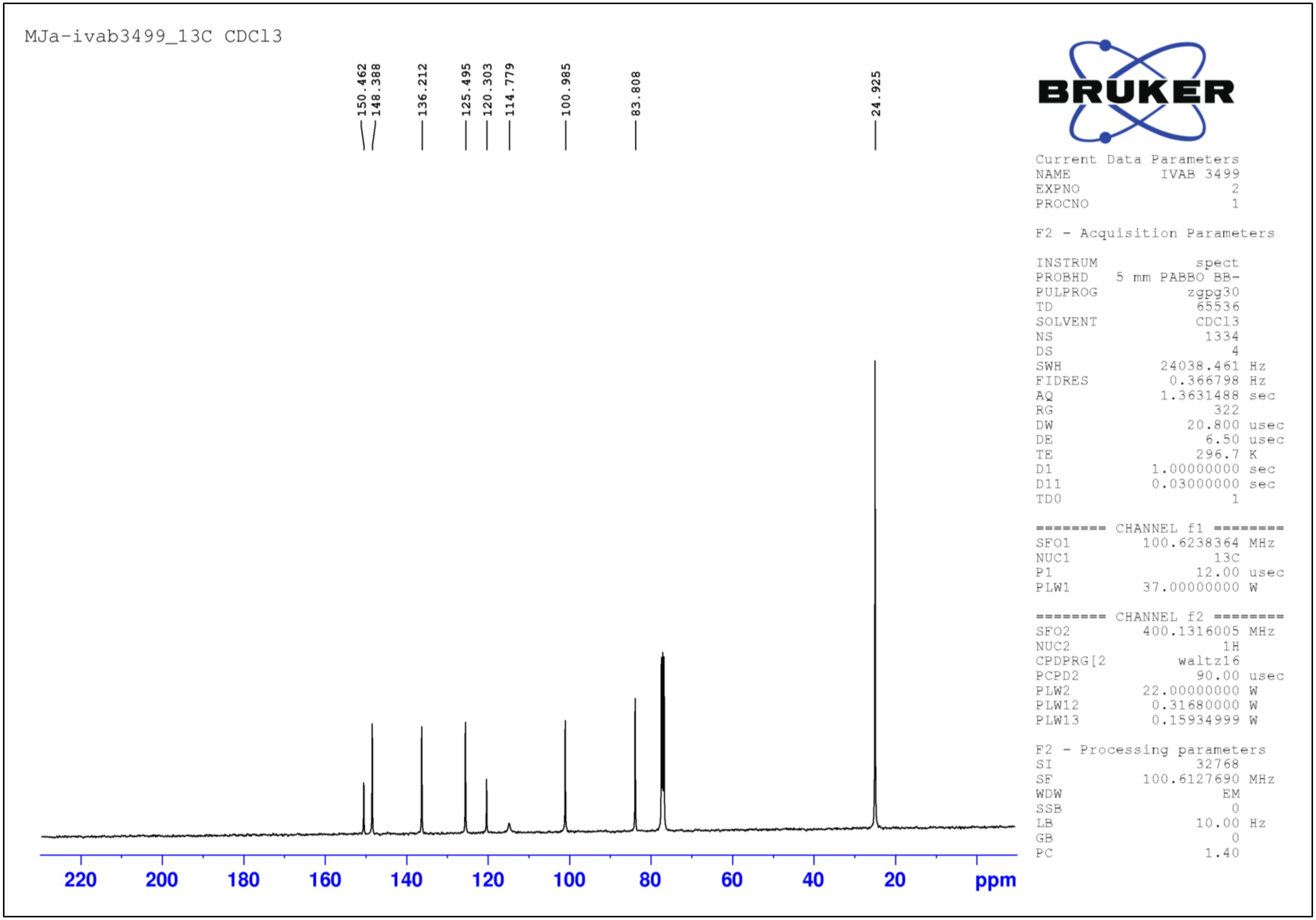

^{13}C NMR Spectrum of Compound 2g

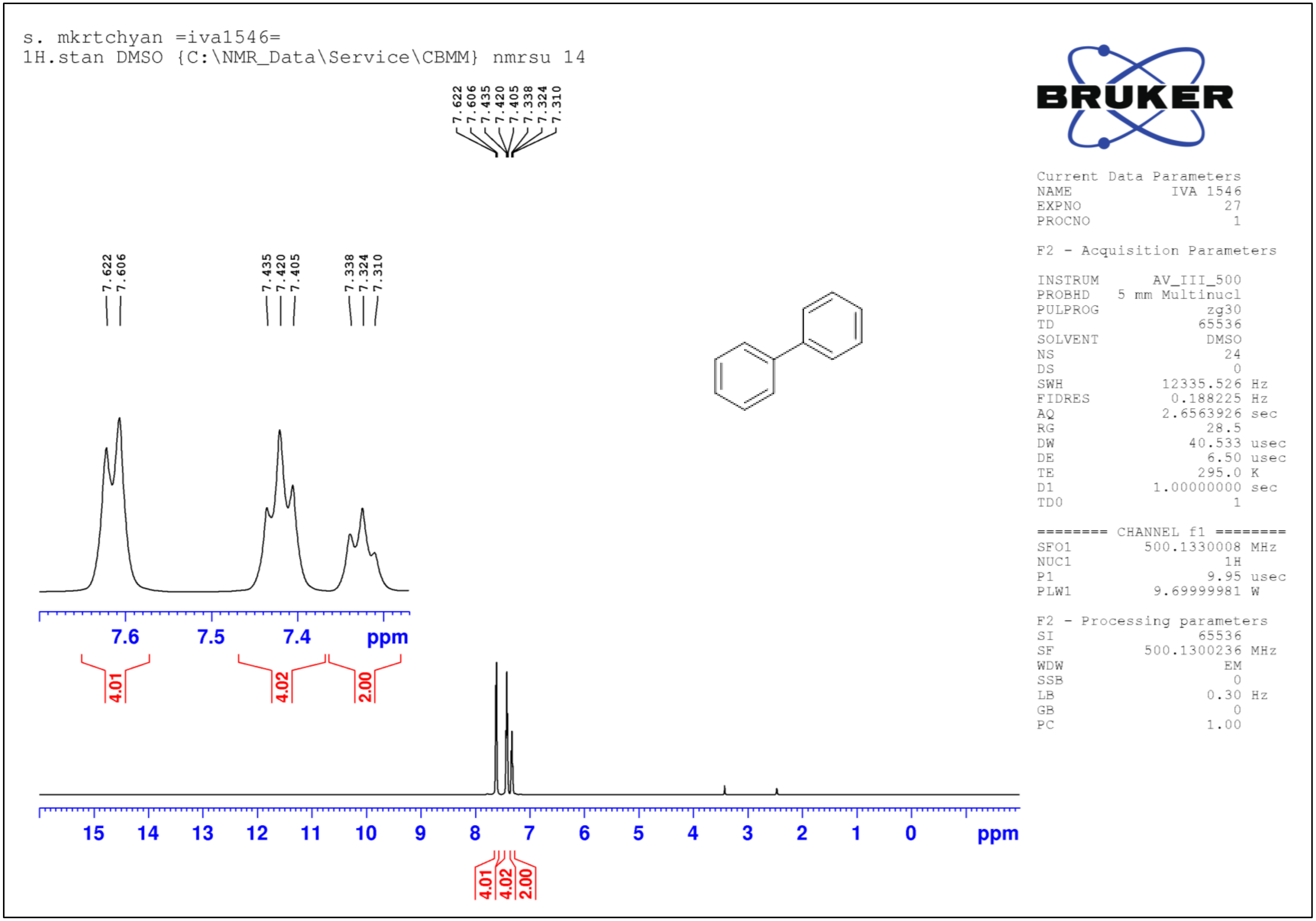

^{1}H NMR Spectrum of Compound 4a

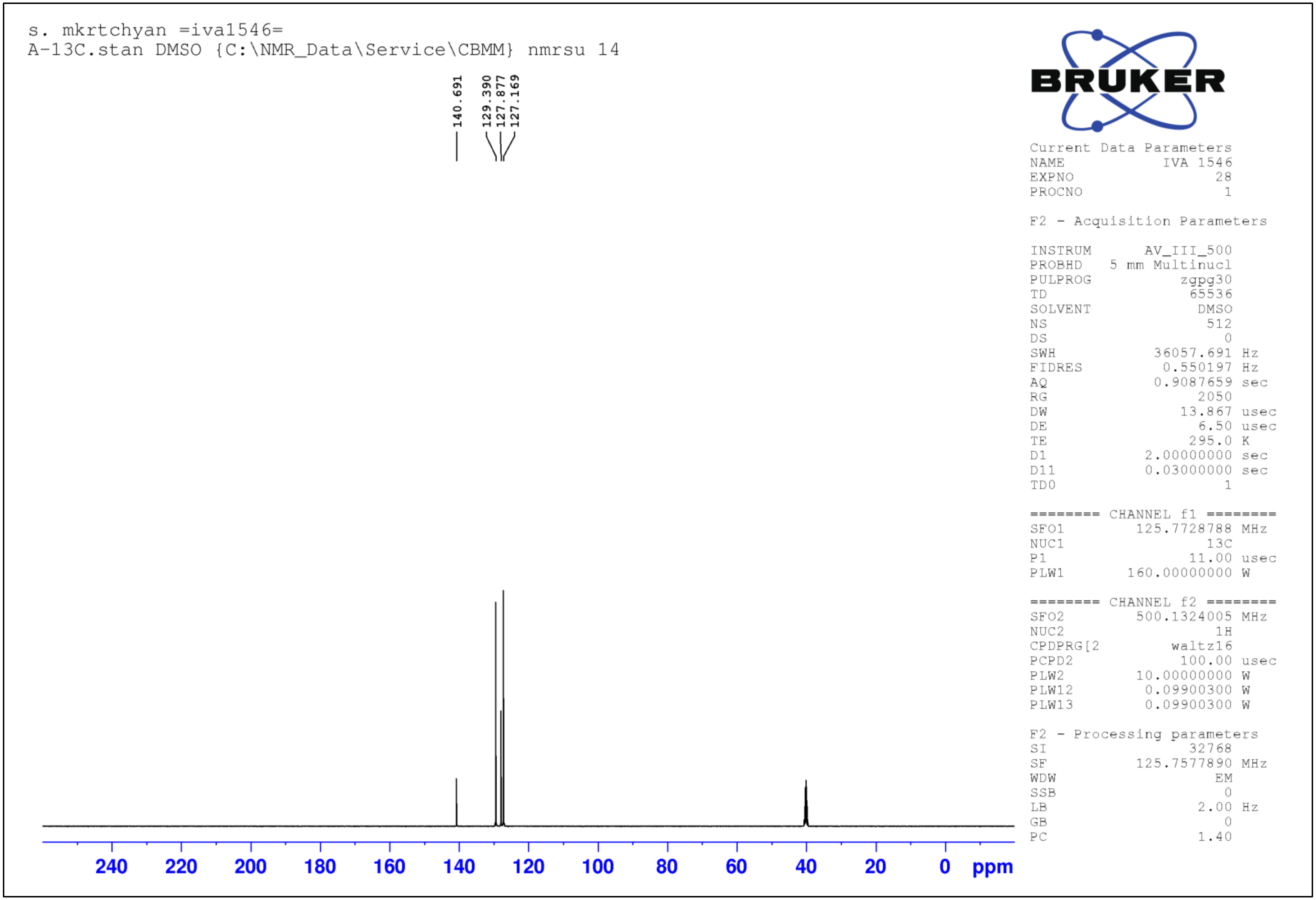

^{13}C NMR Spectrum of Compound 4a

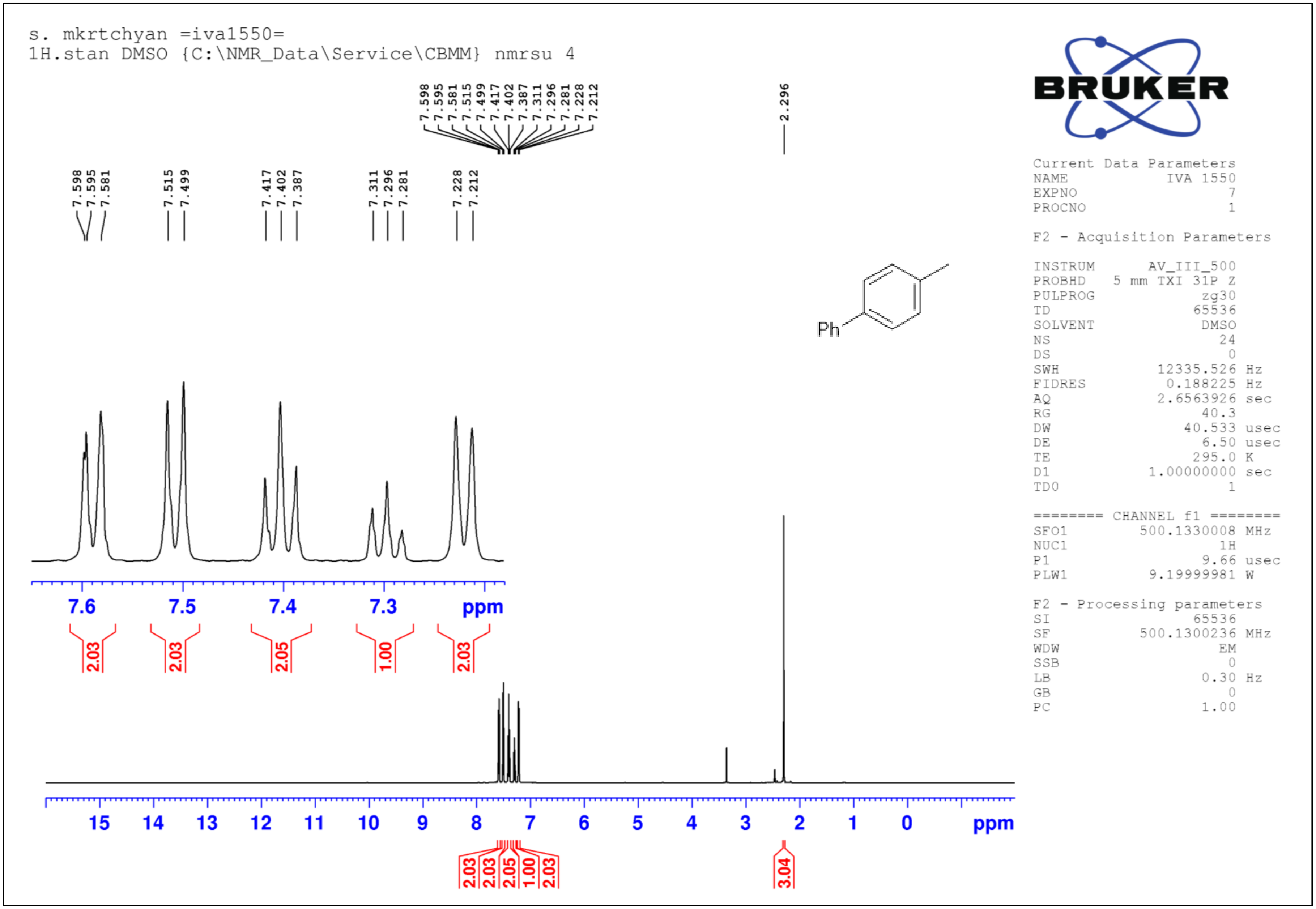

^{1}H NMR Spectrum of Compound 4b

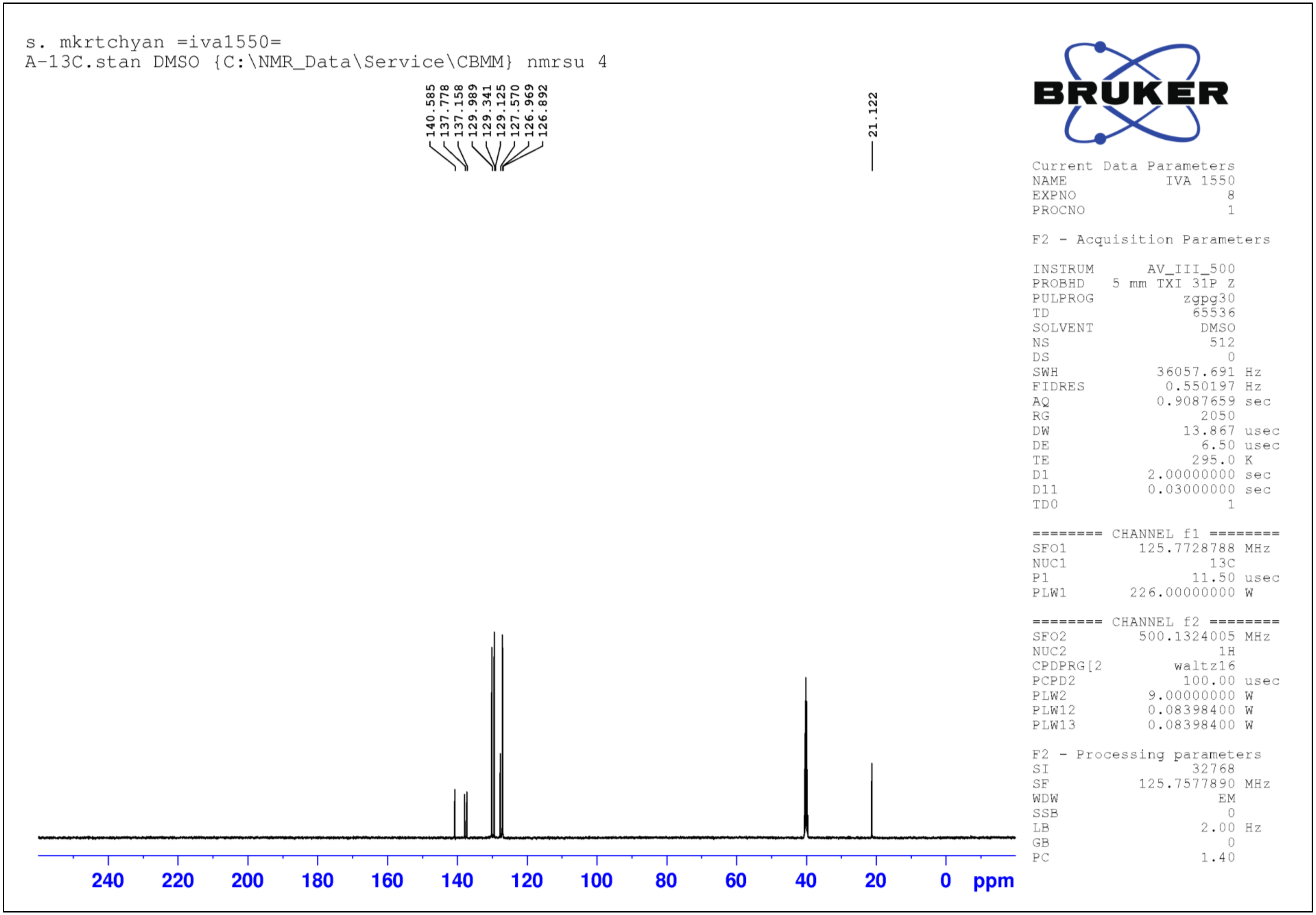

^{13}C NMR Spectrum of Compound 4b

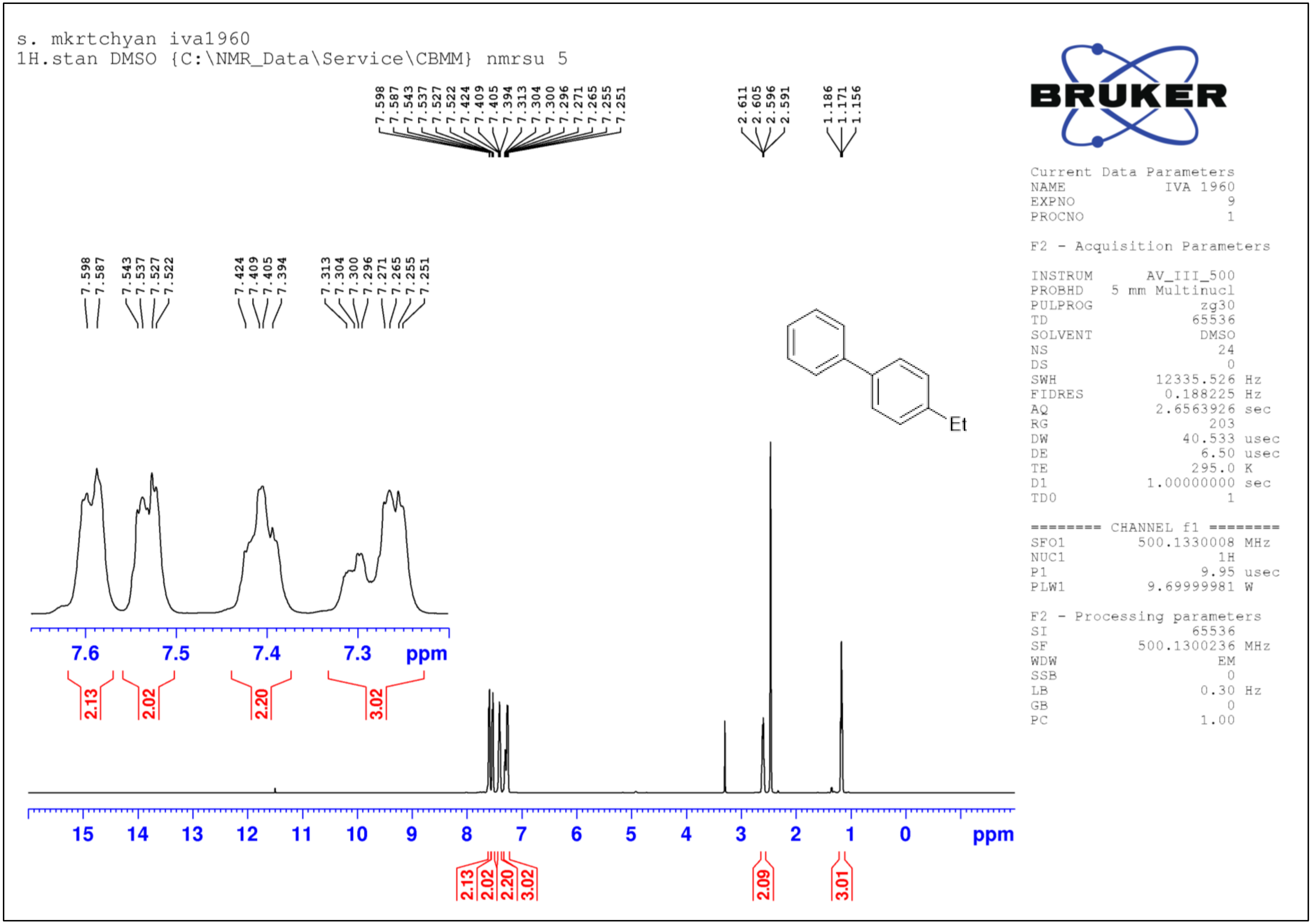

^{1}H NMR Spectrum of Compound 4c

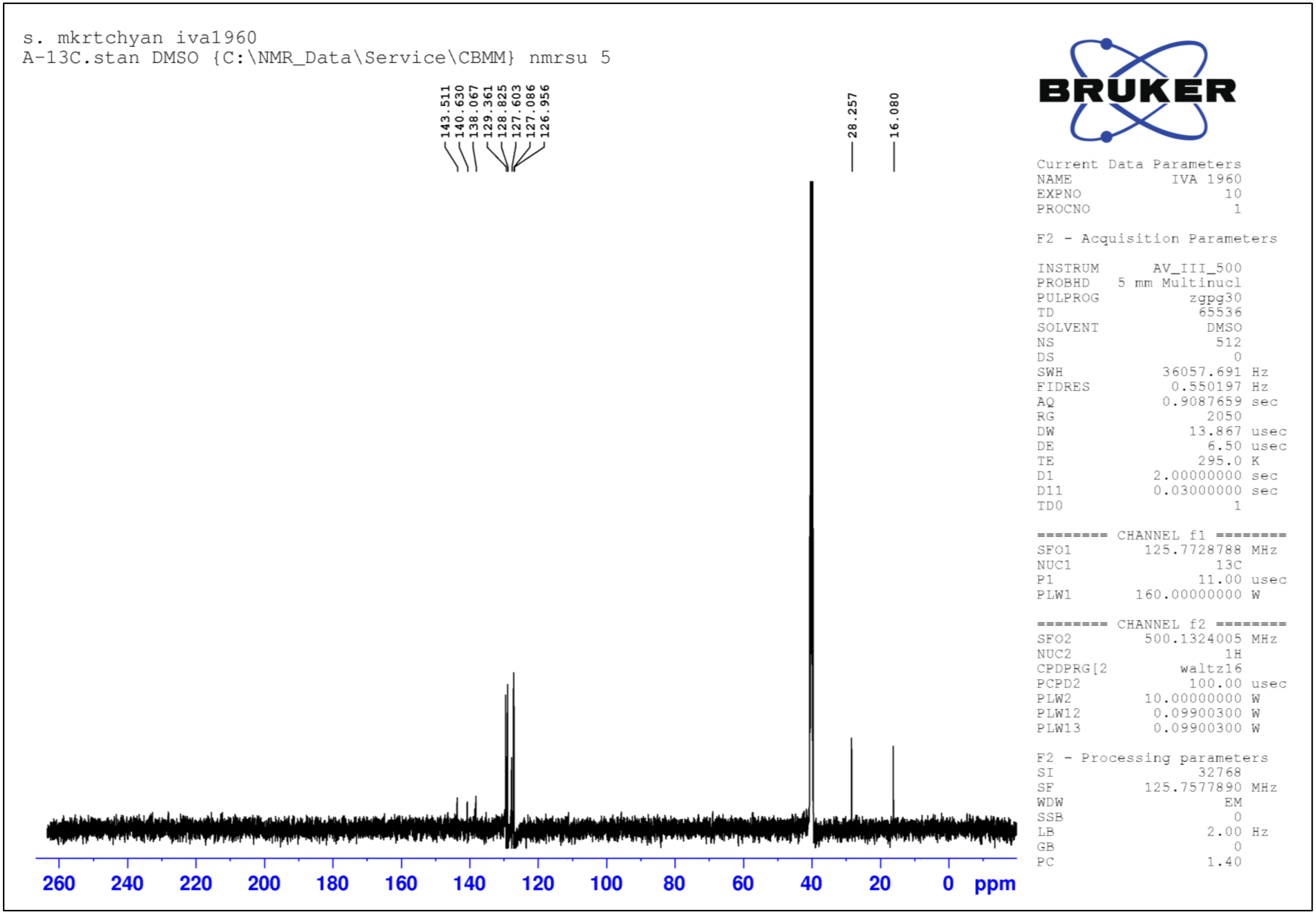

^{13}C NMR Spectrum of Compound 4c

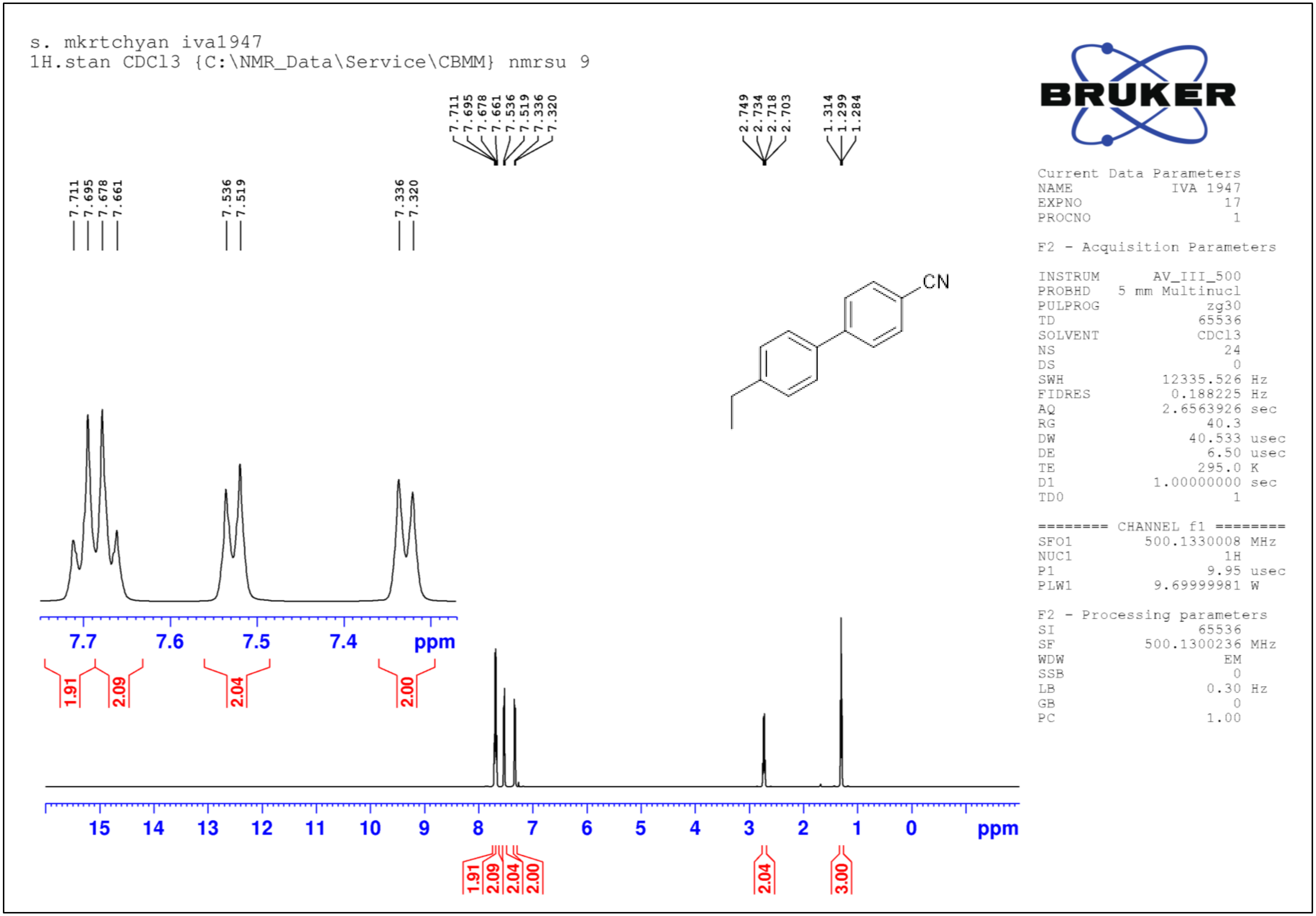

^{1}H NMR Spectrum of Compound 4d

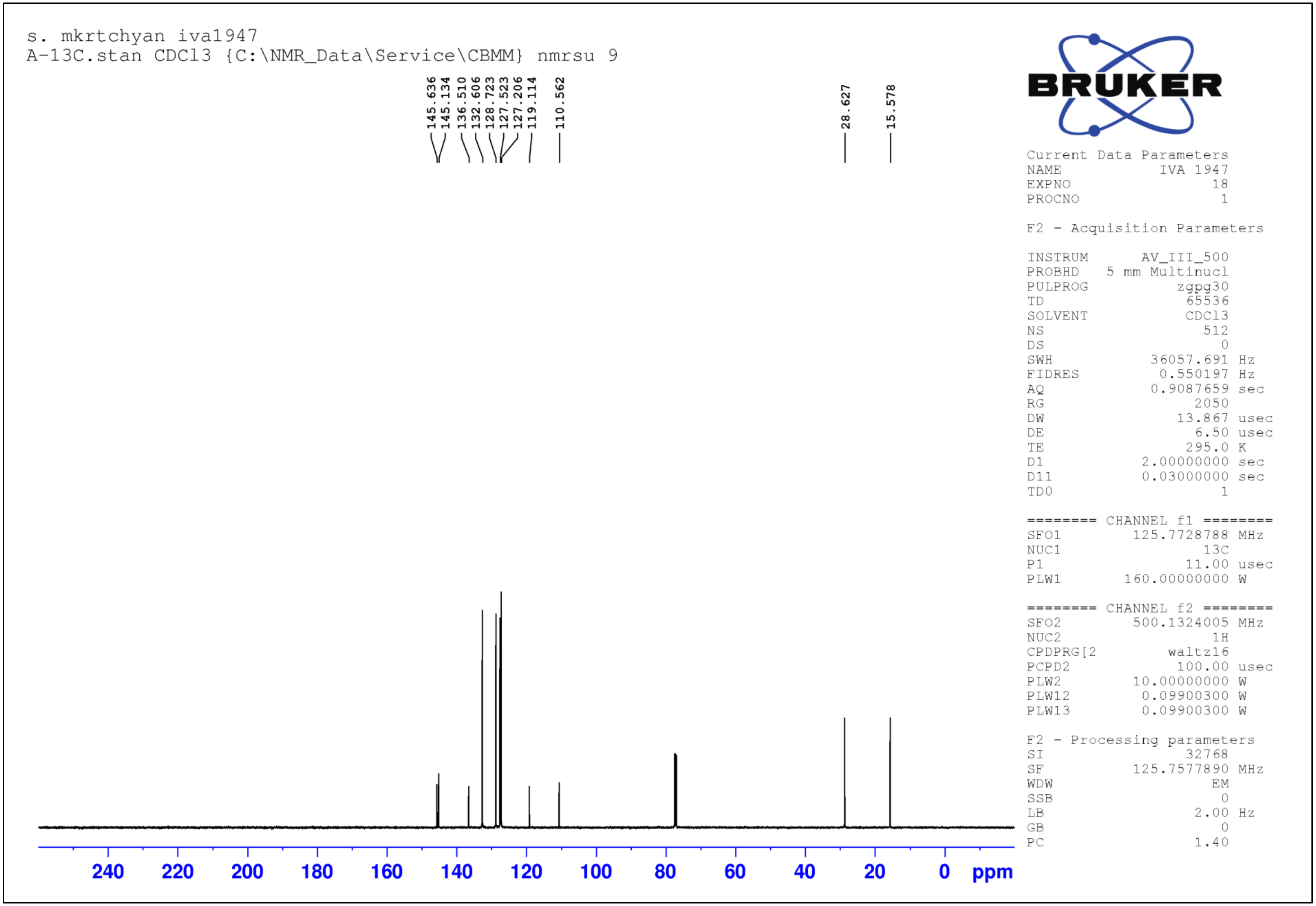

^{13}C NMR Spectrum of Compound 4d

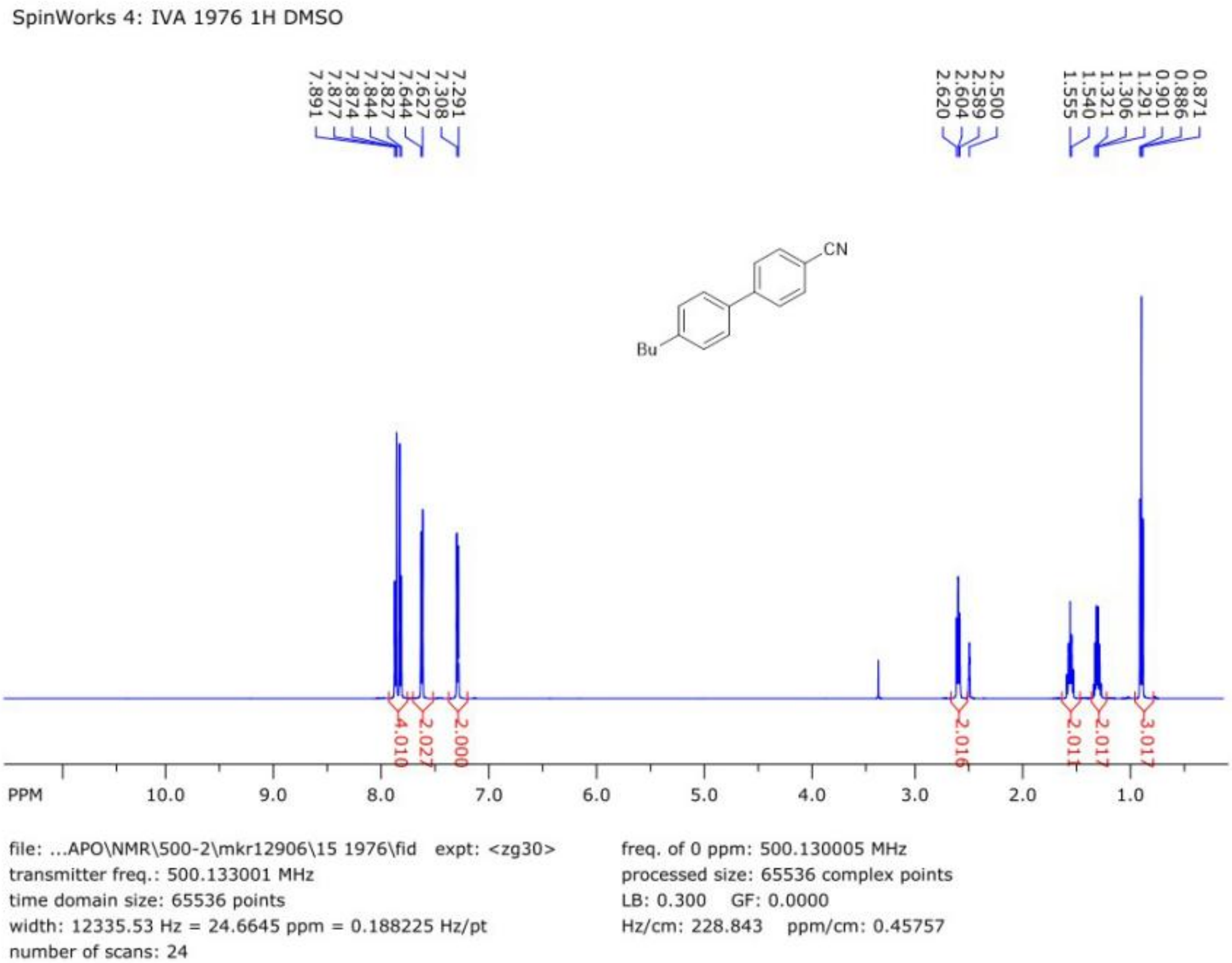

^{1}H NMR Spectrum of Compound 4e

SpinWorks 4: IVA 1976 13C DMSO

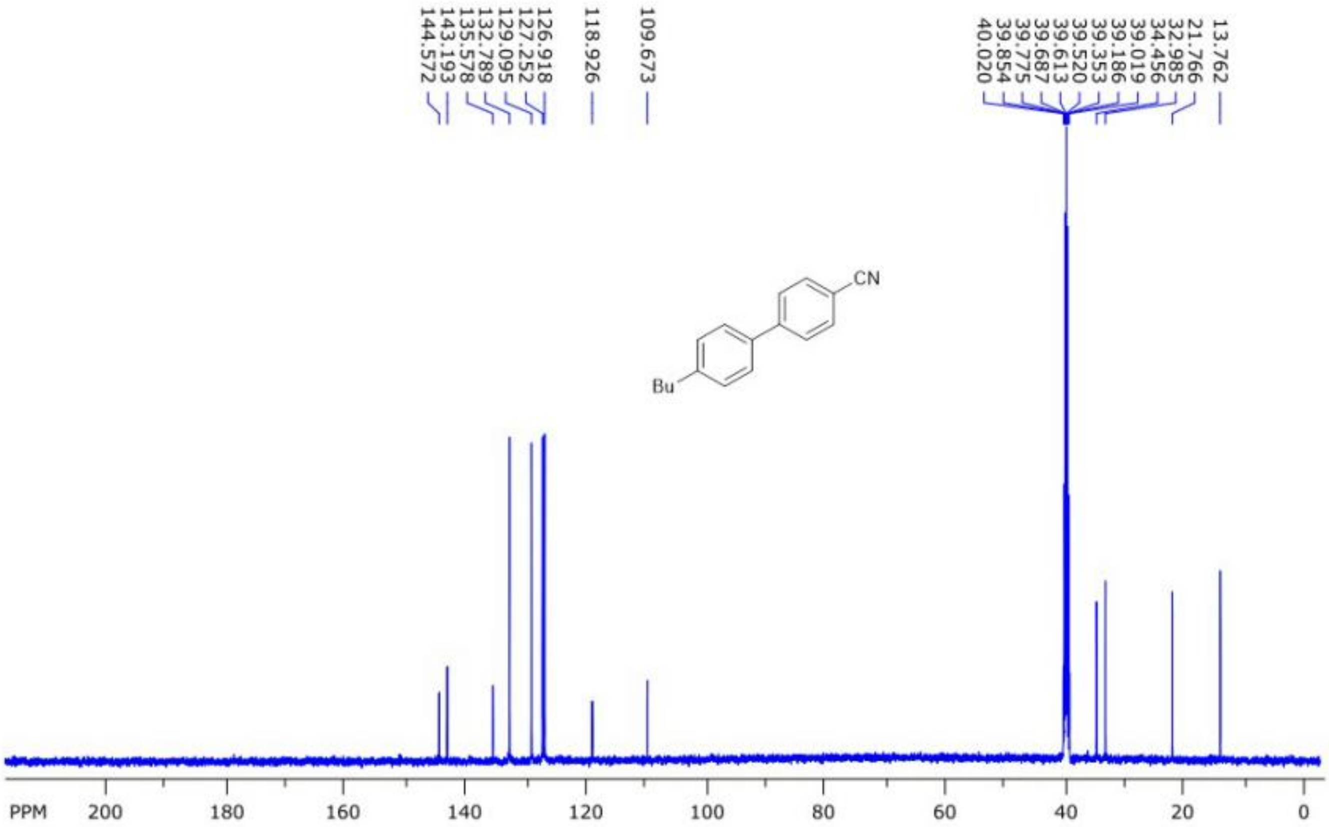

file: D:\NAPO\NMR\500-2\mkr12906\16\fid expt: <zgpg30>
transmitter freq.: 125.772879 MHz
time domain size: 65536 points
width: 36057.69 Hz = 286.6889 ppm = 0.550197 Hz/pt
number of scans: 512

freq. of 0 ppm: 125.757845 MHz
processed size: 32768 complex points
LB: 2.000 GF: 0.0000
Hz/cm: 1107.258 ppm/cm: 8.80363

^{13}C NMR Spectrum of Compound 4e

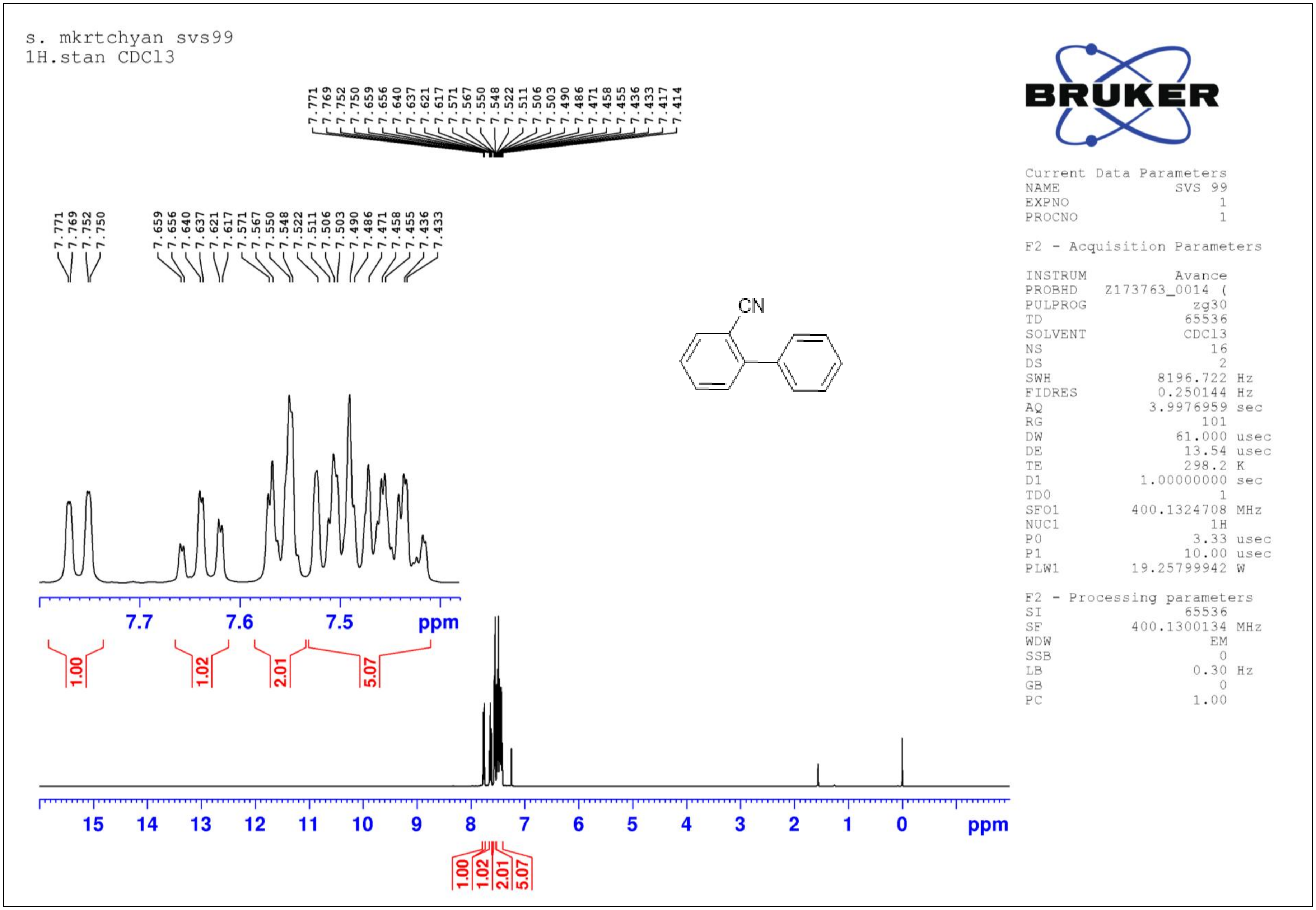

^{1}H NMR Spectrum of Compound 4f

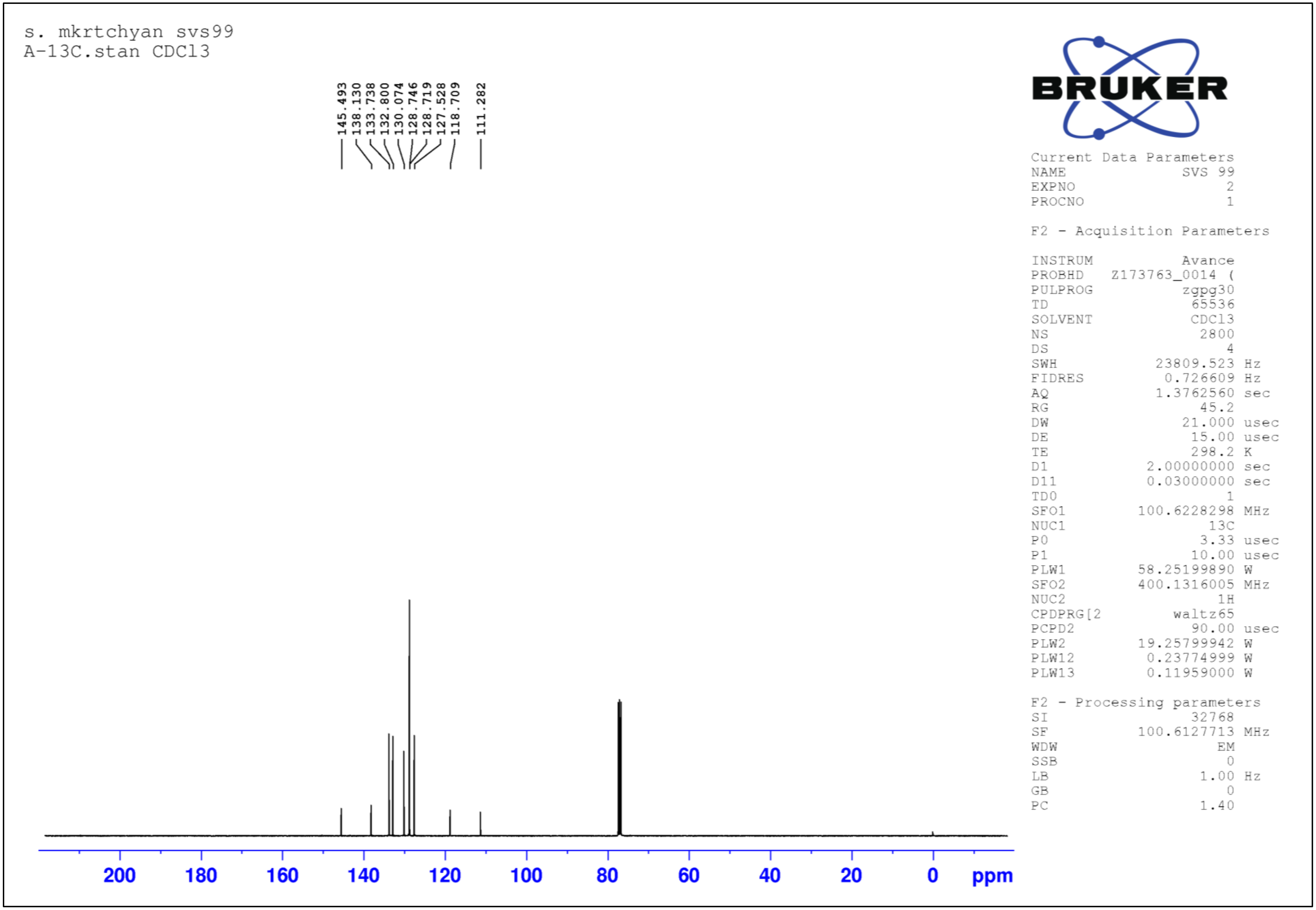

^{13}C NMR Spectrum of Compound 4f

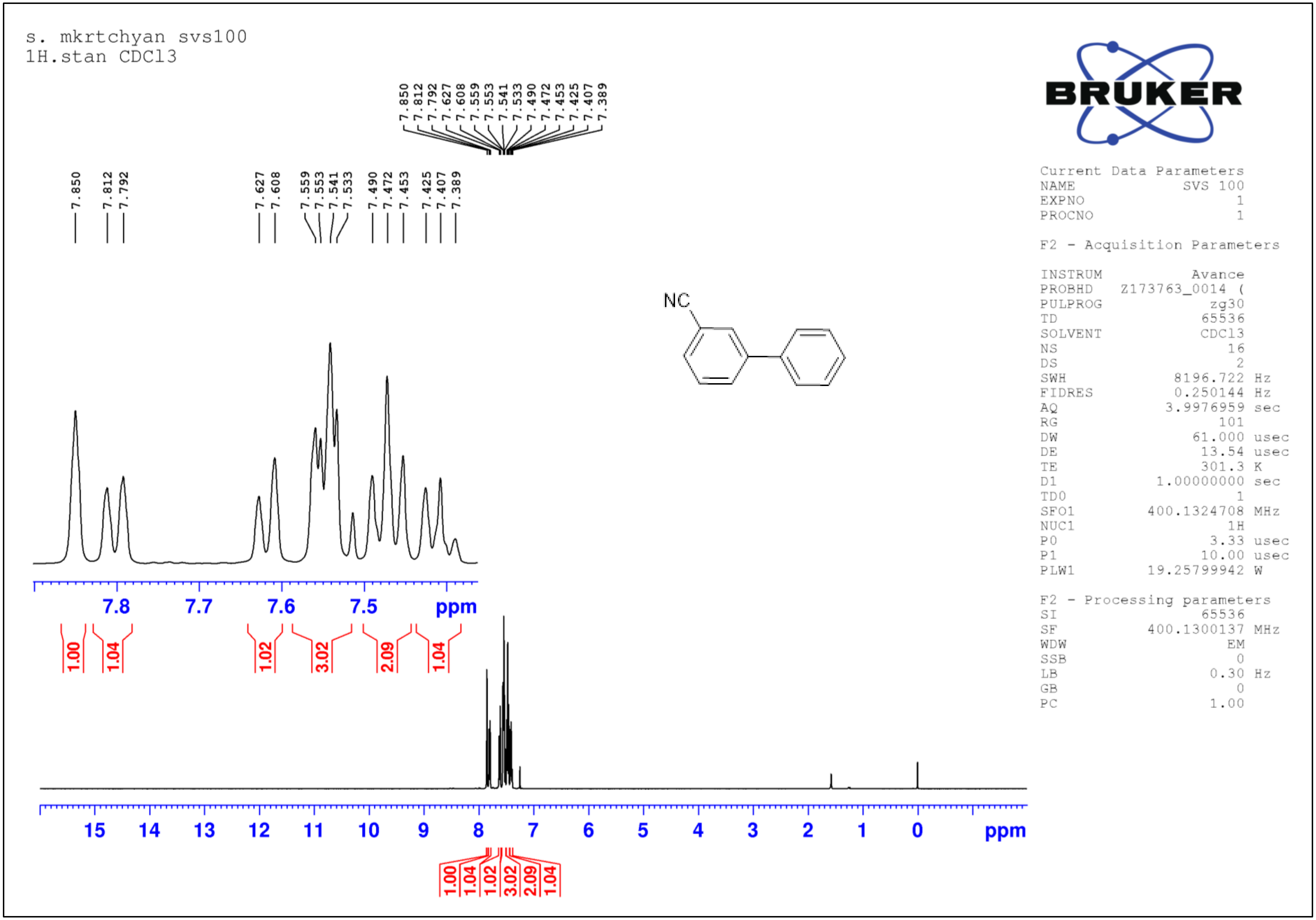

^{1}H NMR Spectrum of Compound 4g

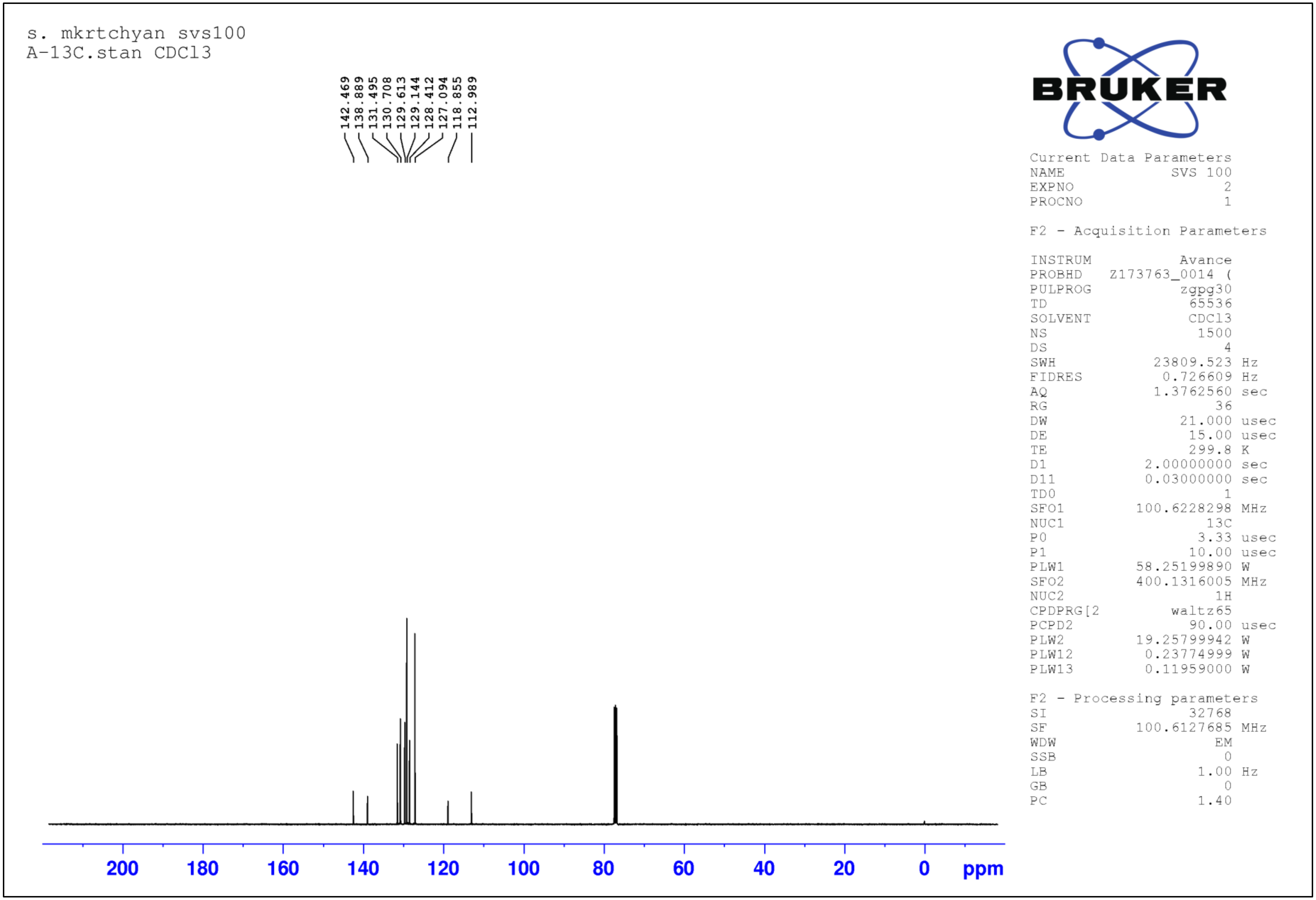

^{13}C NMR Spectrum of Compound 4g

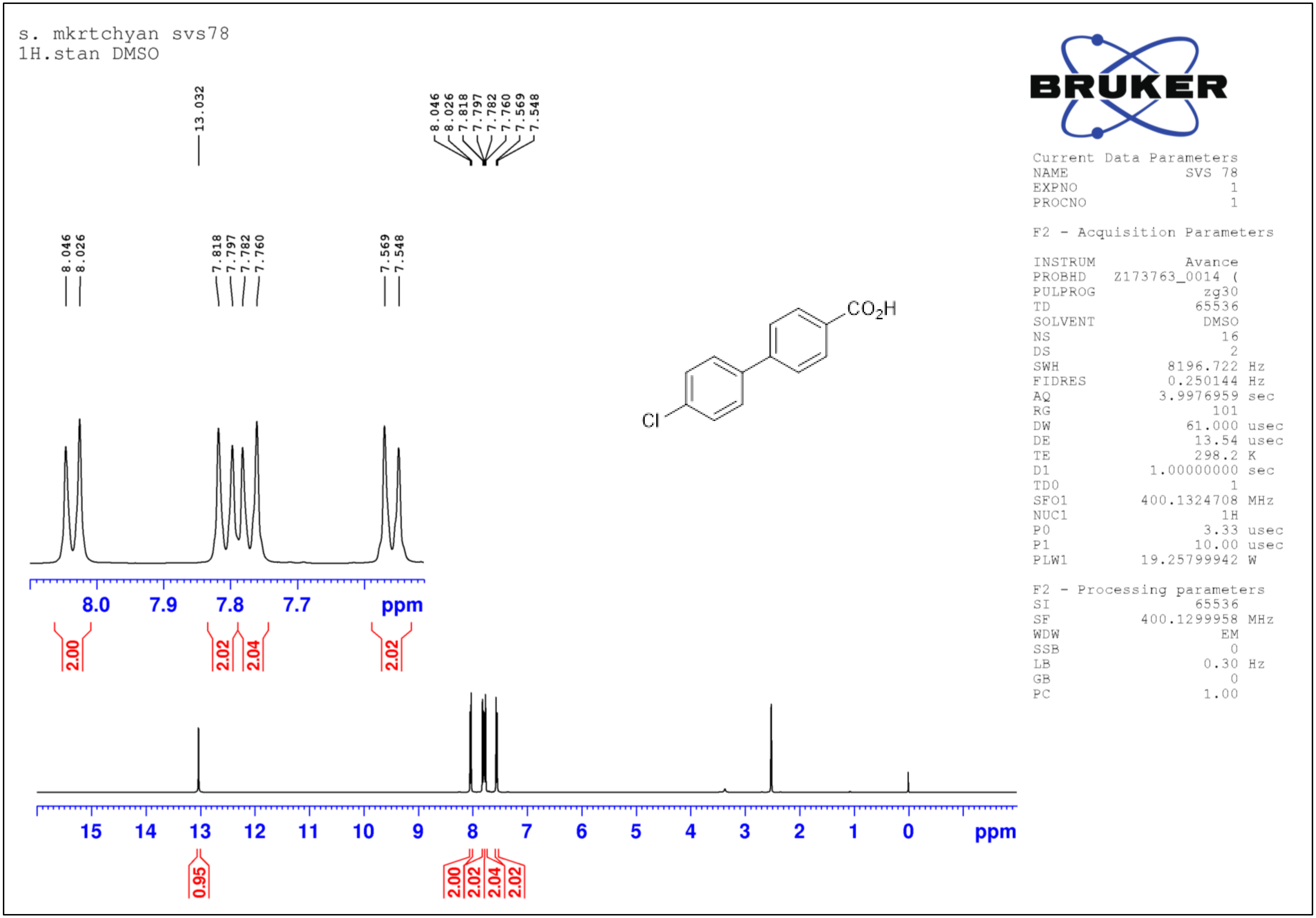

¹H NMR Spectrum of Compound 4h

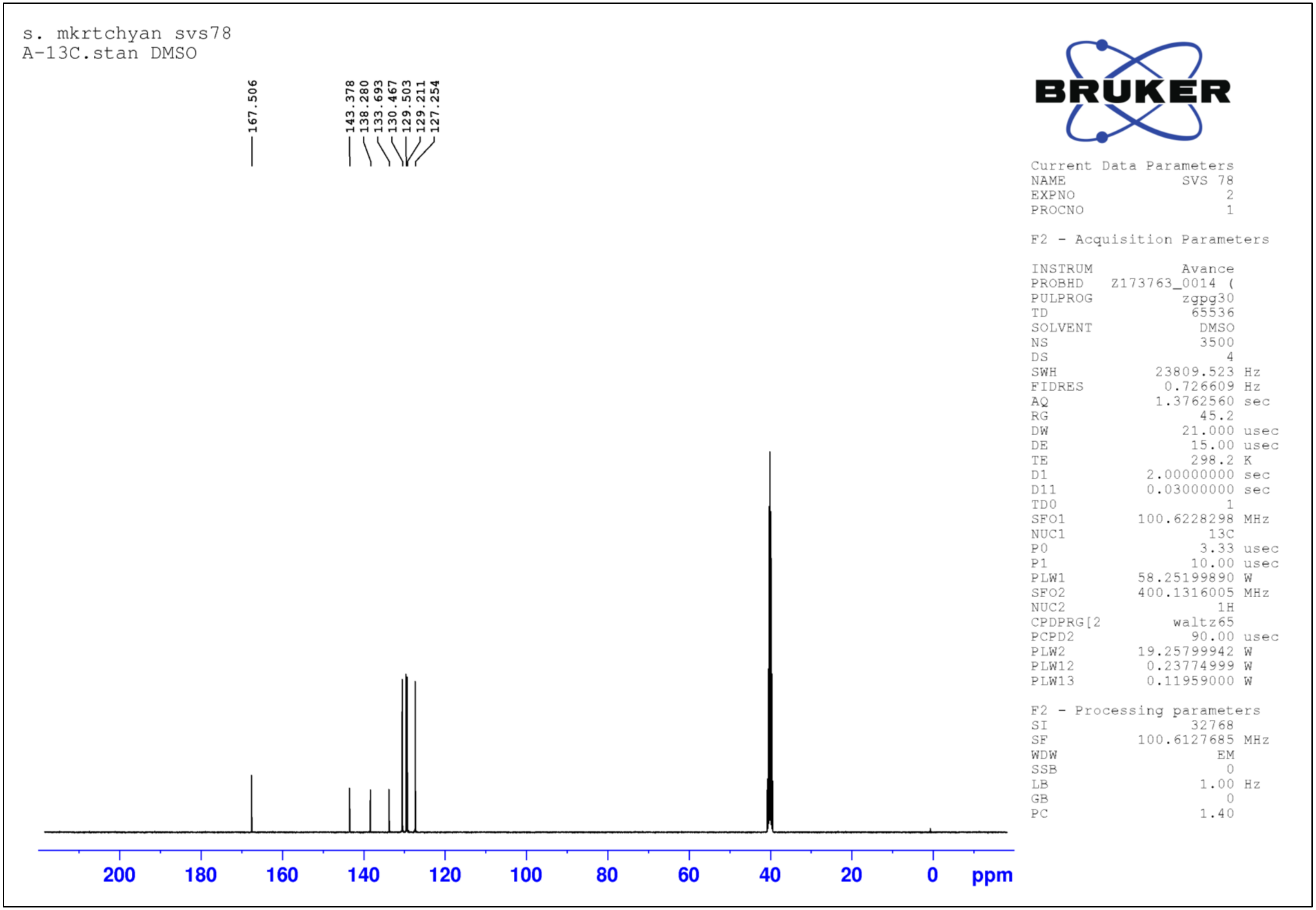

^{13}C NMR Spectrum of Compound 4h

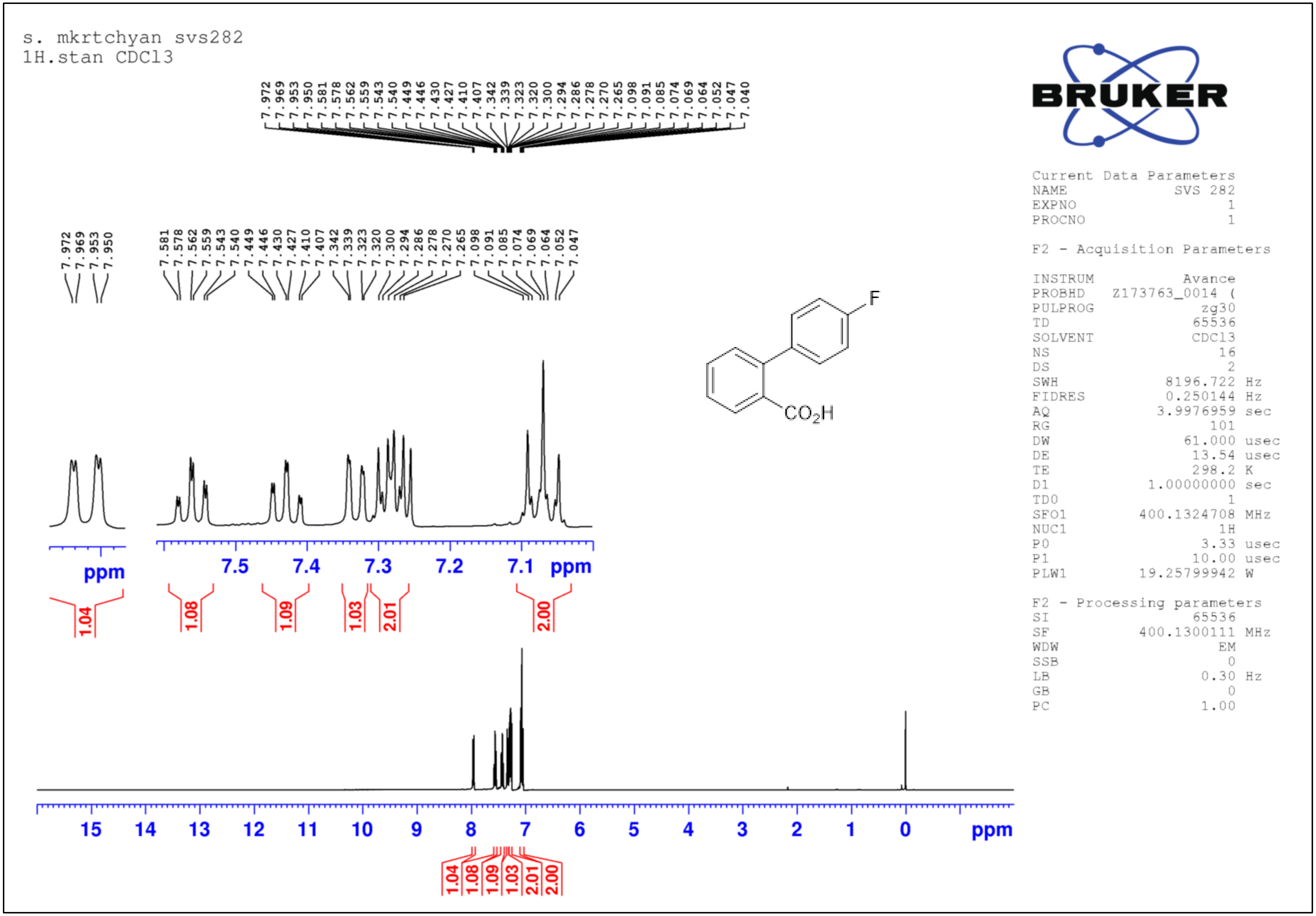

^{1}H NMR Spectrum of Compound 4i

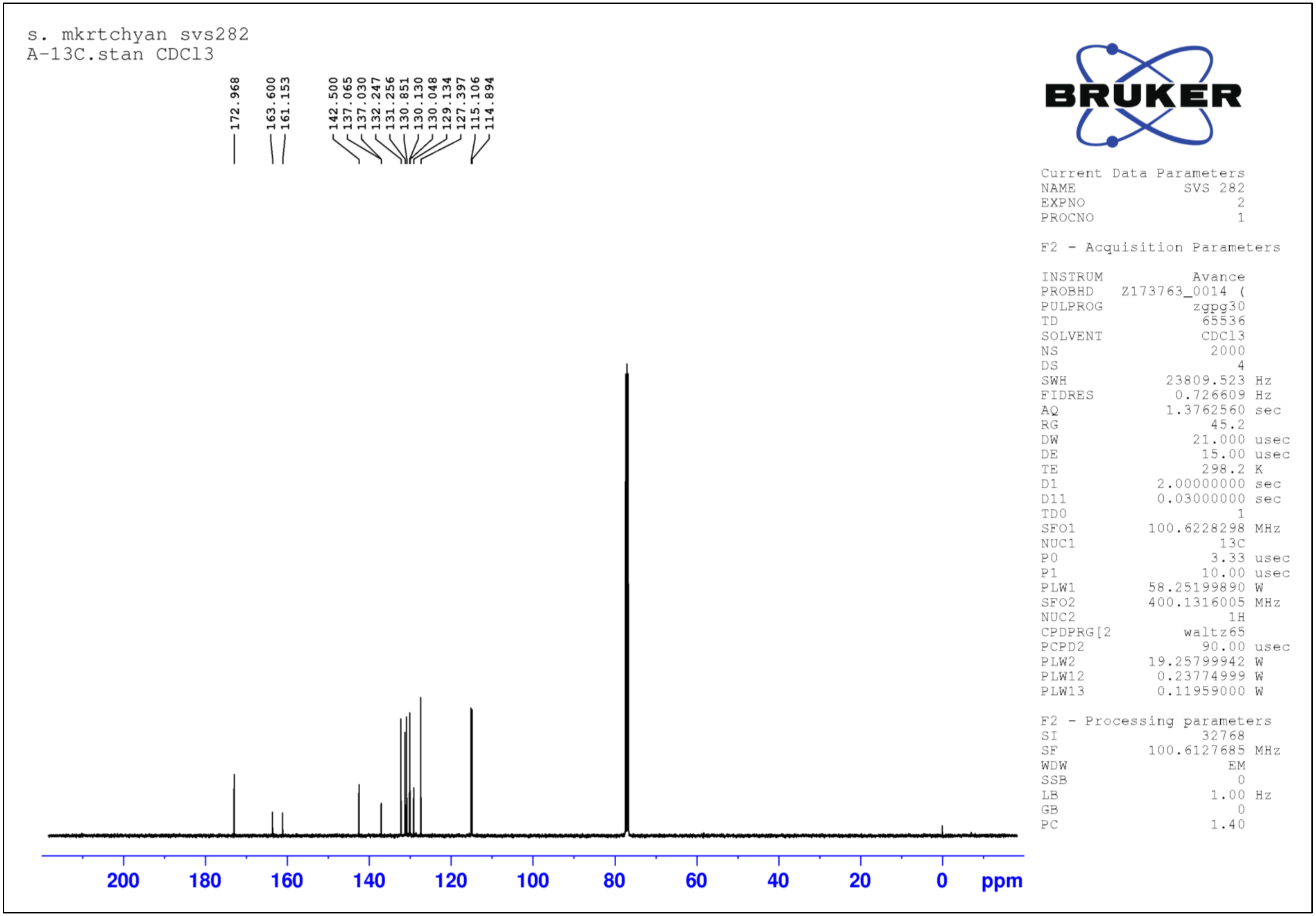

^{13}C NMR Spectrum of Compound 4i

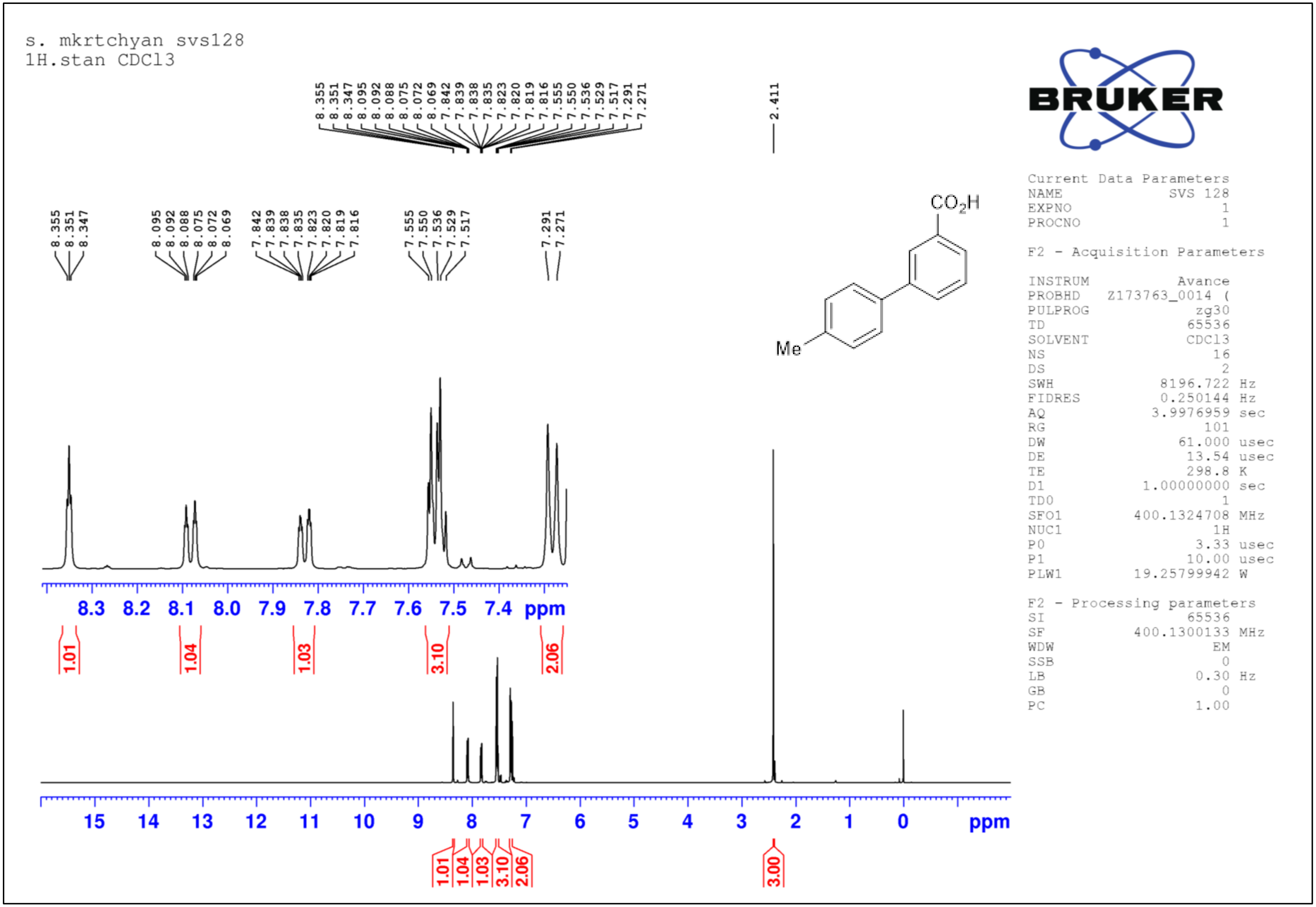

^{1}H NMR Spectrum of Compound 4j

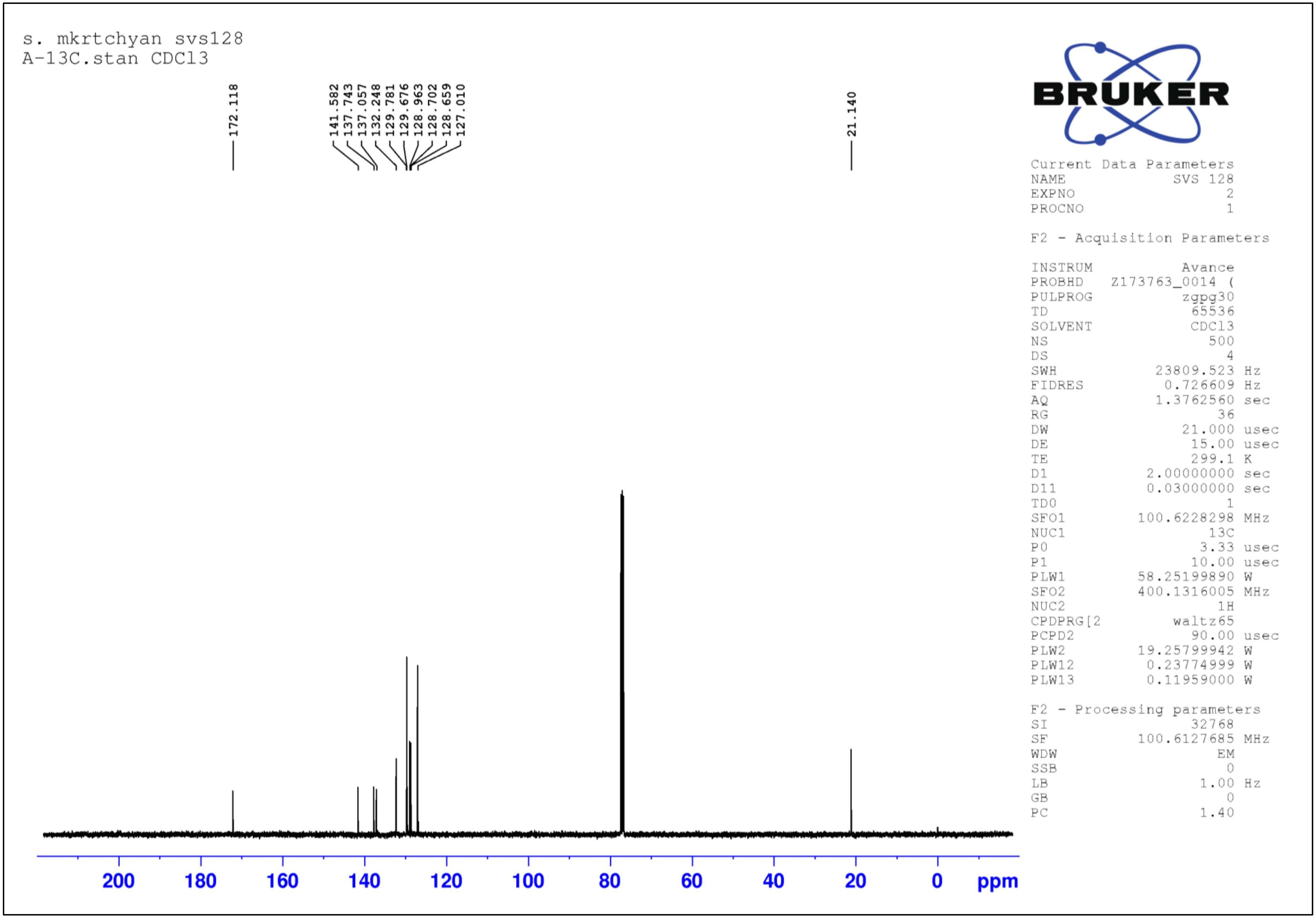

^{13}C NMR Spectrum of Compound 4j

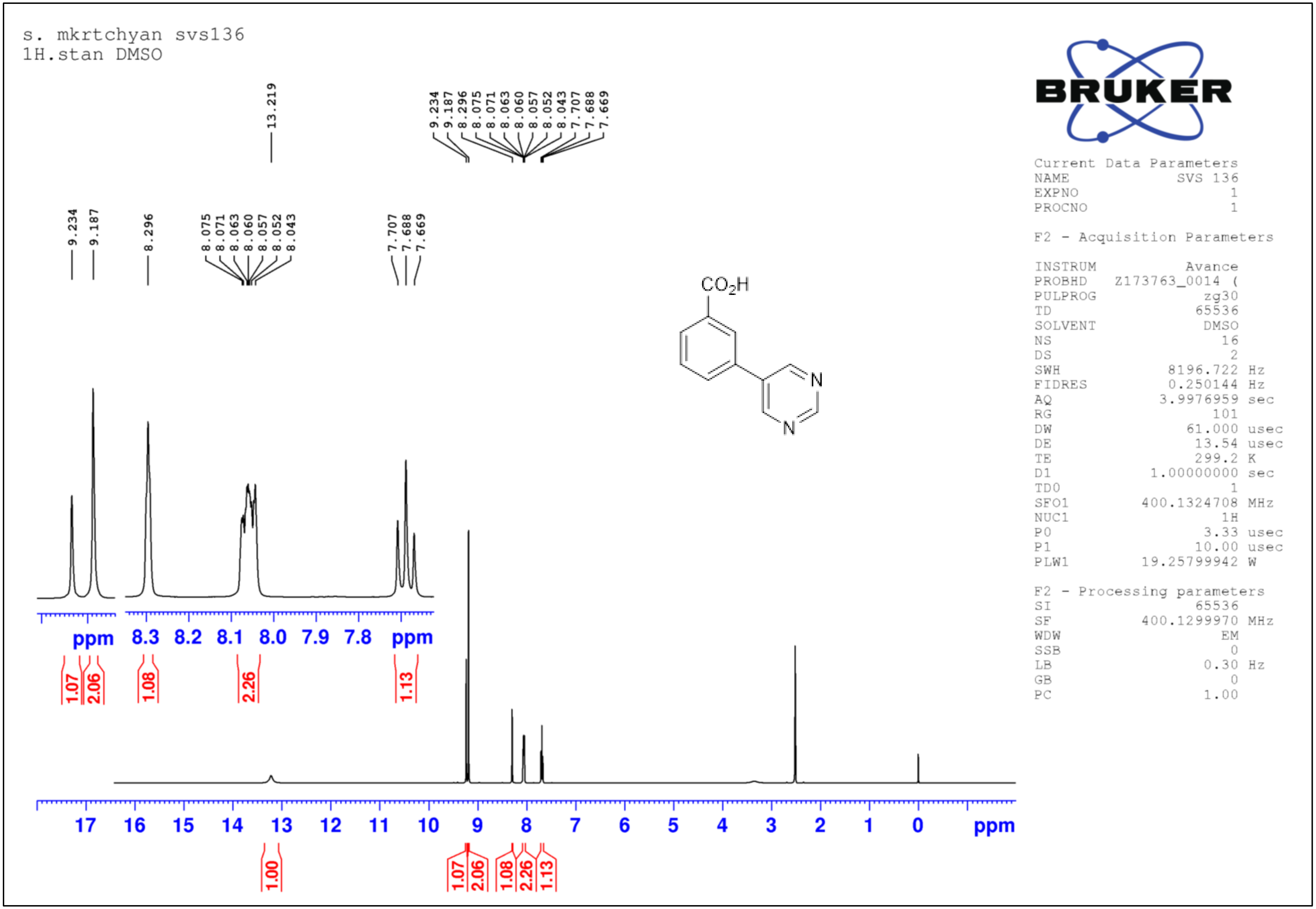

^{1}H NMR Spectrum of Compound 4k

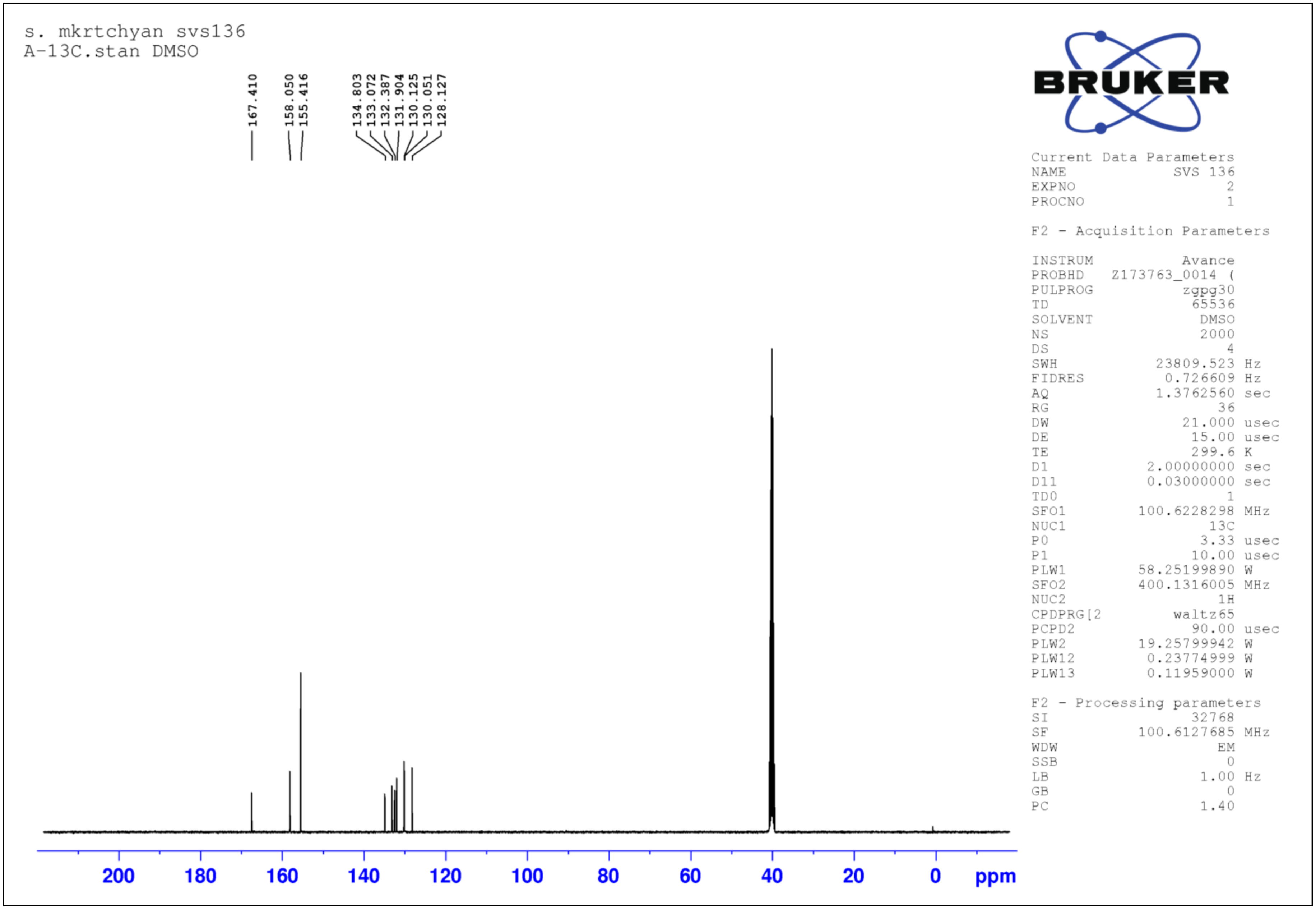

^{13}C NMR Spectrum of Compound 4k

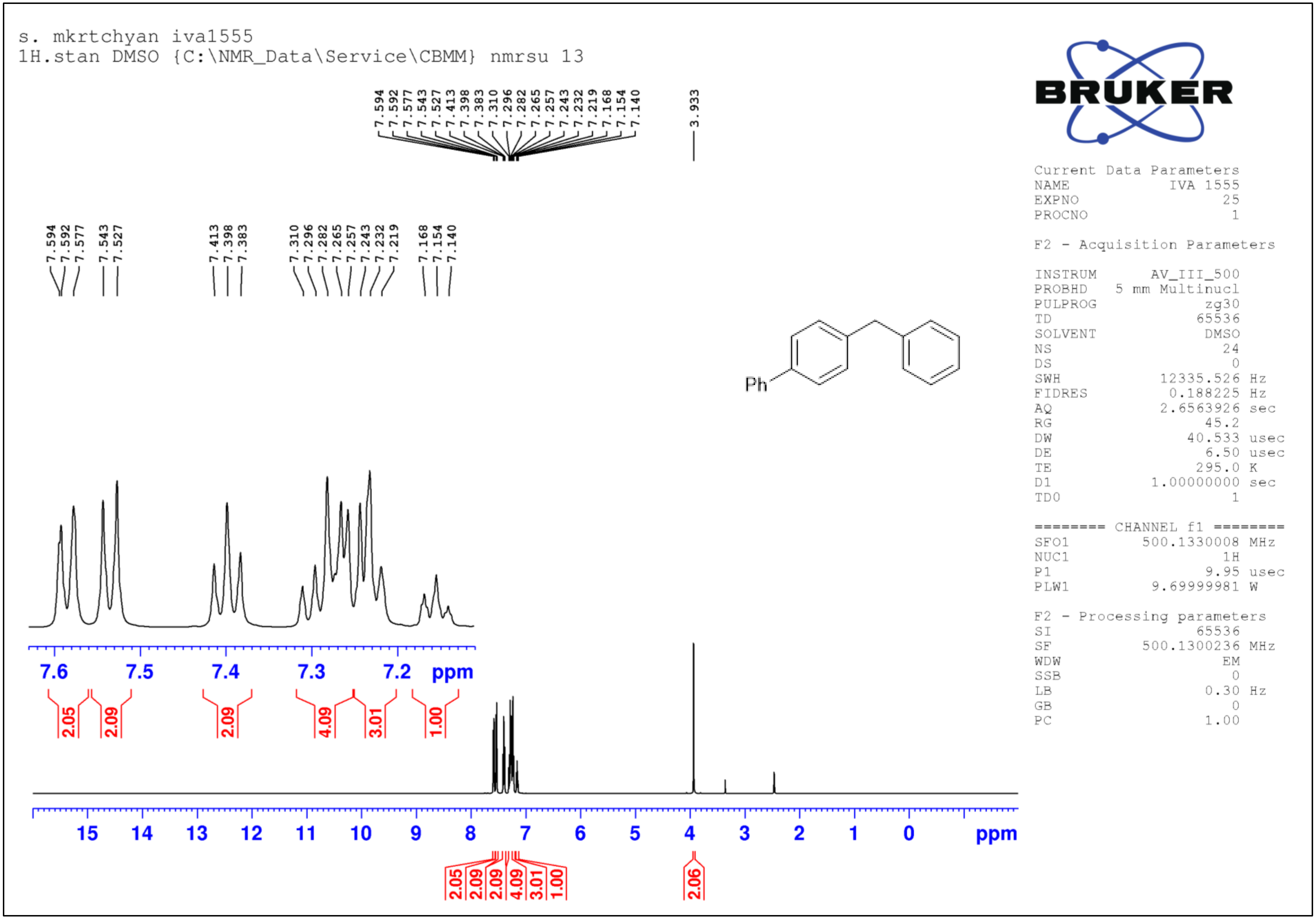

^{1}H NMR Spectrum of Compound 4l

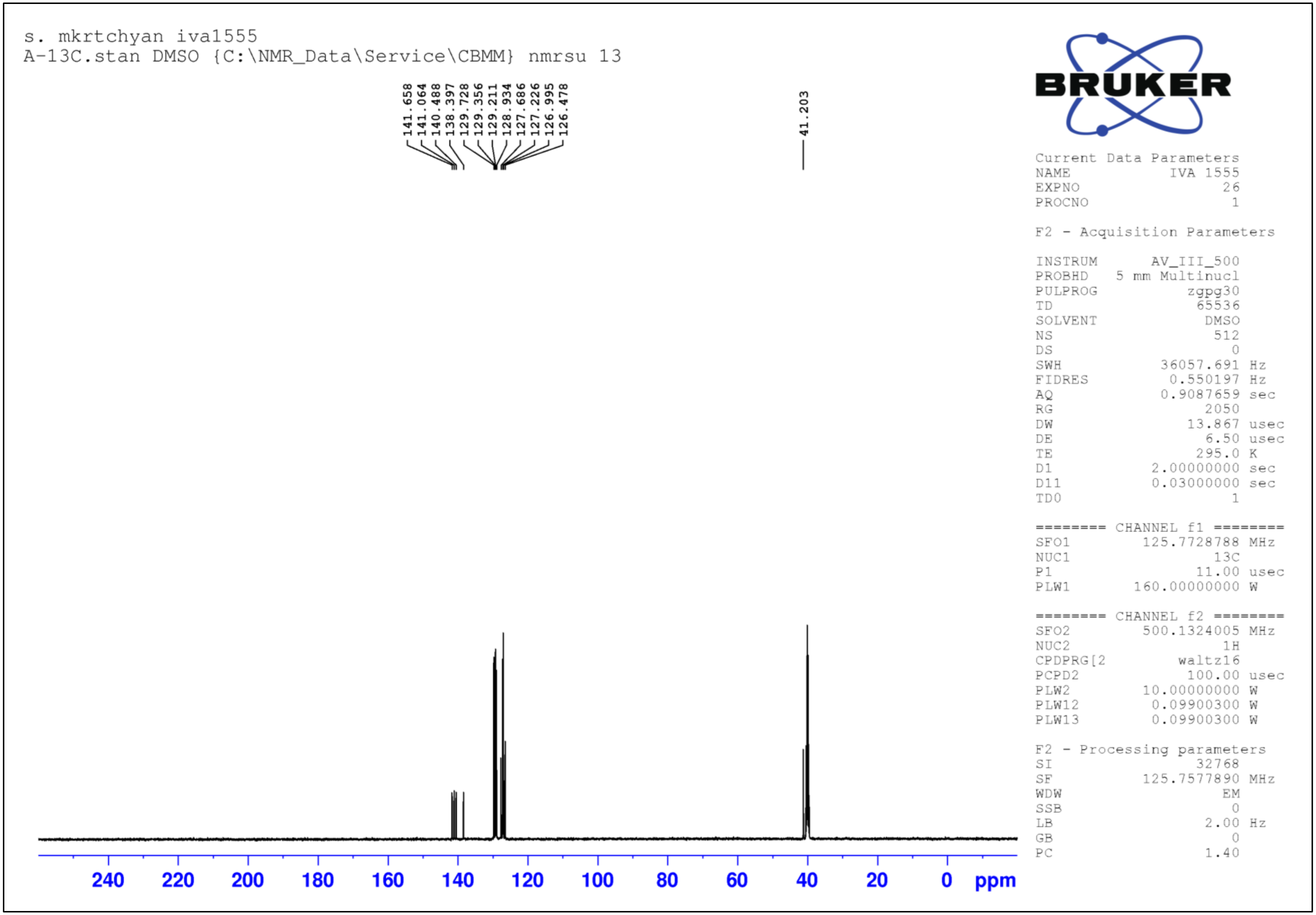

^{13}C NMR Spectrum of Compound 4l

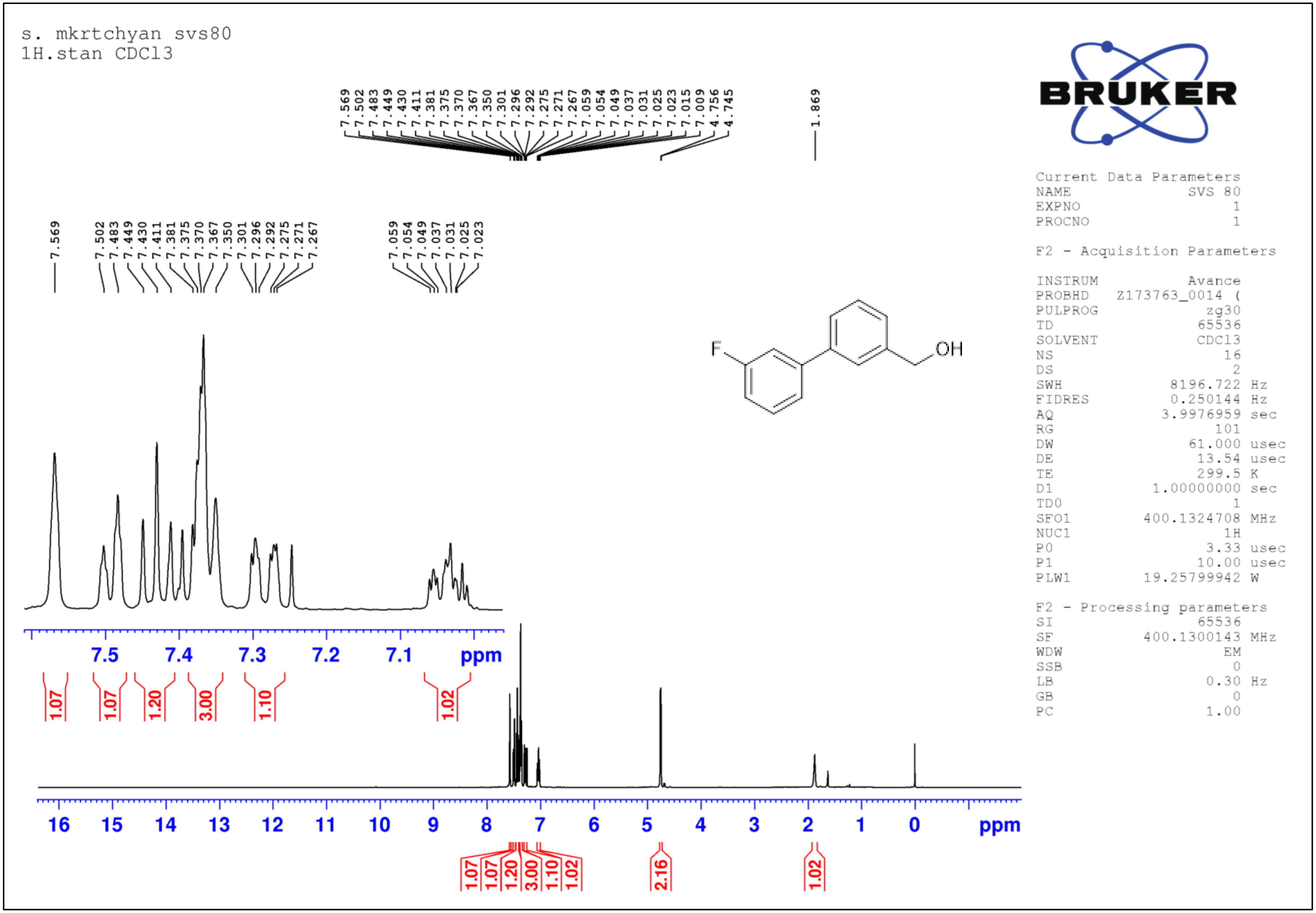

^{1}H NMR Spectrum of Compound 4m

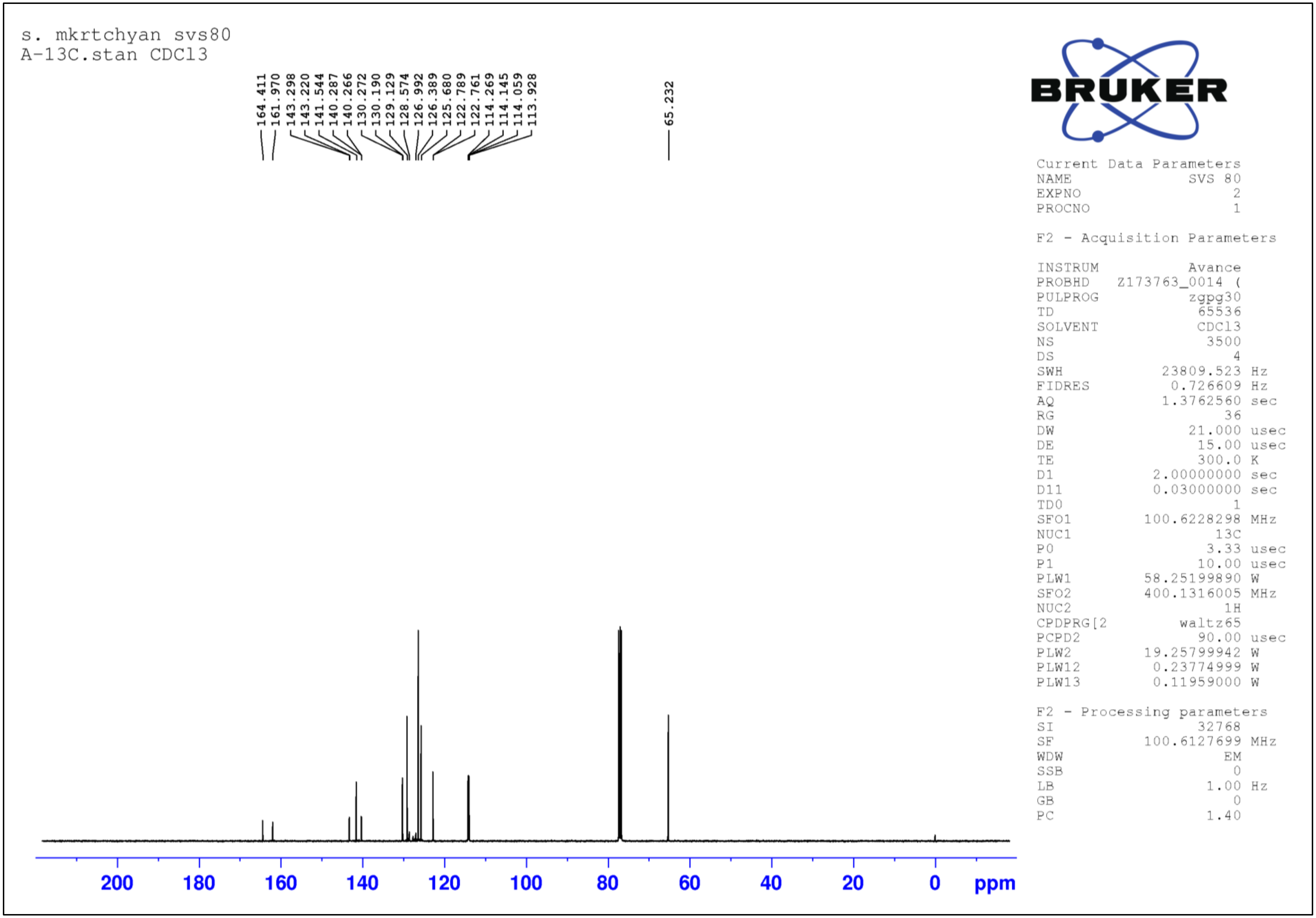

^{13}C NMR Spectrum of Compound 4m

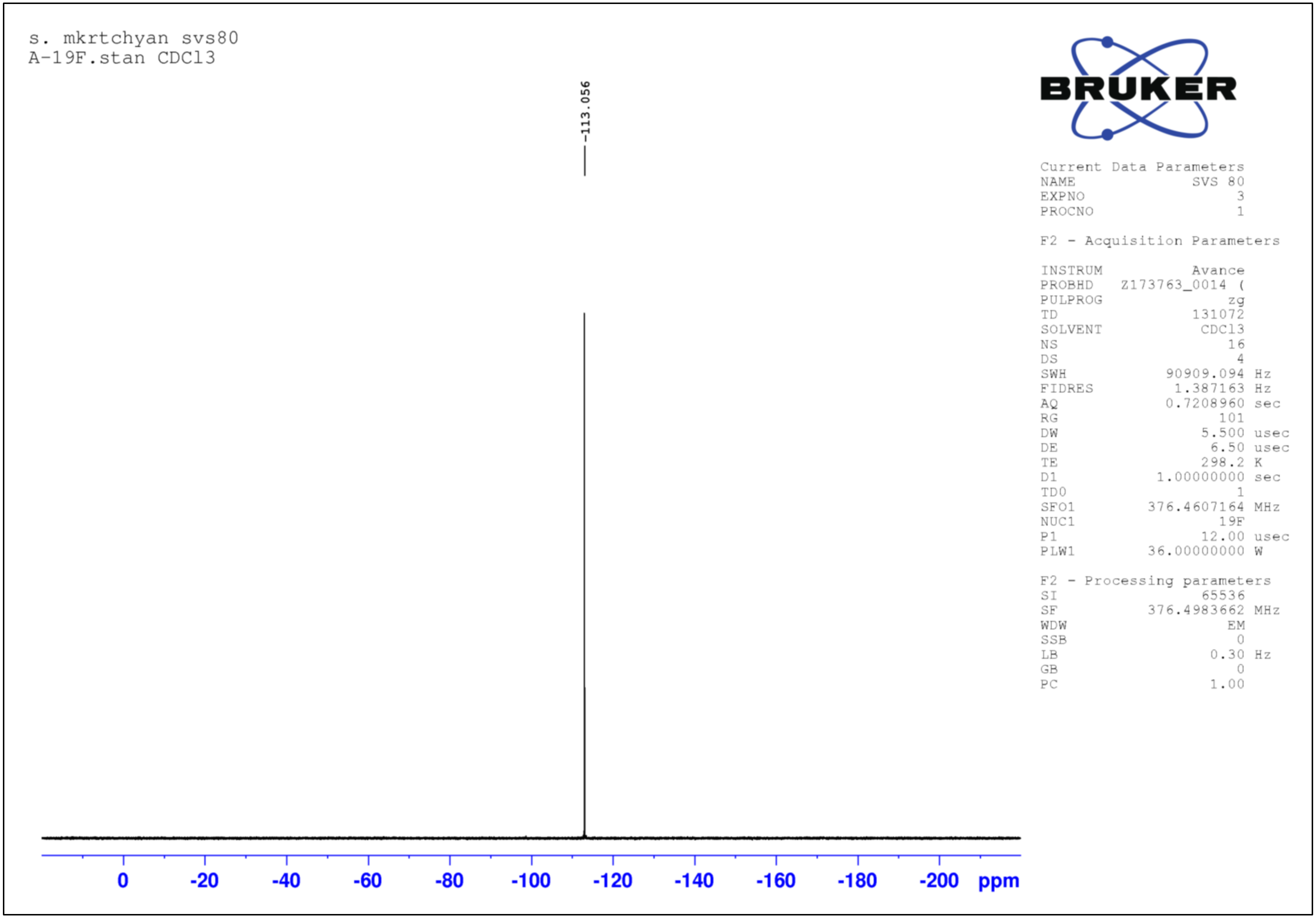

^{19}F NMR Spectrum of Compound 4m

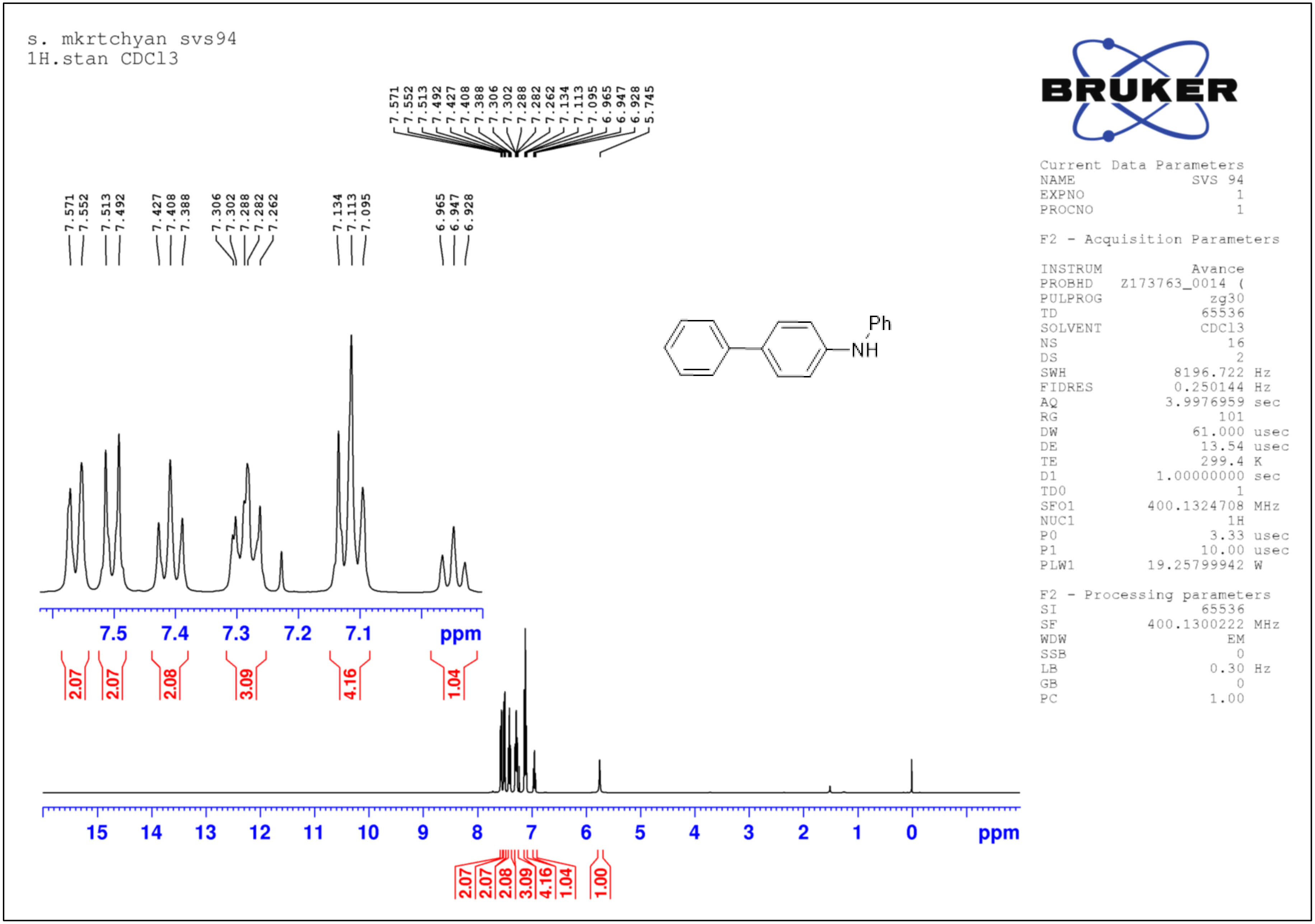

^{1}H NMR Spectrum of Compound 4n

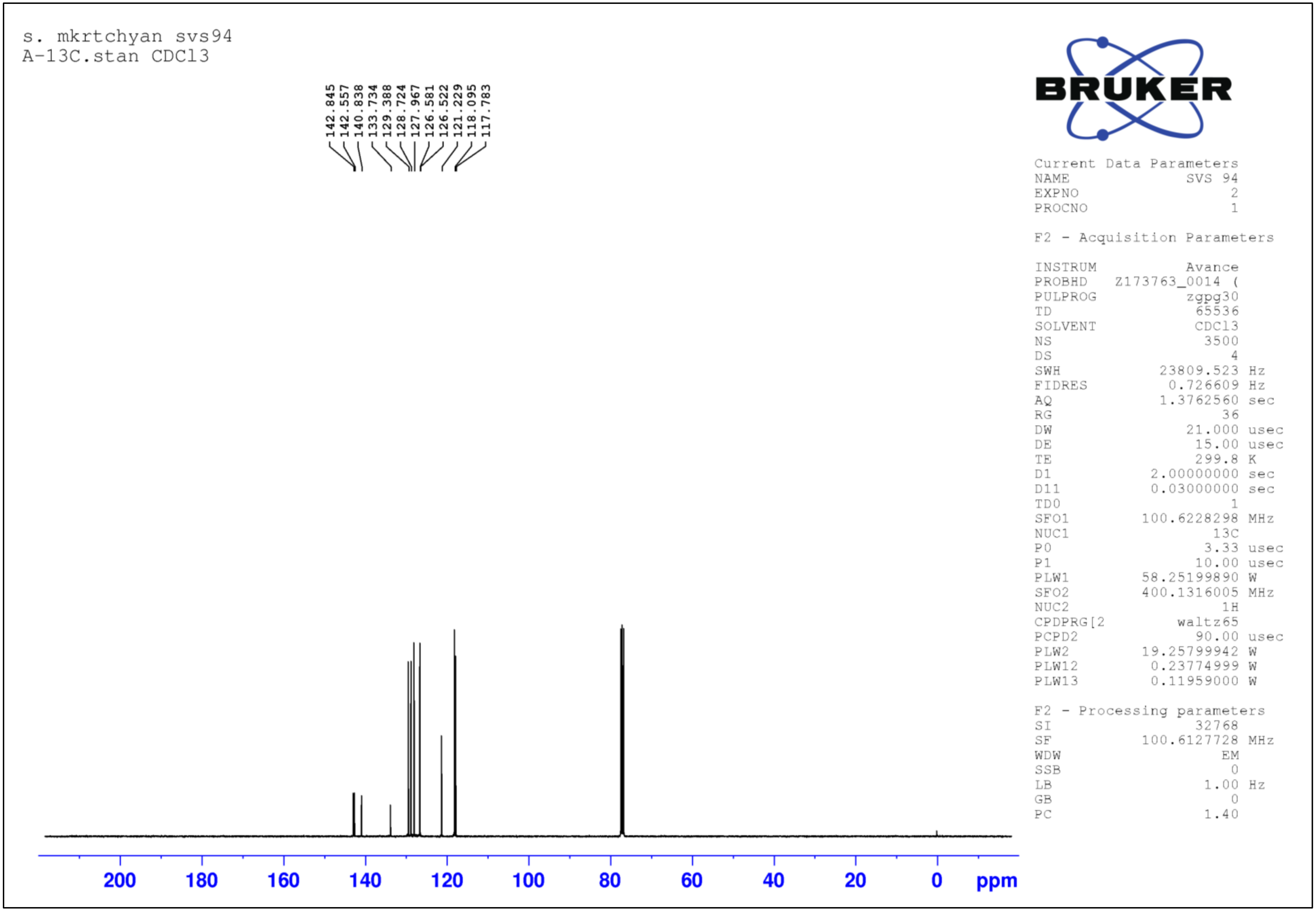

^{13}C NMR Spectrum of Compound 4n

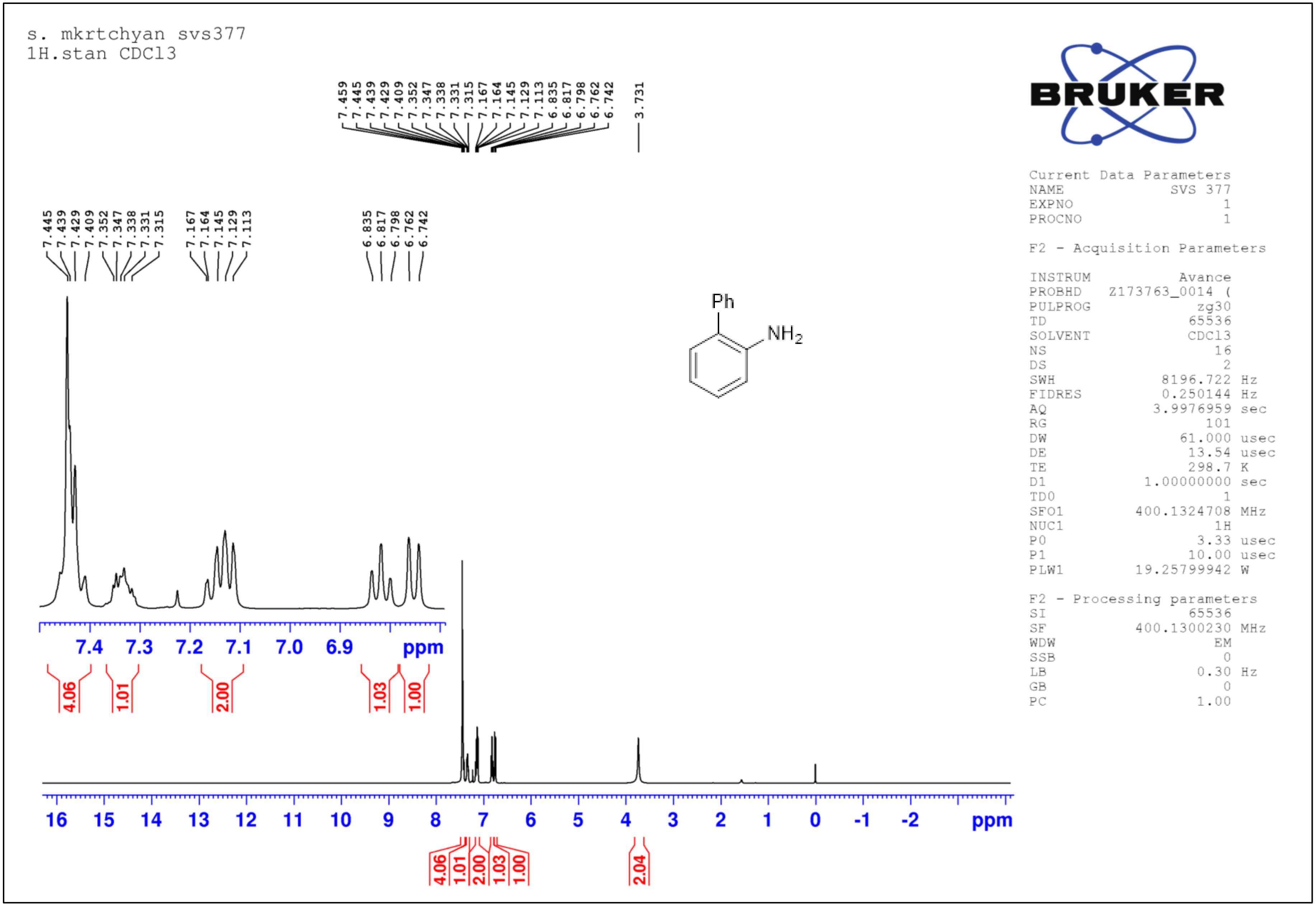

^{1}H NMR Spectrum of Compound 4o

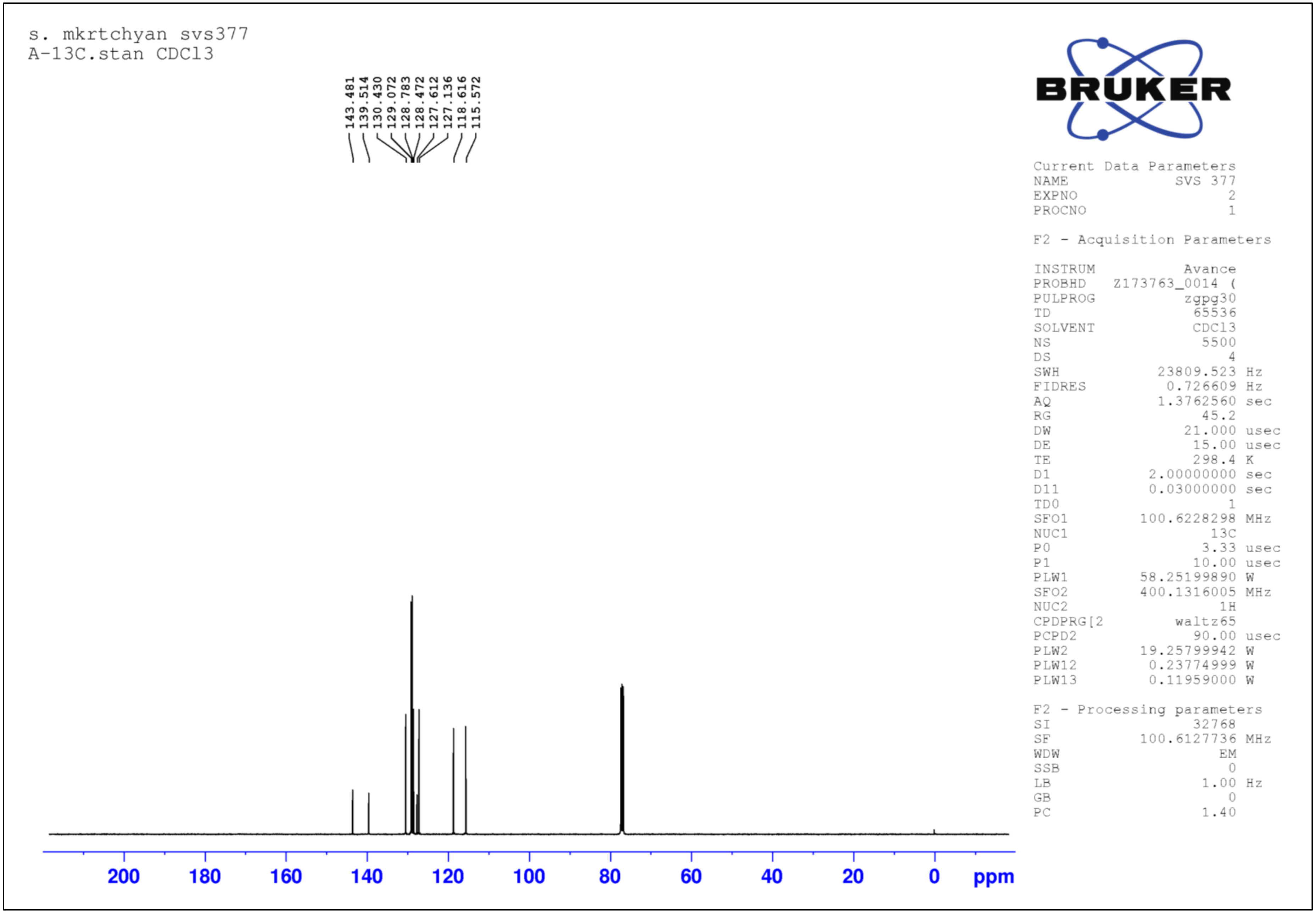

^{13}C NMR Spectrum of Compound 4o

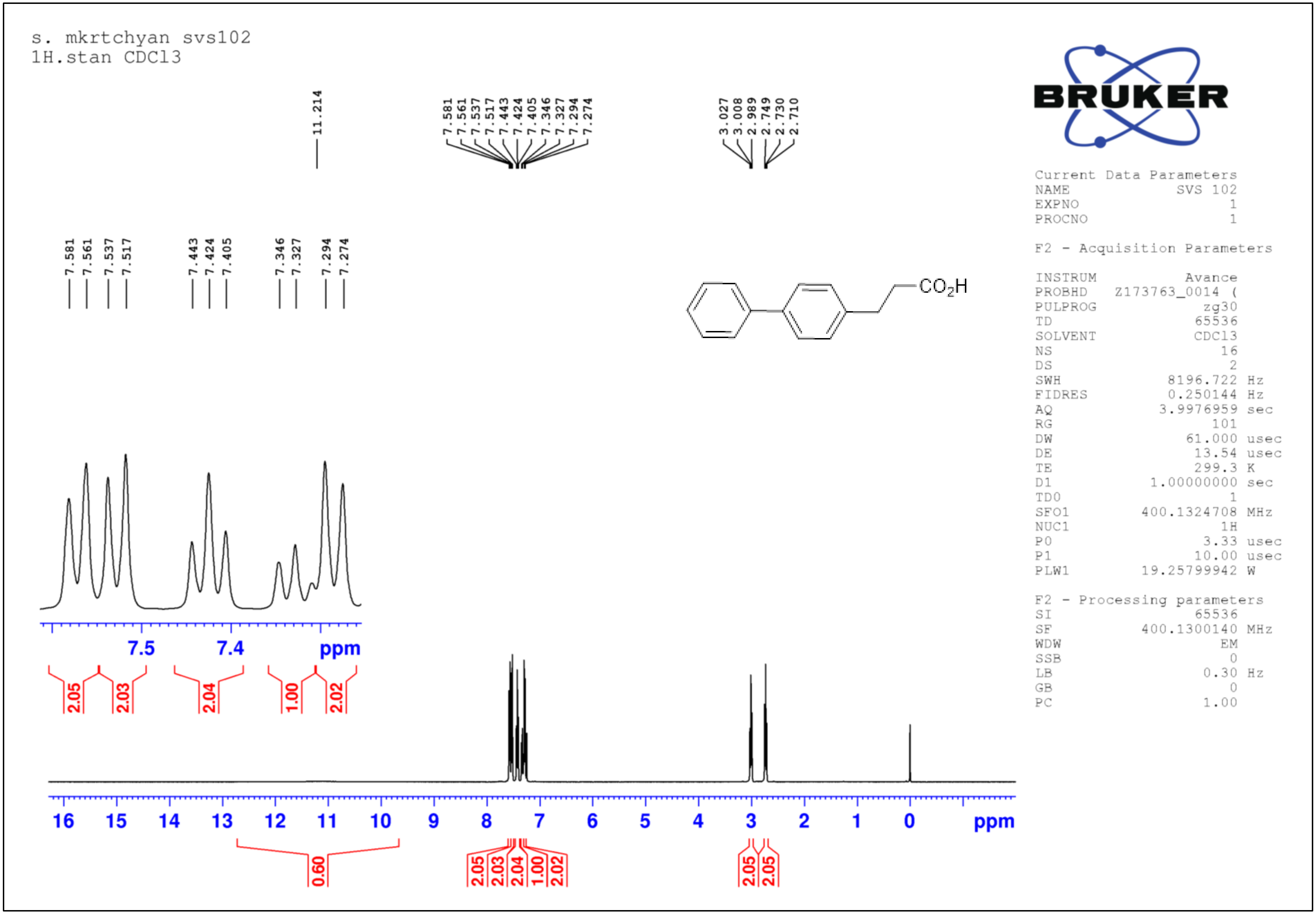

^{1}H NMR Spectrum of Compound 4p

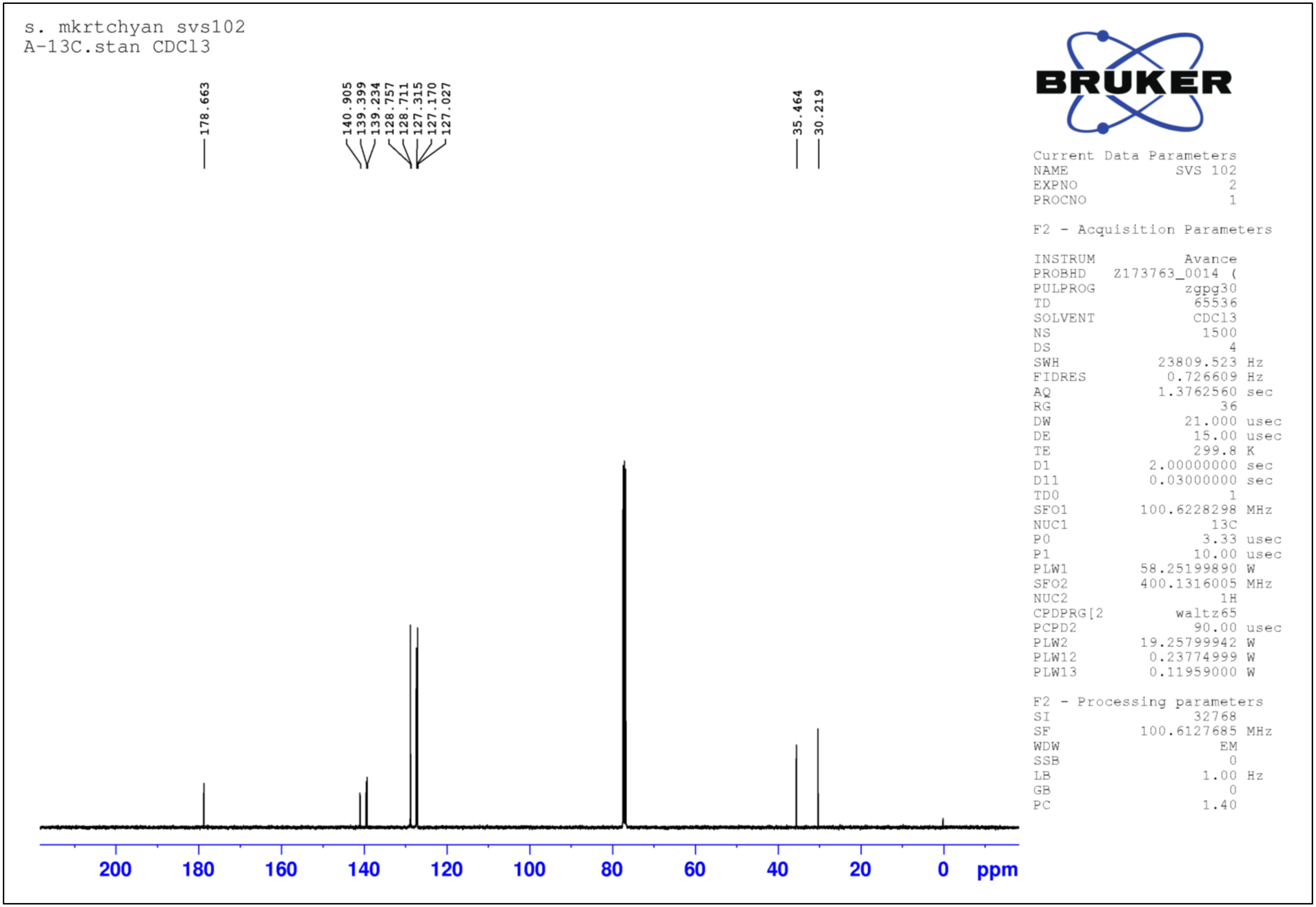

^{13}C NMR Spectrum of Compound 4p

SpinWorks 4: SVS 308 1H CDCl3

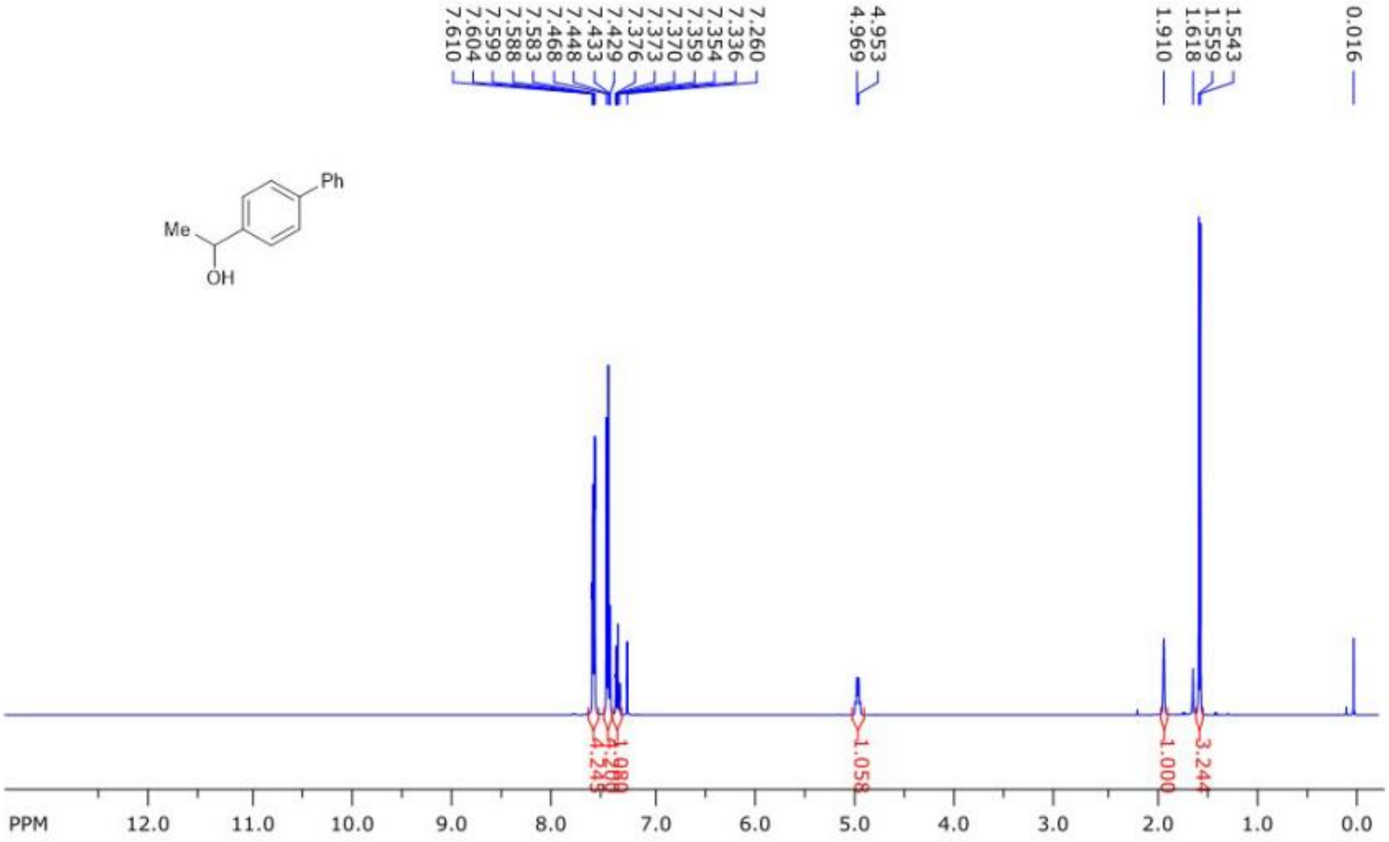

file: D:\NAPO\NMR\JELA\nmr\jn-308\1\fid expt: <zg30>
transmitter freq.: 400.132471 MHz
time domain size: 65536 points
width: 8196.72 Hz = 20.4850 ppm = 0.125072 Hz/pt
number of scans: 16

freq. of 0 ppm: 400.130009 MHz
processed size: 65536 complex points
LB: 0.300 GF: 0.0000
Hz/cm: 219.426 ppm/cm: 0.54838

^{1}H NMR Spectrum of Compound 4q

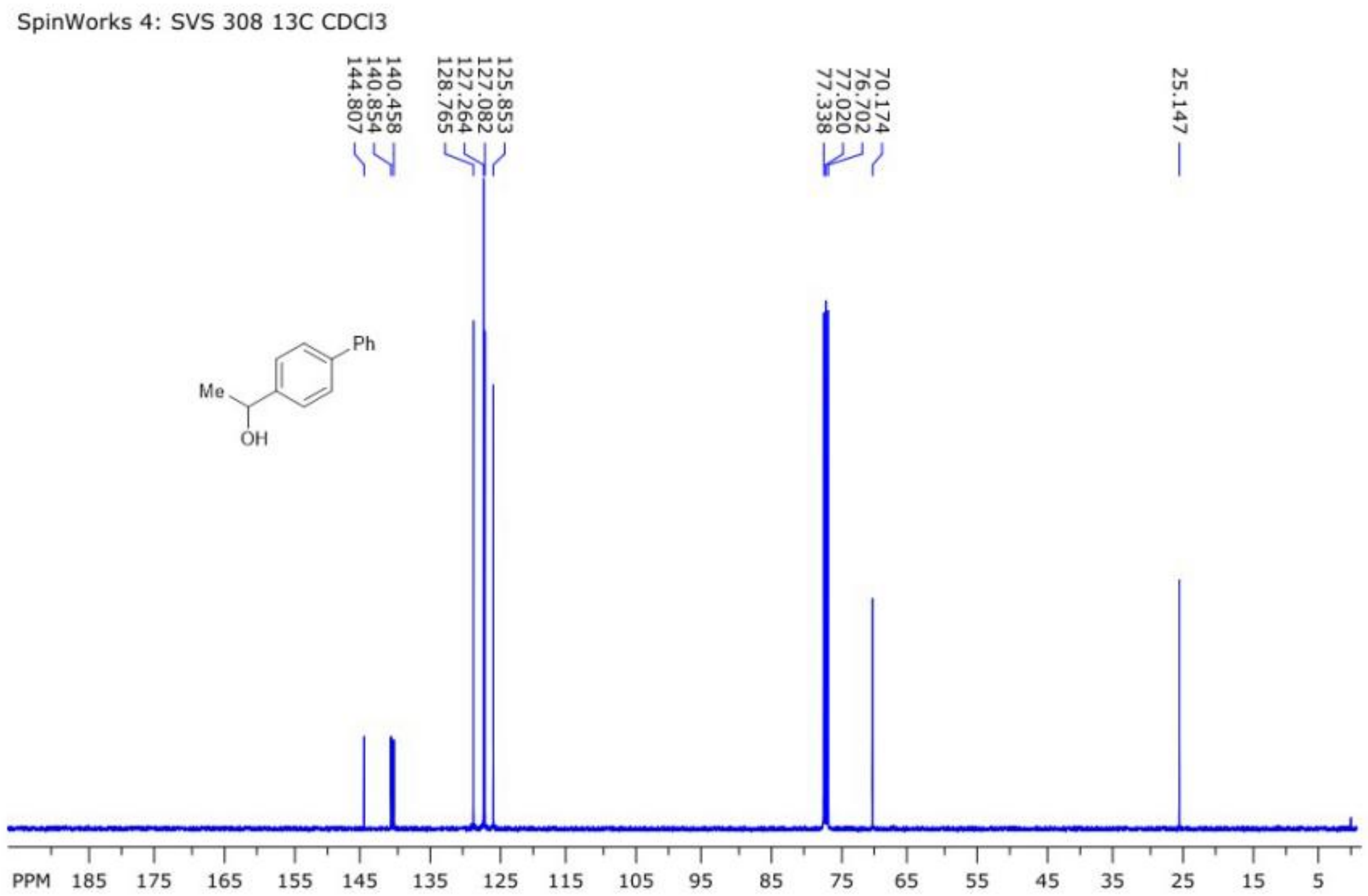

file: D:\NAPO\NMR\JELA\nmr\jn-308\2\fid expt: <zgpg30>
transmitter freq.: 100.622830 MHz
time domain size: 65536 points
width: 23809.52 Hz = 236.6215 ppm = 0.363305 Hz/pt
number of scans: 1500

freq. of 0 ppm: 100.612770 MHz
processed size: 32768 complex points
LB: 1.000 GF: 0.0000
Hz/cm: 796.460 ppm/cm: 7.91530

^{13}C NMR Spectrum of Compound 4q

SpinWorks 4: IVA 2073 1H DMSO

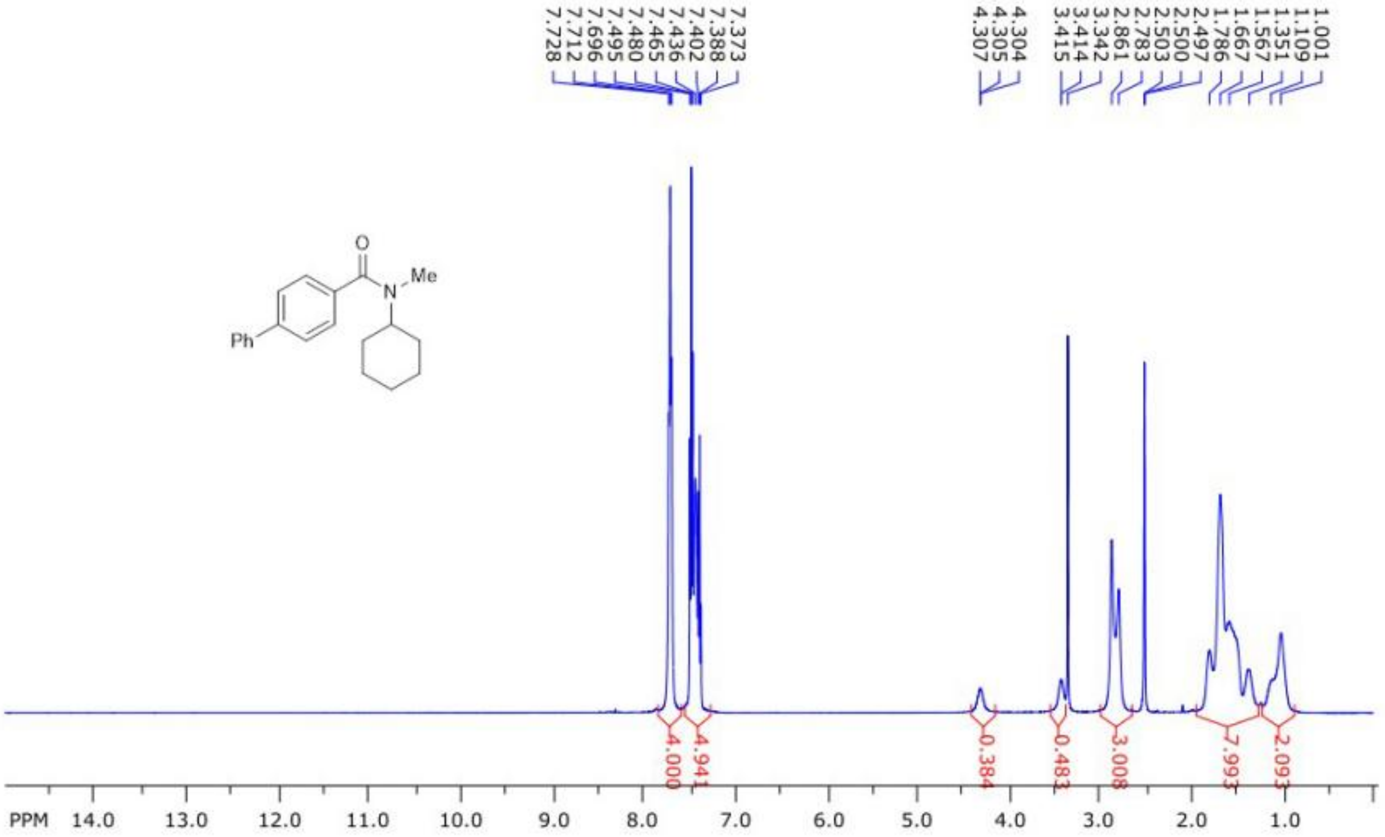

file: ...APO\NMR\500-2\mkr12607\29 2073\fid expt: <zg30>
transmitter freq.: 500.133001 MHz
time domain size: 65536 points
width: 12335.53 Hz = 24.6645 ppm = 0.188225 Hz/pt
number of scans: 24

freq. of 0 ppm: 500.130005 MHz
processed size: 65536 complex points
LB: 0.300 GF: 0.0000
Hz/cm: 300.747 ppm/cm: 0.60133

^{1}H NMR Spectrum of Compound 4r

SpinWorks 4: IVA 2073 13C DMSO

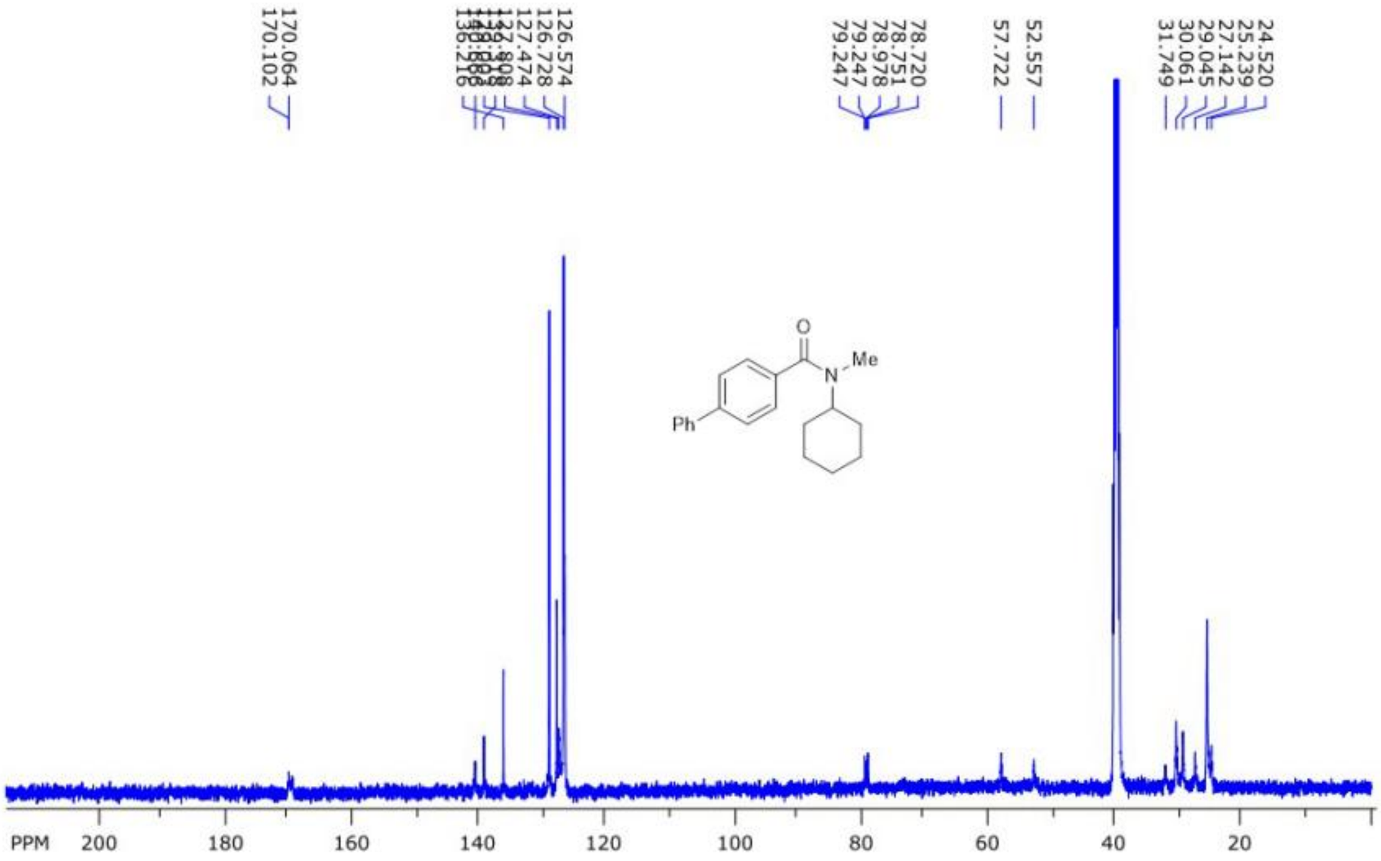

file: D:\NAPO\NMR\500-2\mkr12607\30\fid   expt: <zgpg30>
transmitter freq.: 125.772879 MHz
time domain size: 65536 points
width: 36057.69 Hz = 286.6889 ppm = 0.550197 Hz/pt
number of scans: 2048

freq. of 0 ppm: 125.757846 MHz
processed size: 32768 complex points
LB: 2.000   GF: 0.0000
Hz/cm: 1083.326   ppm/cm: 8.61335

^{13}C NMR Spectrum of Compound 4r

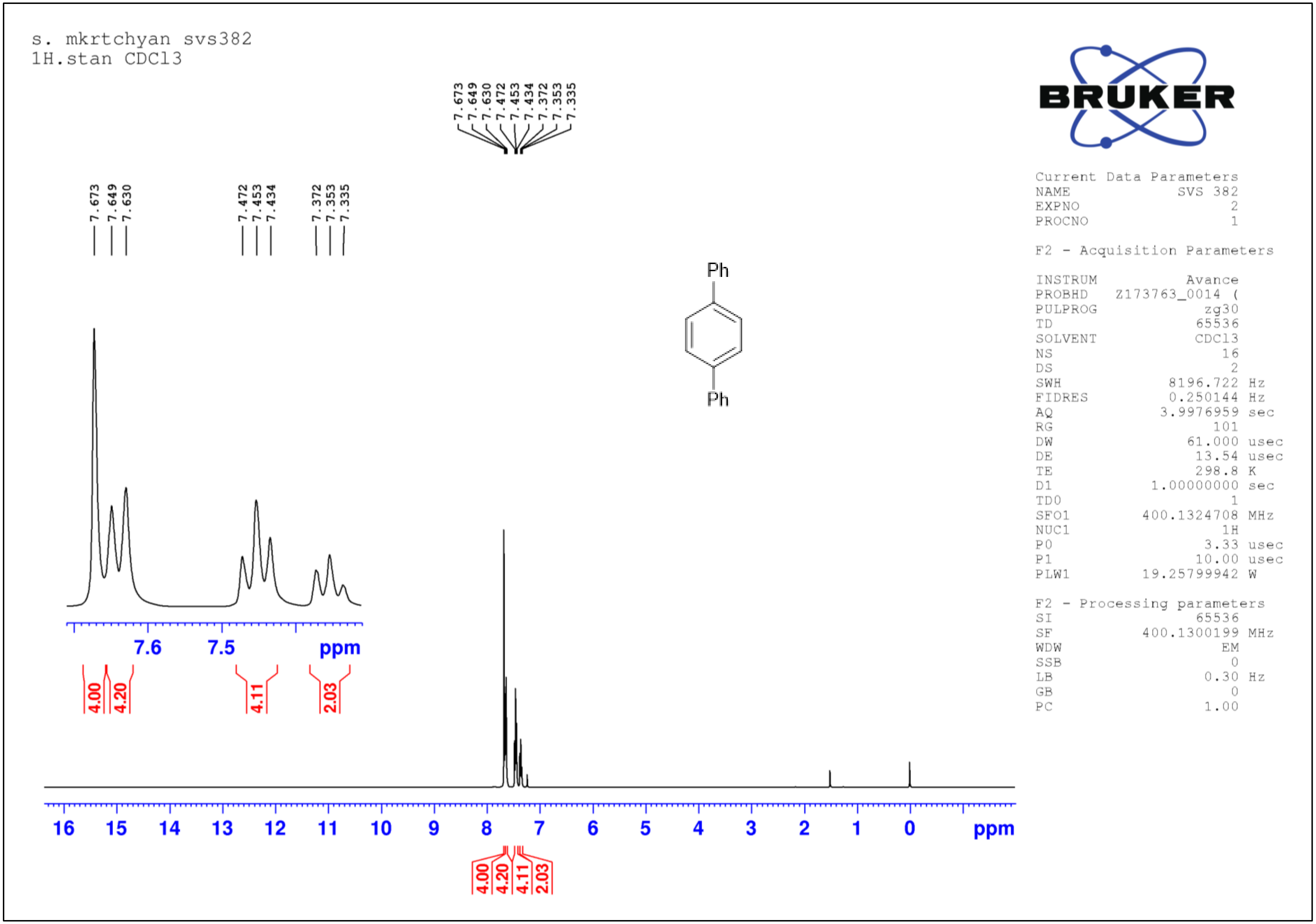

^{1}H NMR Spectrum of Compound 4s

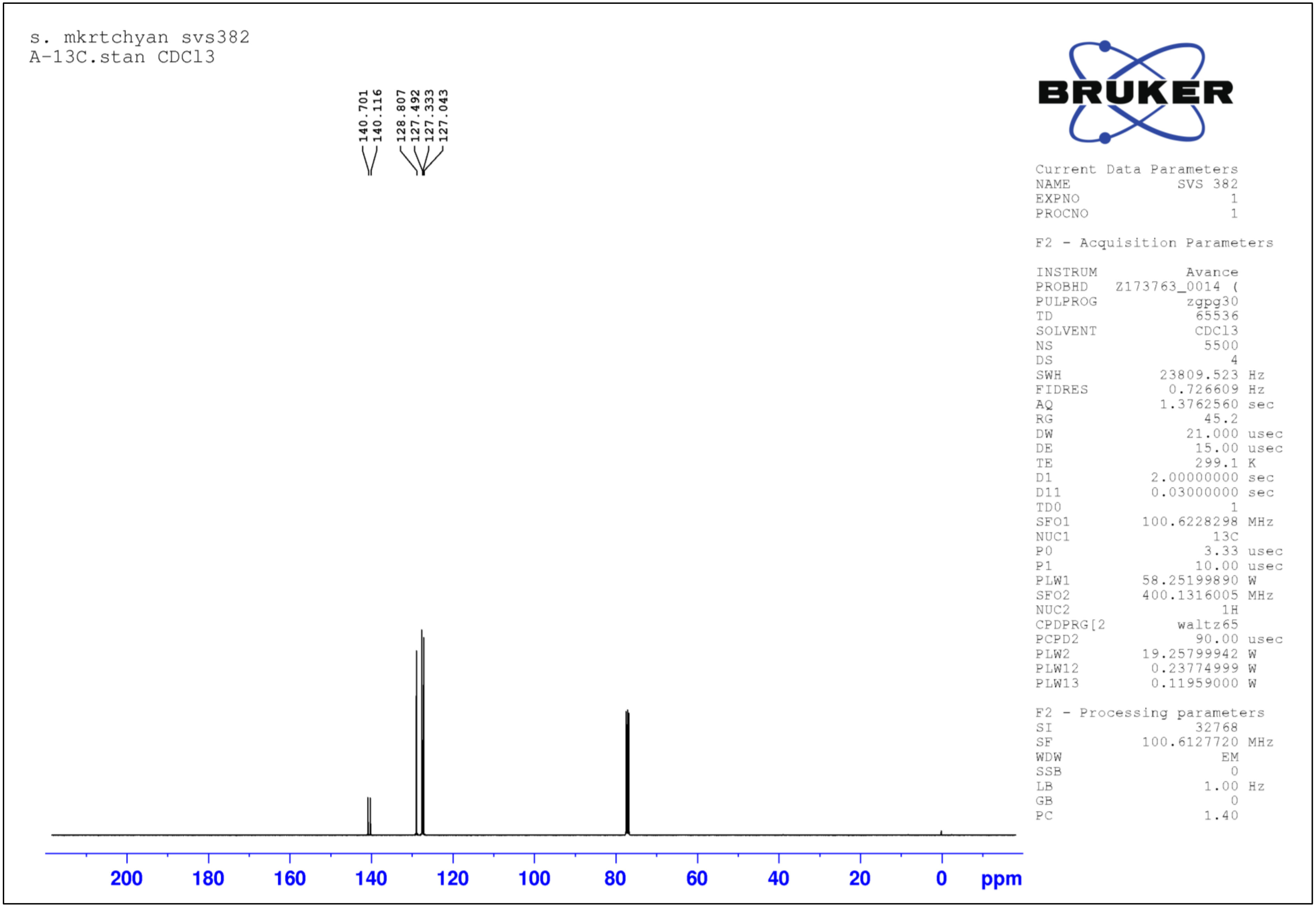

^{13}C NMR Spectrum of Compound 4s

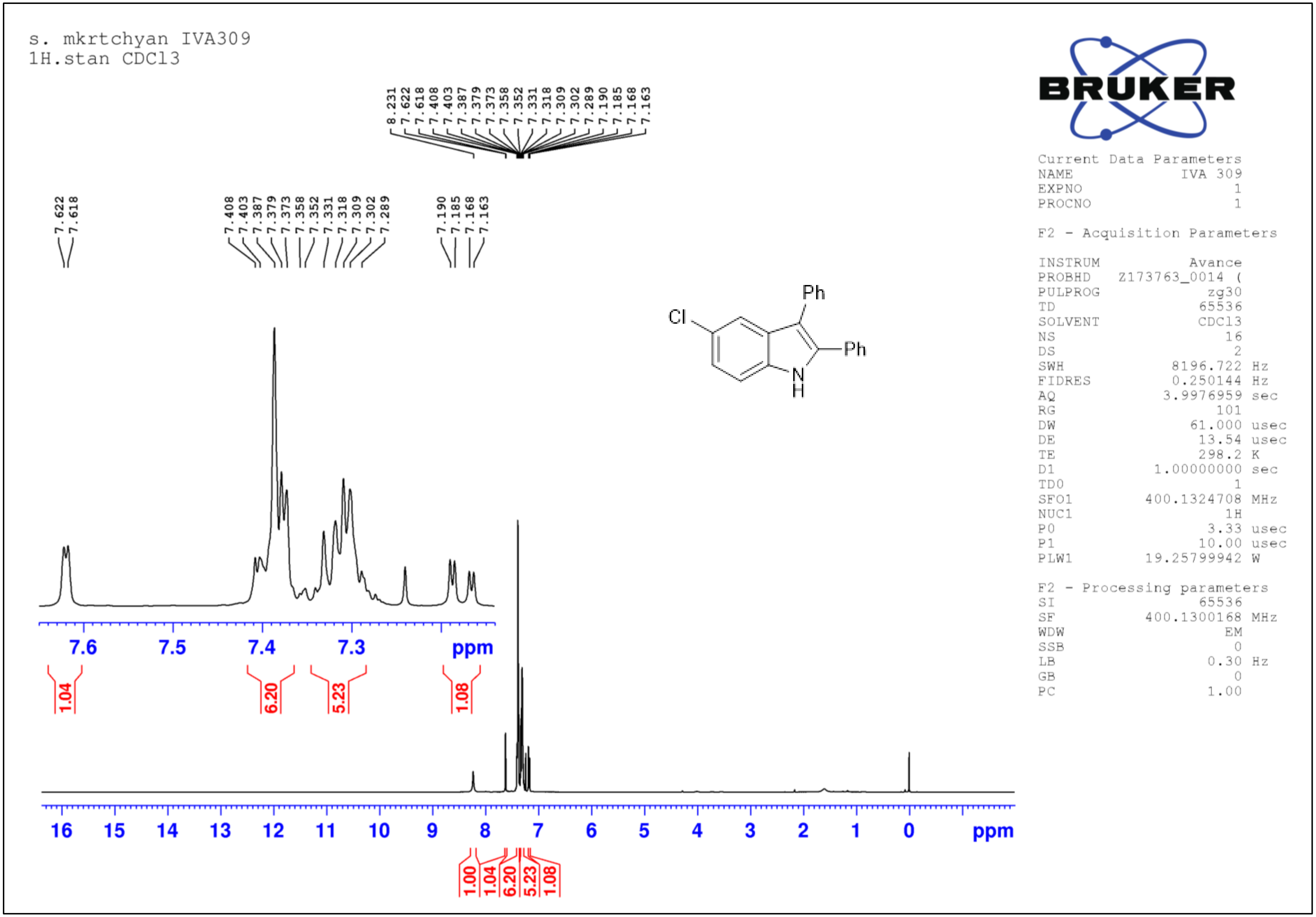

^{1}H NMR Spectrum of Compound 4t

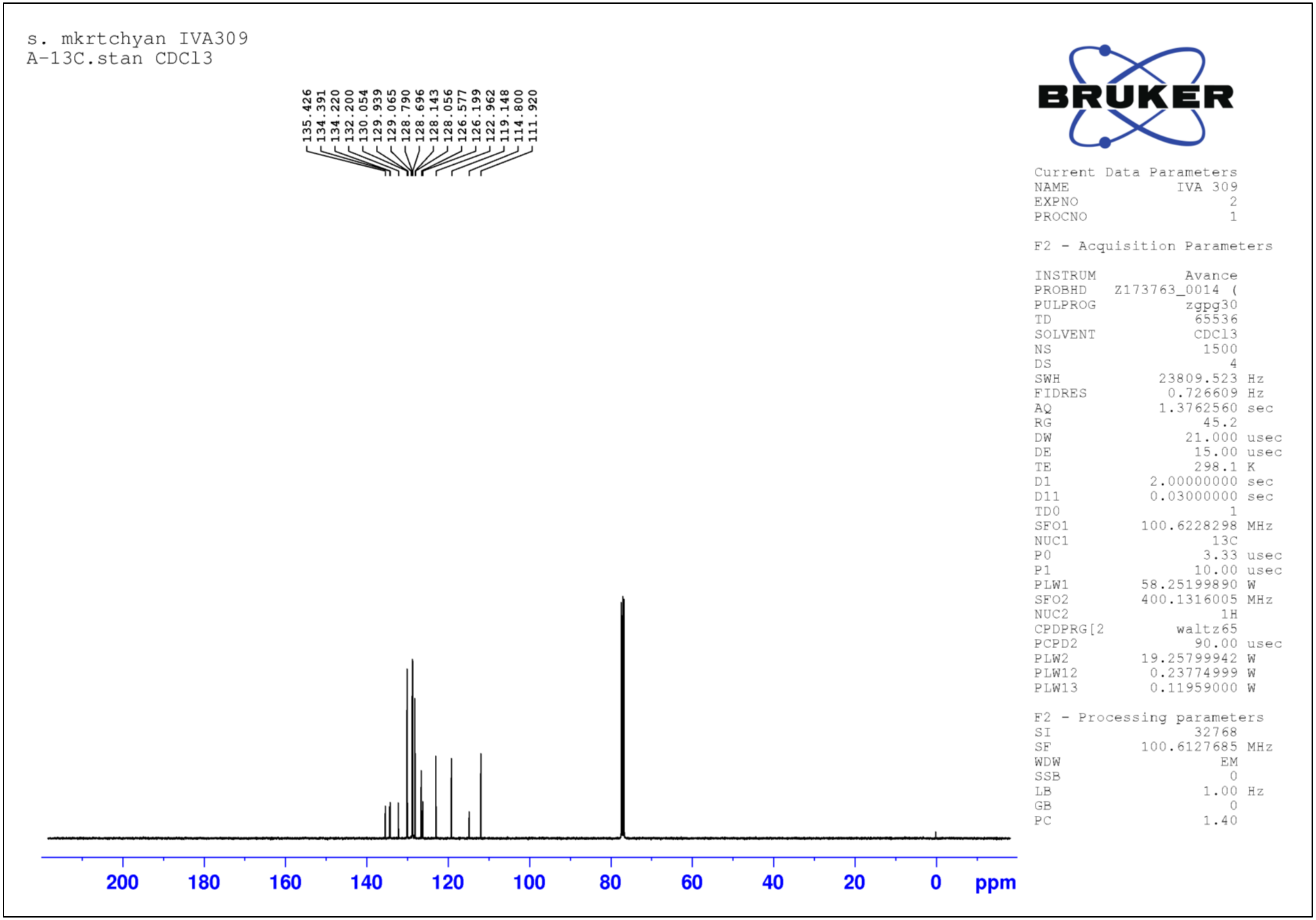

^{13}C NMR Spectrum of Compound 4t

(D) Computational DFT studies Data

Computational methodology

A DFT study is performed to confirm the proposed mechanism Ru-catalyzed deoxygenative borylation of unprotected phenols via C-O bond cleavage under mechanochemical conditions. All the density functional theory (DFT) simulation are carried out using package Gaussian09 [1]. The geometry optimizations are simulated at B3LYP density functional without any symmetry constraints. B3LYP is a low cost functional, which is frequently implemented for the DFT calculations of mechanistic studies, electronic parameters, and thermodynamic stabilities [2-4]. All the complexes involved are optimized by using GenECP methods, i.e., for heavy atoms (Ru & Zr) LanL2DZ basis set is assigned and 6-31G(d,p) pople style basis set is assigned for lighter atoms (C, O, N, H & B) [5]. Moreover, frequency analysis is performed to confirm transition state and intermediate structures over potential energy surface i.e., the presence of one negative frequency in the Hessian matrix corroborates saddle point (transition state), whereas absence of negative frequency validates the minima nature of intermediate species. Furthermore, transition state is also identified through negative frequency animation i.e., the motion of eigen vector corresponds to reaction axis [6]. All the free energies are presented in kcal/mol, while bond distances are expressed in angstrom (Å).

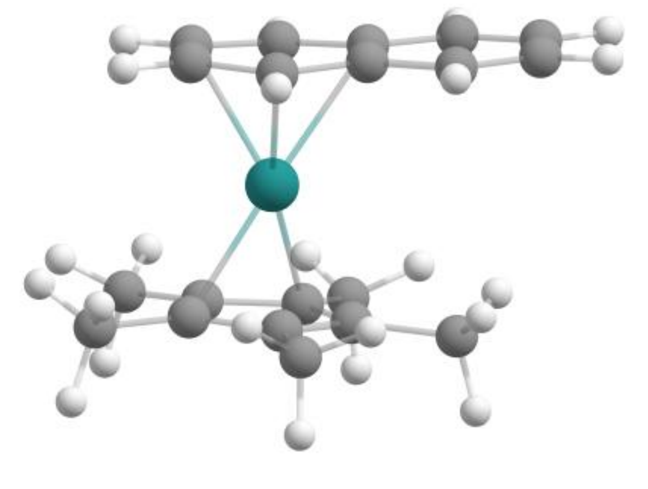	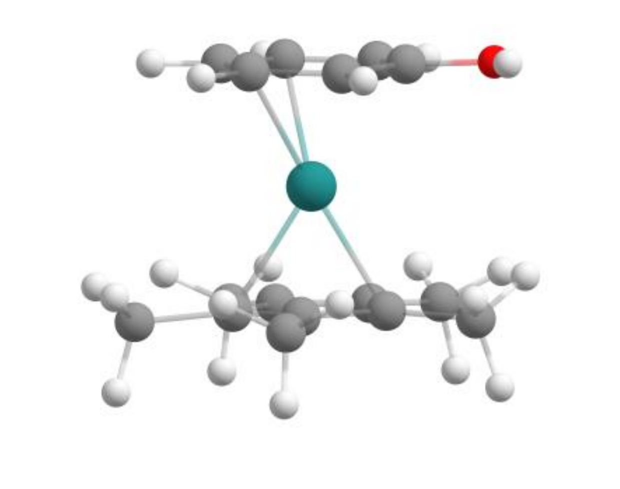
**C-0 (R)**	**C-I (Int 1)**

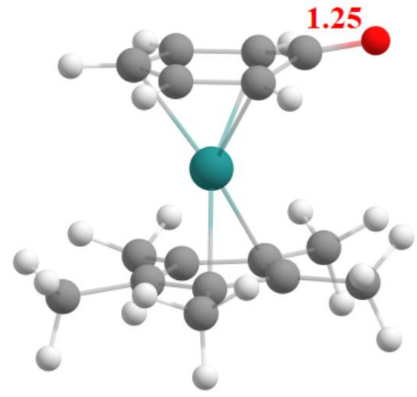

C-II (Int 2)

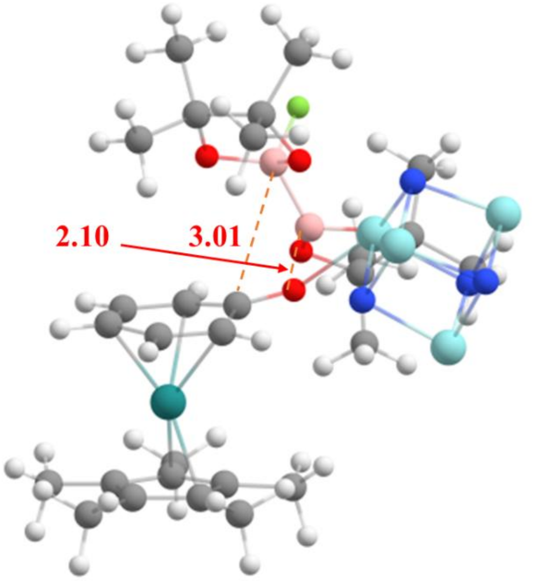

Int 3

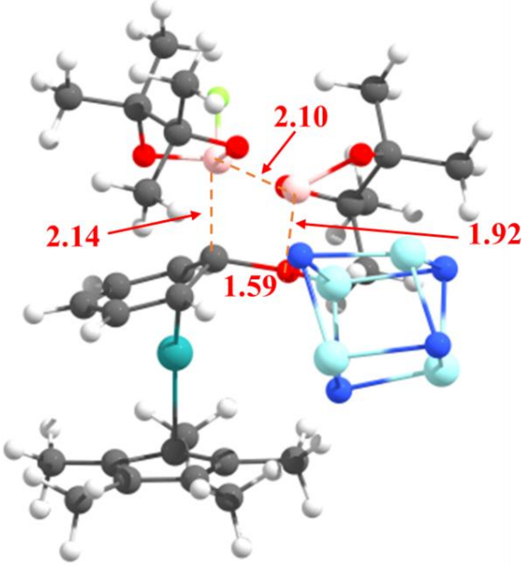

TS

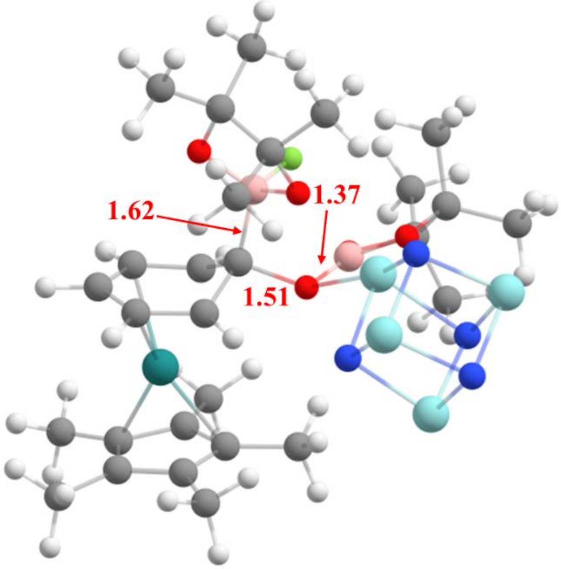

Int 4

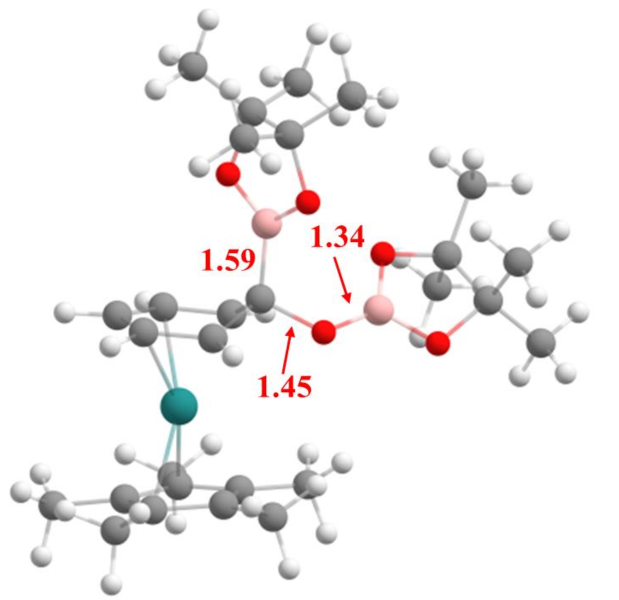

C-III (Int 5)

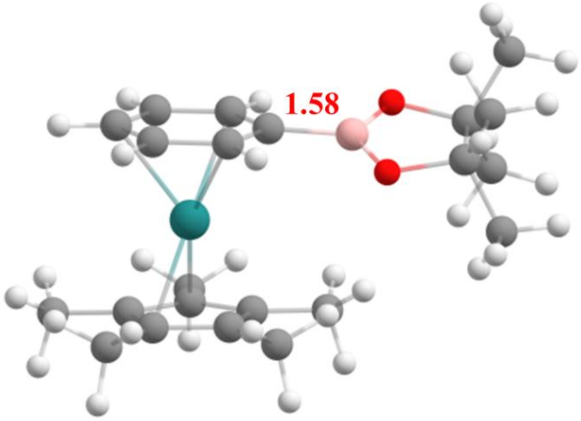

C-IV (Int 6)

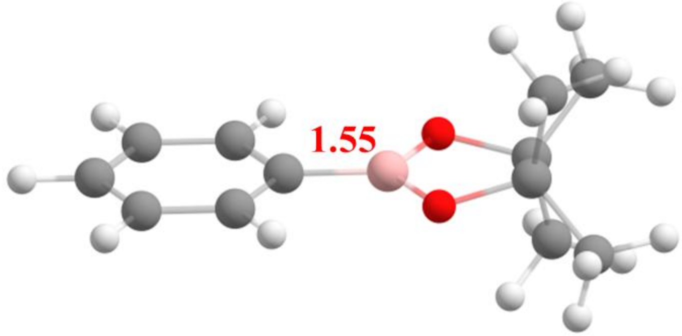

Final product

Figure S4. Optimized geometries of reactants, intermediates, transitions state and final product along with important bond lengths.

References

1. Frisch, M., et al., *01; Gaussian, Inc.* Wallingford, CT, 2009.
2. Bibi, S., et al., *Structure and electronic characterization of pristine and functionalized single wall carbon nanotube interacting with sulfide ion: A density functional theory approach.* Journal of Molecular Liquids, 2022. **366**: p. 120144.
3. Mkrtchyan, S., et al., *Mechanochemical arylative detrifluoromethylation of trifluoromethylarenes.* Organic & Biomolecular Chemistry, 2023. **21**(32): p. 6549-6555.
4. Mkrtchyan, S., et al., *Metal-Free Supramolecular Reduction of Nitro Compounds into the Cucurbit[7]uril Cavity: Testing the Enabling Technique in Aqueous Media.* ACS Sustainable Chemistry & Engineering, 2023. **11**(23): p. 8406-8412.
5. Danish, M., et al., *Synthesis, single-crystal X-ray diffraction, and in vitro biological evaluation of sodium, cobalt, and tin complexes of o-nitro-/o-methoxyphenylacetic acid: experimental and theoretical investigation.* Monatshefte für Chemie-Chemical Monthly, 2020. **151**(11): p. 1727-1736.
6. Mukhtar, A., S. Sarfaraz, and K. Ayub, *Organic transformations in the confined space of porous organic cage CC2; catalysis or inhibition.* RSC advances, 2022. **12**(37): p. 24397-24411.

Cartesian coordinates

C-0

1 1

C	-0.80829400	-1.78851500	0.12904200
C	-1.46784400	-1.07619900	1.19134000
C	-2.33343900	-0.08995000	0.59212500
C	-1.26486100	-1.25066200	-1.12941300
C	-2.21225300	-0.20272400	-0.84438300
Ru	-0.25578200	0.37258600	0.00441800
C	-1.36159700	-1.38000800	2.65639100
H	-1.53935700	-0.49312100	3.26869800
H	-2.11017500	-2.13016000	2.93968800

H	-0.38033700	-1.78214400	2.91815600
C	0.13544200	-2.94095800	0.29504500
H	0.87150200	-2.98058200	-0.51103800
H	0.67466500	-2.88987200	1.24316400
H	-0.42161500	-3.88597800	0.28097900
C	-0.90512800	-1.77385600	-2.48826100
H	-1.56654200	-2.60561000	-2.76021900
H	-1.00980700	-1.00652300	-3.25846900
H	0.12106000	-2.14748000	-2.51904600
C	-3.02222000	0.55216300	-1.85581100
H	-3.30142600	1.54446500	-1.49457400
H	-2.48479800	0.67264200	-2.79908500
H	-3.95020200	0.00892500	-2.07304400
C	-3.29128500	0.80101200	1.32527800
H	-3.48546600	1.72617200	0.77800900
H	-4.25277400	0.28958800	1.45748800
H	-2.92131600	1.06485300	2.31856700
C	3.79197500	-0.88526000	0.71093100
C	2.91875400	-0.10187800	1.41827500
C	2.00531800	0.76150700	0.73517200

C	2.00677300	0.77494700	-0.71249700
C	2.92096400	-0.07641300	-1.40994800
C	3.79311100	-0.87242000	-0.71539700
H	1.03643000	1.55744600	2.51676800
H	4.49924500	-1.51894800	1.23646200
H	2.92481900	-0.10061400	2.50410000
C	1.06526800	1.59289300	1.43304500
C	1.06791200	1.61888200	-1.39603600
H	2.92894400	-0.05515400	-2.49552400
H	4.50133300	-1.49643400	-1.25117000
C	0.22927700	2.50327300	-0.68520500
C	0.22833400	2.49042200	0.73750600
H	1.04055700	1.60373500	-2.48027700
H	-0.44765000	3.15820400	-1.22151600
H	-0.44990100	3.13527300	1.28432300

C-I

1 1

C	1.43891600	0.55538300	1.22732500
C	1.93673500	-0.56097200	0.46372900
C	1.79772100	-0.24147400	-0.93574600

C	0.99144200	1.56677800	0.29807400
C	1.21887600	1.07462600	-1.03673800
Ru	-0.22456500	-0.26275700	0.00545700
C	2.59100900	-1.78897400	1.02333600
H	2.45970600	-2.65336600	0.36866200
H	3.66947600	-1.62129800	1.13370200
H	2.19736500	-2.04663100	2.00903900
C	1.49269600	0.69070900	2.72020800
H	0.70095600	1.34066100	3.09948100
H	1.40507900	-0.27703000	3.21908700
H	2.45091800	1.13074100	3.02242500
C	0.47753100	2.93154600	0.64942800
H	1.29175300	3.66517700	0.61001200
H	-0.29781100	3.26289100	-0.04611400
H	0.06302100	2.95903400	1.65973700
C	0.98854800	1.84170900	-2.30471000
H	0.77642000	1.18070500	-3.14787800
H	0.16023900	2.54693100	-2.20754000
H	1.88563500	2.41993300	-2.55824600
C	2.28345400	-1.07895700	-2.08125400

H	1.70323600	-0.90138500	-2.98941300
H	3.32884700	-0.83452400	-2.30655900
H	2.23918700	-2.14611200	-1.85262600
C	-1.64334500	-1.59761900	1.21466500
C	-2.19117900	-0.28697900	1.21423200
H	-0.93083600	-3.24821500	-0.00735200
C	-1.36922200	-2.25737700	-0.00905100
C	-2.51313800	0.35349500	-0.01416200
C	-2.16743500	-0.28045900	-1.23868600
C	-1.62270200	-1.58967200	-1.23453300
H	-2.35490300	0.24564800	-2.16732700
H	-1.37824900	-2.06882600	-2.17532100
H	-1.41805800	-2.08099000	2.15808100
H	-2.38723800	0.21636000	2.15592300
O	-3.08143400	1.57064700	-0.09264000
H	-3.29186300	1.91584400	0.78654800

C-II

0 1

C	-1.49409900	0.34368600	-1.24891200
C	-1.99834900	-0.56846600	-0.25410400

C	-1.75888200	0.01174300	1.04230700
C	-0.95805400	1.50123500	-0.56515000
C	-1.12162600	1.29114300	0.84530100
Ru	0.20430800	-0.29665300	0.01519800
C	-2.73819700	-1.84717600	-0.52263900
H	-2.59348600	-2.57201100	0.28284900
H	-3.81732200	-1.66379800	-0.61116500
H	-2.40781100	-2.31524300	-1.45354400
C	-1.62146600	0.18546400	-2.73678800
H	-0.79297200	0.66631300	-3.26321000
H	-1.63253800	-0.86787600	-3.02912000
H	-2.55233300	0.63957200	-3.10120600
C	-0.39382700	2.73698900	-1.20548900
H	-1.14212500	3.53988200	-1.23592900
H	0.47773400	3.10768300	-0.65925100
H	-0.07510000	2.54350800	-2.23240500
C	-0.76364300	2.27595500	1.92057100
H	-0.53531400	1.77664100	2.86551000
H	0.10746300	2.87292500	1.64044100
H	-1.59738900	2.96644000	2.10450800

C	-2.21021700	-0.55224100	2.35882200
H	-1.55421100	-0.24046300	3.17570600
H	-3.22490500	-0.21128700	2.60415700
H	-2.22632600	-1.64535000	2.34473800
C	1.63728200	-1.60218100	-1.14374600
C	2.16290700	-0.28897700	-1.18199200
H	0.92471800	-3.22149500	0.13860000
C	1.36033100	-2.22961100	0.10287400
C	2.70196600	0.39172400	0.00887600
C	2.14320200	-0.19262200	1.24156900
C	1.61792600	-1.50508700	1.30011200
H	2.28230000	0.36953500	2.15975500
H	1.34048500	-1.93836600	2.25624000
H	1.37430900	-2.10995100	-2.06679800
H	2.31714200	0.19826100	-2.13973500
O	3.36832700	1.43104300	-0.02731700

Int3

-1 1

C	-2.63481300	-0.48428000	2.34287500
C	-1.68543700	-0.56736300	1.29570200

C	-1.49558900	0.53415900	0.38355200
C	-2.56408500	1.49874500	0.32877600
C	-3.52268900	1.57626000	1.36921200
C	-3.54848800	0.60169900	2.39998900
H	-2.69104200	-1.27971700	3.07852400
H	-0.97222700	-1.38615600	1.23111700
H	-2.53000800	2.24686500	-0.45396000
H	-4.26017200	2.37133800	1.35989700
H	-4.29404000	0.65244100	3.18595900
C	-4.13874200	-1.64144300	-1.42238400
C	-4.30131900	-2.50193300	-0.27650000
C	-5.40521700	-2.00516800	0.50279900
C	-5.13883300	-0.60529000	-1.34074200
C	-5.92511300	-0.83071600	-0.15466300
Ru	-3.80831900	-0.45844400	0.43779400
O	-0.48916000	0.59064100	-0.41951600
C	-3.20248400	-1.88942000	-2.56707600
H	-3.03527900	-0.98379200	-3.15043600
H	-2.22561200	-2.25003200	-2.23136700
H	-3.62019200	-2.65073000	-3.23971400

C	-5.39116000	0.45657600	-2.37228800
H	-6.07199400	0.08600500	-3.15067800
H	-5.84781300	1.34573300	-1.92966100
H	-4.46556700	0.76858900	-2.86125400
C	-7.14217100	-0.05445600	0.25877200
H	-7.27492900	-0.05955800	1.34455900
H	-7.08191800	0.98808700	-0.06298400
H	-8.04934100	-0.48442300	-0.18666500
C	-5.99167200	-2.66399400	1.71759600
H	-6.45097100	-1.93505600	2.39141500
H	-6.76941100	-3.38377600	1.42901700
H	-5.23356100	-3.21138700	2.28345500
C	-3.52023500	-3.75584900	-0.01104400
H	-3.48087000	-3.99089300	1.05563700
H	-3.98002900	-4.61101700	-0.52449300
H	-2.49035000	-3.67290600	-0.36777800
N	2.75015000	-1.22344800	-1.62836700
Zr	4.51334300	-1.17126200	-0.60580800
N	1.31814100	-1.80459500	0.75038100
Zr	2.99833000	-2.16302200	1.85648300

N	3.62160800	-0.39732000	1.14129800
Zr	1.88114900	0.09647100	-0.01177000
N	3.81943000	-2.98659500	0.14050700
Zr	2.02296800	-2.92054600	-0.88527000
C	0.33275900	3.48879600	2.65954100
C	1.61662800	2.51280600	2.48446400
C	2.98216700	3.21463300	2.55084600
H	3.16471200	3.64314200	3.54287300
H	3.76195100	2.47353100	2.34825800
H	3.05048900	3.99710800	1.79661000
C	1.63384400	1.32537900	3.45637000
H	2.53460400	0.73093200	3.28151800
H	1.64639100	1.66598800	4.49758000
H	0.76382500	0.68045800	3.31696000
C	0.71501700	4.94529800	2.99358100
H	-0.20141400	5.54049100	3.07399700
H	1.25157600	5.02162700	3.94634600
H	1.32520200	5.37491700	2.19906900
C	-0.67962300	3.00930700	3.71704100
H	-0.27211300	3.03665800	4.73422800

H	-1.54836300	3.67485200	3.68098900
H	-1.02973100	1.99667700	3.50853500
O	1.44832700	2.01350800	1.13461100
O	-0.34300400	3.46571100	1.40962900
B	0.58574900	3.07129000	0.37227600
B	0.13588200	2.30027400	-1.11145000
O	1.26984100	1.62229300	-1.76129600
O	-0.67248000	2.80788000	-2.14255800
C	1.36369200	2.11025200	-3.11788000
C	-0.14867500	2.41274100	-3.42970300
C	2.00416400	1.05226900	-4.01341500
C	2.22904900	3.38151900	-3.09422200
C	-0.91123300	1.16558100	-3.89300300
C	-0.37087300	3.56596700	-4.41047000
H	2.02908100	1.40372200	-5.05198700
H	3.02713100	0.85942400	-3.68242900
H	1.47803000	0.09803100	-3.96668400
H	3.21891800	3.11469000	-2.71309500
H	2.34778400	3.80615800	-4.09722200
H	1.81278900	4.13352300	-2.42123200

H	-1.98167700	1.39826700	-3.89999500
H	-0.62111800	0.85005700	-4.90057800
H	-0.73684900	0.33982400	-3.19998200
H	0.09020200	3.35248800	-5.38156800
H	-1.44425100	3.71420100	-4.56935200
H	0.04295800	4.49774300	-4.02075900
F	1.46831200	4.16166200	0.03608700

TS

-1 1

C	2.64899700	-0.32386000	-1.99237500
C	1.80721900	-0.23590600	-0.85891400
C	1.83182900	0.91875100	0.05085700
C	3.17641100	1.51498300	0.05643500
C	4.02790500	1.46754500	-1.07093900
C	3.77208900	0.54015600	-2.11708800
H	2.49645800	-1.11564600	-2.71929900
H	1.01438400	-0.96371400	-0.71636200
H	3.42279300	2.16378400	0.88927800
H	4.93289400	2.06659900	-1.09372800
H	4.44197700	0.46147200	-2.96584700

C	4.11197400	-1.69476800	1.73414500
C	3.94064200	-2.65766600	0.67438600
C	5.07751100	-2.57078700	-0.20547700
C	5.35371600	-1.00051900	1.48928600
C	5.95183500	-1.54615300	0.29867800
Ru	3.95531200	-0.60971300	-0.19389600
O	1.27345000	0.67771900	1.29419800
C	3.21289900	-1.52757000	2.92414900
H	3.27830800	-0.51760100	3.33408200
H	2.16742600	-1.68744700	2.65422900
H	3.47834800	-2.23829700	3.71998400
C	5.96829100	0.03240200	2.39006700
H	6.58670100	-0.43991200	3.16579300
H	6.60843400	0.72269100	1.83339500
H	5.20097100	0.62492200	2.89419200
C	7.29869200	-1.18405400	-0.25887800
H	7.33433100	-1.32054000	-1.34345400
H	7.55287100	-0.14159800	-0.04830600
H	8.09029400	-1.80963100	0.17738700
C	5.34998300	-3.45987500	-1.38502800

H	5.94067900	-2.94559800	-2.14865500
H	5.90949700	-4.35665800	-1.08426600
H	4.42232100	-3.79659500	-1.85496600
C	2.81388100	-3.64600900	0.57226700
H	2.64853200	-3.96515800	-0.46014400
H	3.03049300	-4.54492100	1.16529700
H	1.87440600	-3.22466500	0.93770000
N	-3.57215500	-0.36578400	1.52463900
Zr	-4.95468300	-0.55204000	-0.01343900
N	-1.64123900	-1.79883700	0.10843700
Zr	-2.97595500	-2.44840300	-1.36445100
N	-3.50017600	-0.52999600	-1.50770700
Zr	-2.09597200	0.20690800	0.00135200
N	-4.43691300	-2.54148200	0.11287600
Zr	-3.02309900	-2.27395900	1.61742500
C	0.38330400	2.60299000	-3.22965600
C	-0.86775900	1.65489400	-2.91782200
C	-2.22811600	2.25863600	-3.28201300
H	-2.29276000	2.44337700	-4.36077700
H	-3.01994600	1.55694000	-3.00567800

H	-2.39724200	3.20134600	-2.75809500
C	-0.75080700	0.26686500	-3.55470700
H	-1.61042800	-0.33506500	-3.25050400
H	-0.76133200	0.33359100	-4.64731800
H	0.16380000	-0.24100500	-3.24435900
C	-0.00299200	4.07816800	-3.47738600
H	0.92068300	4.65961100	-3.56035600
H	-0.57018600	4.20201500	-4.40673500
H	-0.57561700	4.48522700	-2.64580400
C	1.23365900	2.14059600	-4.42162900
H	0.66679000	2.17699500	-5.35870300
H	2.09175800	2.81294200	-4.51961900
H	1.61588300	1.12882600	-4.28246200
O	-0.80933900	1.49179700	-1.45509900
O	1.19060600	2.51286500	-2.05747100
B	0.30872200	2.37686100	-0.93552900
B	0.49330900	1.87277700	1.07296600
O	-0.94537600	1.65184700	1.40210400
O	0.89524600	3.04604000	1.77972400
C	-1.37314800	2.64300800	2.39794400

C	0.01163700	3.25595400	2.87553400
C	-2.15263700	1.92962600	3.50403800
C	-2.29222700	3.65093400	1.69490700
C	0.60050800	2.52962100	4.10334300
C	-0.04692700	4.76067000	3.16061800
H	-2.42316600	2.64632000	4.28883800
H	-3.06379300	1.48551900	3.09807000
H	-1.57068700	1.12382500	3.95523500
H	-3.15727700	3.11555200	1.28642500
H	-2.67077500	4.39628100	2.40212700
H	-1.77404800	4.14683100	0.87599300
H	1.61213500	2.91242000	4.27012600
H	0.01409300	2.69659800	5.01327900
H	0.67705700	1.45431200	3.92501600
H	-0.75511000	4.99236000	3.96455700
H	0.94442100	5.10486100	3.47224200
H	-0.32926000	5.31659500	2.26521100
F	-0.23957000	3.63444000	-0.57867500

TS’

0 1

C	2.77557700	0.32063000	-2.31760300
C	1.70436400	0.20106100	-1.39805100
C	1.34560900	1.34908700	-0.48036400
C	2.69317100	1.75181100	0.06760700
C	3.80128100	1.92660100	-0.79383600
C	3.85306800	1.21078200	-2.02720700
H	2.84651300	-0.34771600	-3.17075600
H	0.95030300	-0.55888700	-1.57655200
H	2.71686800	2.22334800	1.04430500
H	4.66538000	2.49820000	-0.46907500
H	4.70927800	1.29058500	-2.68706200
C	3.31122700	-2.11302800	0.84923700
C	4.13794800	-2.43446100	-0.29422500
C	5.37491800	-1.71176500	-0.16135700
C	4.02280200	-1.17496200	1.66309800
C	5.29846700	-0.91806100	1.03556300
Ru	3.65198600	-0.25181000	-0.35359100
O	0.50187700	0.81081900	0.64608100
C	1.98442100	-2.73747800	1.18165200
H	1.35501500	-2.03651500	1.74002000

H	1.45911800	-3.01412000	0.25642900
H	2.11308900	-3.64668500	1.78569800
C	3.56374400	-0.63045300	2.98533100
H	3.84307000	-1.30471900	3.80669100
H	4.01341200	0.34386100	3.19314500
H	2.47949900	-0.49853100	3.00769300
C	6.40530200	-0.08237700	1.61339400
H	7.05028600	0.32631700	0.83053200
H	6.01357400	0.75965100	2.19052900
H	7.04058000	-0.67406100	2.28682800
C	6.57344900	-1.84076800	-1.05777900
H	7.16016200	-0.91835100	-1.07855000
H	7.23891400	-2.64548400	-0.71576400
H	6.28405600	-2.07115900	-2.08668000
C	3.81884300	-3.46361500	-1.34167900
H	4.35594500	-3.26616400	-2.27337300
H	4.09878000	-4.47287100	-1.00909100
H	2.75095900	-3.47874000	-1.57900500
N	-2.08431600	-1.43507800	1.55449700
Zr	-4.06054900	-1.51673100	0.92436100

N	-1.07950000	-1.68095200	-1.00277400
Zr	-2.93113900	-2.13180600	-1.84219200
N	-3.61816000	-0.53689600	-0.84571200
Zr	-1.68539300	0.04211900	-0.00599600
N	-3.43832200	-3.15987500	-0.09948400
Zr	-1.44397600	-3.01790700	0.52713300
C	-0.45468800	3.60986500	-2.83046700
C	-1.67289900	2.74064300	-2.32766400
C	-2.69729100	3.54608300	-1.52189400
H	-3.25889200	4.22715500	-2.16811400
H	-3.41190900	2.85965000	-1.05659500
H	-2.21179000	4.12694200	-0.73625800
C	-2.38283900	1.94003700	-3.41916700
H	-3.20505000	1.36609000	-2.98178500
H	-2.80205300	2.61493000	-4.17380700
H	-1.70133900	1.24645400	-3.91570200
C	-0.80768200	5.06095200	-3.15990300
H	0.09427500	5.58606200	-3.48664900
H	-1.54364100	5.11610600	-3.96939200
H	-1.20312800	5.58555900	-2.28837800

C	0.28283800	2.97048400	-4.01883500
H	-0.31190400	3.00582400	-4.93742400
H	1.21135600	3.52210300	-4.18851100
H	0.54510100	1.93031200	-3.80830100
O	-1.00877900	1.81021100	-1.41197100
O	0.45250100	3.60282200	-1.71180900
B	0.24696700	2.45716900	-0.94193400
B	-0.04076200	2.07228700	1.16794600
O	-1.44262000	1.85719000	1.48946100
O	0.55566500	2.79573600	2.21381800
C	-1.76501400	2.65522500	2.67002400
C	-0.34351600	2.78910600	3.34438300
C	-2.80760400	1.91275500	3.50281300
C	-2.32525900	3.99715000	2.18772000
C	0.01098900	1.57337100	4.21680400
C	-0.13713900	4.08015400	4.13545200
H	-2.98016000	2.43576300	4.45007100
H	-3.75977900	1.87925400	2.96298500
H	-2.49786200	0.88858900	3.71394700
H	-3.20471200	3.81875100	1.56311900

H	-2.62959200	4.62250400	3.03227600
H	-1.58466900	4.54186800	1.59710500
H	1.07145500	1.62947600	4.47726800
H	-0.56953100	1.55458000	5.14546100
H	-0.15903400	0.63664900	3.67937800
H	-0.85449300	4.15874400	4.95984800
H	0.86953700	4.08624000	4.56313400
H	-0.23468700	4.96019700	3.49744300

Int4

-1 1

C	-2.77345600	0.11663900	2.38541400
C	-1.76022800	-0.00057900	1.40454100
C	-1.44713100	1.17537600	0.50618600
C	-2.83110500	1.50839800	-0.01183800
C	-3.88738900	1.70974900	0.91390400
C	-3.86152300	1.01211500	2.15715900
H	-2.79984900	-0.55532200	3.23816000
H	-0.99983500	-0.76822000	1.52111400
H	-2.91650500	1.96800400	-0.99153500
H	-4.76673400	2.28433600	0.63757200

H	-4.67601900	1.09999100	2.86744200
C	-3.61971500	-1.88338600	-1.22256400
C	-3.73141100	-2.64873500	-0.00543800
C	-5.01846300	-2.38494600	0.58185600
C	-4.83785500	-1.12070300	-1.35994100
C	-5.70270900	-1.44107900	-0.25488300
Ru	-3.76837600	-0.46250700	0.47961500
O	-0.62166900	0.60224500	-0.62610100
C	-2.51050400	-1.97499400	-2.23023700
H	-2.38398800	-1.03286600	-2.76940800
H	-1.55478100	-2.20002800	-1.74992700
H	-2.70660500	-2.76489200	-2.97049600
C	-5.18430800	-0.24260500	-2.52924000
H	-5.63585900	-0.82490000	-3.34496100
H	-5.89786100	0.53808400	-2.24904600
H	-4.29468000	0.25214300	-2.92805200
C	-7.10892300	-0.94824600	-0.06269700
H	-7.37494300	-0.89458200	0.99727500
H	-7.24508800	0.05061200	-0.48703200
H	-7.83758900	-1.61239500	-0.55006800

C	-5.58240700	-3.04812200	1.80634300
H	-6.28359600	-2.39276900	2.33206300
H	-6.12413700	-3.97002500	1.54989900
H	-4.79382000	-3.32139100	2.51274000
C	-2.71749900	-3.63779300	0.49609100
H	-2.79243400	-3.77784900	1.57818700
H	-2.85862500	-4.62055900	0.02501100
H	-1.69696200	-3.31044300	0.28122700
N	2.70588100	-1.13916800	-1.60116000
Zr	4.51126500	-0.85959800	-0.65988400
N	1.43588500	-1.75402600	0.85448300
Zr	3.20913700	-1.91214700	1.89633000
N	3.61994100	-0.12297500	1.11132000
Zr	1.79025000	0.12110900	0.03185000
N	4.05428900	-2.69777100	0.16422100
Zr	2.20874300	-2.85896300	-0.77180000
C	0.29125800	3.60155800	2.80930000
C	1.32194800	2.41925200	2.61621200
C	2.78634400	2.85425000	2.56068100
H	3.07330800	3.33723400	3.50252100

H	3.42999400	1.98557500	2.39997000
H	2.95915900	3.56027600	1.74509200
C	1.13690100	1.31577700	3.66499700
H	1.75859700	0.45589500	3.39735800
H	1.44824800	1.65081200	4.65950100
H	0.09534000	0.98839000	3.70860800
C	0.75283400	4.91426100	2.14276500
H	-0.09012300	5.61210000	2.14430000
H	1.58659400	5.37743200	2.68171500
H	1.04601500	4.74881600	1.10497600
C	-0.06511600	3.88804500	4.27226500
H	0.82033100	4.16567800	4.85548200
H	-0.77269300	4.72234300	4.31255200
H	-0.54050600	3.02425900	4.74050900
O	0.92224000	1.87125200	1.31917600
O	-0.87918000	3.12191100	2.15442100
B	-0.48098100	2.39344000	0.99176200
B	-0.28896000	1.36922000	-1.71329900
O	1.07380500	1.46857200	-1.96929700
O	-1.06236900	1.86270600	-2.72946700

C	1.25686100	2.29848500	-3.15705500
C	-0.17164500	2.17230400	-3.84071200
C	2.41461400	1.73343000	-3.97568200
C	1.58199900	3.71445500	-2.66815400
C	-0.26793700	0.99113800	-4.81544900
C	-0.67107600	3.45344500	-4.50517200
H	2.51298500	2.27447600	-4.92373700
H	3.34720400	1.85160600	-3.41633300
H	2.28848700	0.66904400	-4.17411400
H	2.49831400	3.67455800	-2.07205300
H	1.75229800	4.39234900	-3.51072000
H	0.78756800	4.10698200	-2.03240600
H	-1.31399000	0.85787200	-5.10598200
H	0.32246100	1.16501800	-5.72025500
H	0.07727000	0.06453500	-4.34896400
H	-0.00498900	3.75540400	-5.32058400
H	-1.66624900	3.28114900	-4.92585700
H	-0.74294500	4.27208600	-3.78734000
F	-0.34212300	3.23103100	-0.19260100

C-III

0 1

C	1.44164600	-2.37559300	0.50169500
C	0.69411400	-1.29710500	1.03807900
C	-0.38234400	-0.59850500	0.21028600
C	0.39064800	-0.33907900	-1.07375100
C	1.12642300	-1.37414100	-1.70052900
C	1.65665700	-2.43573500	-0.90839300
H	1.95813500	-3.07250300	1.15467200
H	0.61782800	-1.19127500	2.11642700
H	0.07704200	0.50618200	-1.67944100
H	1.39688100	-1.30218200	-2.74950100
H	2.26330600	-3.21715300	-1.35100500
C	3.40278800	0.98244200	1.26573800
C	4.31334800	-0.11679900	1.03769700
C	4.64974200	-0.13291600	-0.36040800
C	3.16203400	1.62812300	0.01248800
C	3.92183200	0.92886900	-1.00220600
Ru	2.41872000	-0.48441000	-0.12732100
O	-0.61797200	0.66504600	0.89727100
C	2.86344300	1.42522400	2.59586000

H	1.85554900	1.83503300	2.49819300
H	2.81293000	0.59402400	3.30425100
H	3.50448400	2.19838100	3.04125100
C	2.34570200	2.87260300	-0.19158500
H	2.94900100	3.77266100	-0.00837800
H	1.96713300	2.93715500	-1.21552200
H	1.48528900	2.90193400	0.48129000
C	4.05239300	1.34650700	-2.43975100
H	4.29333100	0.49698300	-3.08498500
H	3.12642000	1.79207900	-2.81404100
H	4.84903200	2.09235000	-2.56719500
C	5.65982000	-1.03209100	-1.01478400
H	5.41942500	-1.20864900	-2.06677000
H	6.66564600	-0.59106900	-0.97780000
H	5.71216300	-2.00587300	-0.52006900
C	4.91833600	-0.98870700	2.10152500
H	5.19676900	-1.96999700	1.70685600
H	5.82656400	-0.53371000	2.52018600
H	4.22442200	-1.15155400	2.93091200
C	-3.36615900	-2.79380800	-0.71020200

C	-3.61719500	-2.51269300	0.82363300
C	-5.03266900	-2.06325000	1.18008200
H	-5.76257800	-2.84538900	0.94543700
H	-5.09189100	-1.85650900	2.25258400
H	-5.31227300	-1.15394100	0.64511300
C	-3.18547400	-3.66974400	1.73456500
H	-3.20023800	-3.32649600	2.77265900
H	-3.85722700	-4.52896400	1.64562600
H	-2.16830100	-3.99765600	1.50268900
C	-4.24672000	-1.94638100	-1.63811500
H	-3.88867200	-2.06202000	-2.66496300
H	-5.29461000	-2.25980500	-1.60172200
H	-4.18168000	-0.88689700	-1.37783400
C	-3.42567400	-4.26351800	-1.12116000
H	-4.42581400	-4.67653400	-0.95150700
H	-3.19929600	-4.35382400	-2.18741000
H	-2.70078100	-4.86668300	-0.57181700
O	-2.70367900	-1.41124900	1.09204500
O	-2.00199500	-2.31946900	-0.89240600
B	-1.73623500	-1.43137300	0.11762700

B	-1.54839600	1.54561200	0.47194900
O	-1.72591000	2.76276100	1.09830700
O	-2.39623700	1.36468900	-0.61054900
C	-2.94629400	3.33460900	0.56542600
C	-3.05364000	2.63913000	-0.84857800
C	-2.79717500	4.85411800	0.53090500
C	-4.08118500	2.93347000	1.51889900
C	-2.24830100	3.36036200	-1.93814700
C	-4.47645000	2.37495000	-1.33587300
H	-3.67436600	5.32385300	0.07328500
H	-2.70368800	5.23552400	1.55178600
H	-1.90765700	5.15852700	-0.02351800
H	-3.83093800	3.27631200	2.52652000
H	-5.03628600	3.38218500	1.22883600
H	-4.20012800	1.84665000	1.55366500
H	-2.19217100	2.71743700	-2.82110000
H	-2.71583600	4.30561500	-2.23004000
H	-1.22807700	3.56770300	-1.60291700
H	-5.02478400	3.31425400	-1.46423900
H	-4.44644500	1.86781200	-2.30485000

H	-5.02966600	1.74260200	-0.63909500

C-IV

1 1

C	1.26563100	2.29595200	-1.33445700
C	0.00522800	1.64748200	-1.28887700
C	-0.65147200	1.39803300	-0.04615800
C	0.00334200	1.81018600	1.15217100
C	1.26946500	2.44558600	1.11507400
C	1.90376000	2.69007800	-0.13015300
H	1.75943400	2.45691700	-2.28617900
H	-0.46106000	1.31009200	-2.20773500
H	-0.46799300	1.60627100	2.10718000
H	1.76609700	2.72058300	2.03870300
H	2.88251700	3.15514400	-0.15996300
C	1.27215500	-1.72472300	0.48912400
C	1.61863600	-1.58605000	-0.90403200
C	2.90649600	-0.94235800	-0.97809100
C	2.33914900	-1.16073000	1.27532900
C	3.34937000	-0.67527100	0.36865000
Ru	1.45656000	0.43887300	-0.00664300

C	0.06552100	-2.43995400	1.01771800
H	-0.23711600	-2.05998500	1.99607600
H	-0.78225200	-2.34751300	0.33549200
H	0.28470100	-3.50887200	1.13206800
C	2.43565300	-1.18180500	2.77192800
H	2.92178900	-2.10753100	3.10372300
H	3.02747200	-0.34642900	3.15252700
H	1.45073800	-1.14132600	3.24242300
C	4.68197900	-0.10900800	0.76046000
H	5.07012200	0.57803400	0.00529500
H	4.63267000	0.42478400	1.71214800
H	5.41366000	-0.91826800	0.87515500
C	3.69938400	-0.69575100	-2.22726400
H	4.35759100	0.17013200	-2.12664100
H	4.33131700	-1.56509800	-2.44675100
H	3.05405900	-0.53297000	-3.09316500
C	0.81594800	-2.11290700	-2.05714100
H	1.08186100	-1.61648600	-2.99272400
H	1.00365600	-3.18563900	-2.18829700
H	-0.25635100	-1.97633500	-1.89717000

C	-4.06588200	-0.04133600	0.77905000
C	-3.94077700	-0.32708800	-0.77523800
C	-4.28703700	-1.74941600	-1.20333200
H	-5.33904400	-1.96788500	-0.99597800
H	-4.12891500	-1.85820400	-2.27966600
H	-3.67365800	-2.49324200	-0.69175500
C	-4.68483100	0.68730200	-1.64988700
H	-4.40174700	0.53385400	-2.69451200
H	-5.76804900	0.56445500	-1.56909500
H	-4.43435700	1.71653600	-1.37780100
C	-3.87652500	-1.28165400	1.65758100
H	-3.79310600	-0.96885100	2.70170600
H	-4.72804100	-1.96216600	1.57373500
H	-2.96934300	-1.83180100	1.39392400
C	-5.32538300	0.71201400	1.19798600
H	-6.21988900	0.12274700	0.97380900
H	-5.30307300	0.89210600	2.27595700
H	-5.40925400	1.67706300	0.69633800
O	-2.50458300	-0.10502800	-1.02508900
O	-2.90880200	0.83077600	1.03412500

B	-2.04897100	0.67839500	-0.00629500

Product

0 1

C	3.79391300	1.19949600	-0.15704600
C	2.39973600	1.19641300	-0.15580700
C	1.67746400	0.00000200	0.00000800
C	2.39973500	-1.19641100	0.15581600
C	3.79391300	-1.19949600	0.15704200
C	4.49242900	0.00000000	-0.00000500
H	4.33690600	2.13262000	-0.27915700
H	1.85651100	2.12958700	-0.27638300
H	1.85651000	-2.12958400	0.27639600
H	4.33690500	-2.13262100	0.27914700
H	5.57910100	-0.00000100	-0.00001000
C	-2.01930300	-0.78576200	-0.08073400
C	-2.01930700	0.78576100	0.08073100
C	-2.92056000	1.54070400	-0.89373900
H	-3.97085100	1.26933200	-0.74449800
H	-2.82251700	2.61668000	-0.72425400
H	-2.65170300	1.33824800	-1.93171300

C	-2.29001700	1.25026800	1.51803300
H	-2.07060200	2.31879900	1.59146900
H	-3.33399000	1.09253600	1.80460500
H	-1.65025900	0.72498100	2.23248600
C	-2.28999000	-1.25026900	-1.51804000
H	-2.07057500	-2.31880000	-1.59147300
H	-3.33395900	-1.09253500	-1.80462800
H	-1.65022000	-0.72498100	-2.23248300
C	-2.92056800	-1.54071000	0.89372100
H	-3.97085800	-1.26933800	0.74446700
H	-2.82252200	-2.61668500	0.72423500
H	-2.65172500	-1.33825600	1.93170000
O	-0.63565500	1.12649000	-0.21404500
O	-0.63565400	-1.12648700	0.21406200
B	0.12392900	0.00000300	0.00001600
